# TGFβ signalling: a nexus between inflammation, placental health and preeclampsia throughout pregnancy

**DOI:** 10.1093/humupd/dmae007

**Published:** 2024-03-22

**Authors:** Monika Horvat Mercnik, Carolin Schliefsteiner, Gonzalo Sanchez-Duffhues, Christian Wadsack

**Affiliations:** Department of Obstetrics and Gynaecology, Medical University of Graz, Graz, Austria; Department of Obstetrics and Gynaecology, Medical University of Graz, Graz, Austria; Nanomaterials and Nanotechnology Research Center (CINN-CSIC), Tissue-Specific BMP Signalling ISPA-HUCA, Oviedo, Spain; Department of Obstetrics and Gynaecology, Medical University of Graz, Graz, Austria; BioTechMed-Graz, Graz, Austria

**Keywords:** preeclampsia, transforming growth factor, bone morphogenetic protein, TGFβ, signalling, trophoblast, endothelial cells, macrophages, human placenta, immune cells

## Abstract

**BACKGROUND:**

The placenta is a unique and pivotal organ in reproduction, controlling crucial growth and cell differentiation processes that ensure a successful pregnancy. Placental development is a tightly regulated and dynamic process, in which the transforming growth factor beta (TGFβ) superfamily plays a central role. This family of pleiotropic growth factors is heavily involved in regulating various aspects of reproductive biology, particularly in trophoblast differentiation during the first trimester of pregnancy. TGFβ signalling precisely regulates trophoblast invasion and the cell transition from cytotrophoblasts to extravillous trophoblasts, which is an epithelial-to-mesenchymal transition-like process. Later in pregnancy, TGFβ signalling ensures proper vascularization and angiogenesis in placental endothelial cells. Beyond its role in trophoblasts and endothelial cells, TGFβ signalling contributes to the polarization and function of placental and decidual macrophages by promoting maternal tolerance of the semi-allogeneic foetus. Disturbances in early placental development have been associated with several pregnancy complications, including preeclampsia (PE) which is one of the severe complications. Emerging evidence suggests that TGFβ is involved in the pathogenesis of PE, thereby offering a potential target for intervention in the human placenta.

**OBJECTIVE AND RATIONALE:**

This comprehensive review aims to explore and elucidate the roles of the major members of the TGFβ superfamily, including TGFβs, bone morphogenetic proteins (BMPs), activins, inhibins, nodals, and growth differentiation factors (GDFs), in the context of placental development and function. The review focusses on their interactions within the major cell types of the placenta, namely trophoblasts, endothelial cells, and immune cells, in both normal pregnancies and pregnancies complicated by PE throughout pregnancy.

**SEARCH METHODS:**

A literature search was carried out using PubMed and Google Scholar, searching terms: ‘TGF signalling preeclampsia’, ‘pregnancy TGF signalling’, ‘preeclampsia tgfβ’, ‘preeclampsia bmp’, ‘preeclampsia gdf’, ‘preeclampsia activin’, ‘endoglin preeclampsia’, ‘endoglin pregnancy’, ‘tgfβ signalling pregnancy’, ‘bmp signalling pregnancy’, ‘gdf signalling pregnancy’, ‘activin signalling pregnancy’, ‘Hofbauer cell tgfβ signalling’, ‘placental macrophages tgfβ’, ‘endothelial cells tgfβ’, ‘endothelium tgfβ signalling’, ‘trophoblast invasion tgfβ signalling’, ‘trophoblast invasion Smad’, ‘trophoblast invasion bmp’, ‘trophoblast invasion tgfβ’, ‘tgfβ preeclampsia’, ‘tgfβ placental development’, ‘TGFβ placental function’, ‘endothelial dysfunction preeclampsia tgfβ signalling’, ‘vascular remodelling placenta TGFβ’, ‘inflammation pregnancy tgfβ’, ‘immune response pregnancy tgfβ’, ‘immune tolerance pregnancy tgfβ’, ‘TGFβ pregnancy NK cells’, ‘bmp pregnancy NK cells’, ‘bmp pregnancy tregs’, ‘tgfβ pregnancy tregs’, ‘TGFβ placenta NK cells’, ‘TGFβ placenta tregs’, ‘NK cells preeclampsia’, ‘Tregs preeclampsia’. Only articles published in English until 2023 were used.

**OUTCOMES:**

A comprehensive understanding of TGFβ signalling and its role in regulating interconnected cell functions of the main placental cell types provides valuable insights into the processes essential for successful placental development and growth of the foetus during pregnancy. By orchestrating trophoblast invasion, vascularization, immune tolerance, and tissue remodelling, TGFβ ligands contribute to the proper functioning of a healthy maternal–foetal interface. However, dysregulation of TGFβ signalling has been implicated in the pathogenesis of PE, where the shallow trophoblast invasion, defective vascular remodelling, decreased uteroplacental perfusion, and endothelial cell and immune dysfunction observed in PE, are all affected by an altered TGFβ signalling.

**WIDER IMPLICATIONS:**

The dysregulation of TGFβ signalling in PE has important implications for research and clinical practice. Further investigation is required to understand the underlying mechanisms, including the role of different ligands and their regulation under pathophysiological conditions, in order to discover new therapeutic targets. Distinguishing between clinically manifested subtypes of PE and studying TGFβ signalling in different placental cell types holistically is an important first step. To put this knowledge into practice, pre-clinical animal models combined with new technologies are needed. This may also lead to improved human research models and identify potential therapeutic targets, ultimately improving outcomes for affected pregnancies and reducing the burden of PE.

## Graphical Abstract

**Graphical Abstract:**
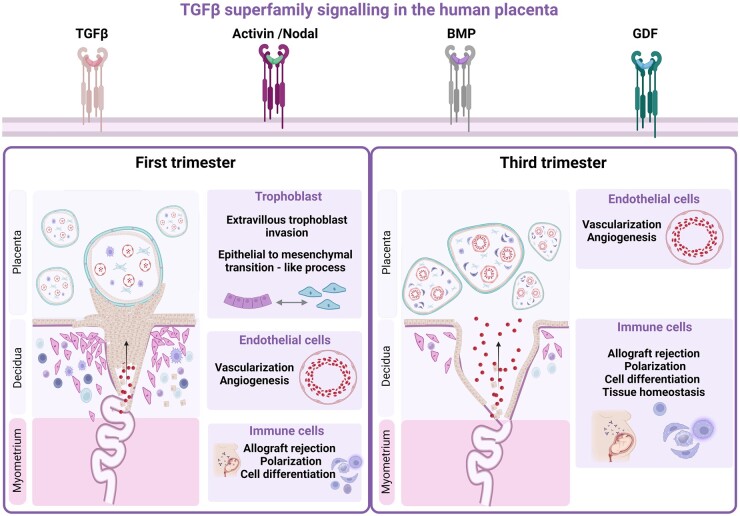
TGFβ signalling governs essential processes in placental development, coordinating trophoblast invasion, vascularization, immune tolerance, and tissue remodelling across cell types, to ensure a healthy pregnancy outcome.

## Introduction

**Figure 1.**
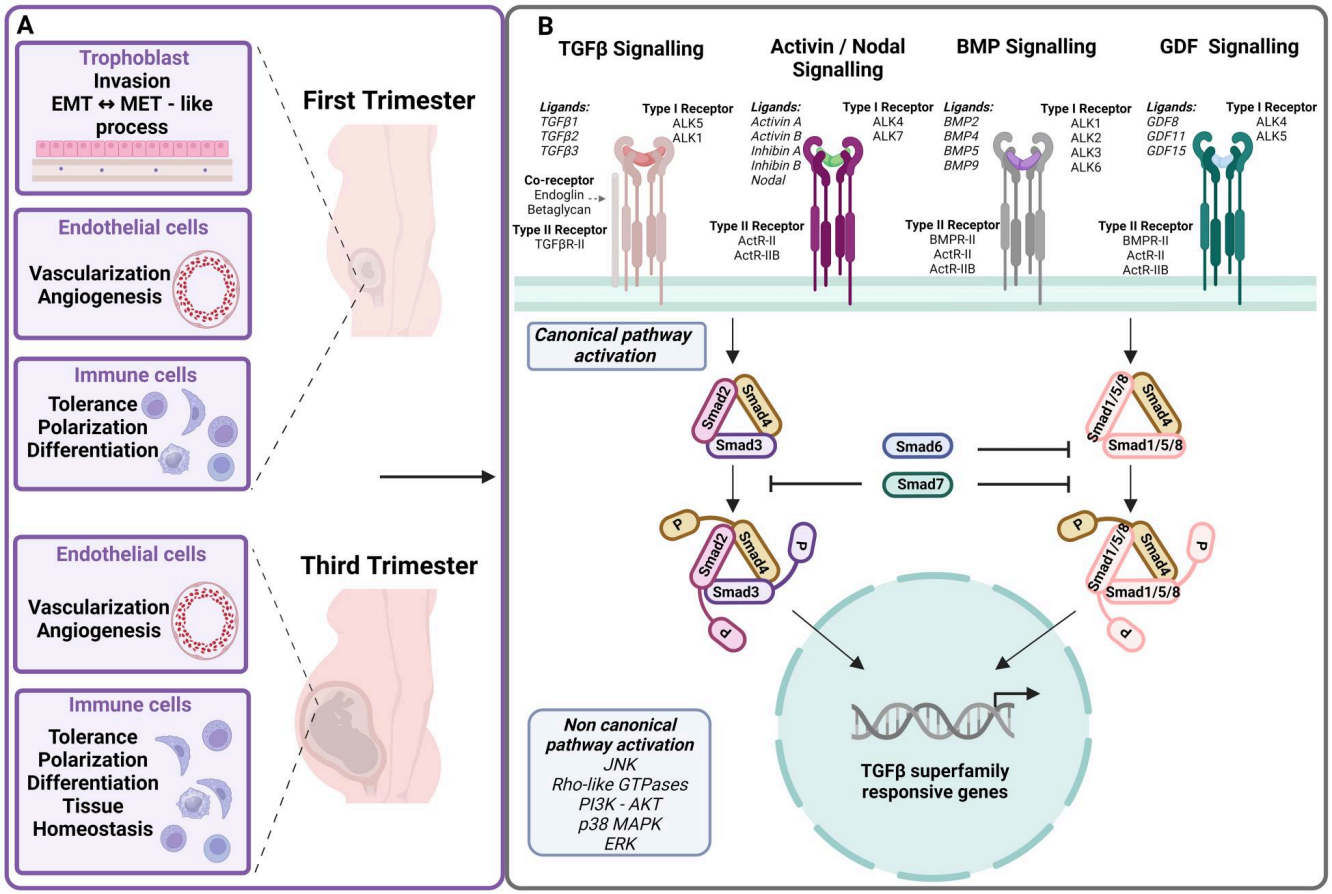
**TGFβ signalling in the placenta.** (**A**) TGFβ signalling in the first and third trimester placenta. TGFβ signalling is crucial for placental development and the functional regulation of placental cells. In trophoblasts, particularly during the first trimester, TGFβ signalling is involved in regulating cell invasion and the transition of CTs to EVTs through an EMT-like process. Additionally, some ligands of the TGFβ family stimulate the reverse process called MET. To ensure blood supply and placental function, TGFβ signalling contributes to vascularization by stimulating angiogenesis through placental ECs. During pregnancy, TGFβ signalling contributes to the establishment of the maternal tolerance to the semi-allogeneic foetus by limiting the immunogenic response. TGFβ also plays a central role in Tregs differentiation, regulation of NK cells functions, and the balance between M1 and M2 macrophages. (**B**) TGFβ family signalling activation pathways in the human placenta. TGFβ ligands interact with type I and type II receptors and induce downstream signalling through canonical and non-canonical pathways. Upon ligand-receptor binding, the receptor complex becomes phosphorylated and recruits the canonical intracellular mediators Smad2/3 or Smad1/5/8. Depending on the type I receptor activated, in the canonical signalling route, either Smad2/3 or Smad1/5/8 are trans-phosphorylated. TGFβ and Activins via ALK4/5/7 primarily activate Smad2/3, whereas Smad1/5/8 activation is induced by the BMP receptors ALK1/2/3/6. Of note, on certain occasions, TGFβ s, activins, and GDFs can activate both Smad1/5/8 and Smad2/3 signalling. Phosphorylated Smads form complexes with Smad4 and translocate to the nucleus to regulate gene expression. Inhibitory Smads, such as Smad6 and Smad7, act as negative feedback regulators to balance receptor activity. In addition to the canonical pathways, TGFβ ligand binding can activate non-canonical, Smad-independent signalling pathways. These pathways involve downstream effectors such as JNK, Rho-like GTPases, PI3K-AKT, p38 MAPK, and ERK. Non-canonical signalling regulates TGFβ responsive genes and mediates additional cellular responses.

During pregnancy, the human placenta plays a unique role as the first organ to develop from cells originating from the blastocyst. The beginning of a healthy pregnancy relies on the intricate regulation between trophoblast cell invasion and foeto-maternal tolerance (Knöfler *et al.*, 2019). As the pregnancy advances, the successful maintenance of the pregnancy relies on the precise coordination, action, and interplay of various cell types in a highly orchestrated manner, to support the developing foetus and ensure a healthy pregnancy outcome (Huppertz, 2008a; Cindrova-Davies and Sferruzzi-Perri, 2022). TGFβ signalling plays a major part in embryonic and placental development and thereby effecting pregnancy outcome (Fig. 1) (Haider *et al.*, 2017, 2022; Li *et al.*, 2021; Yang *et al.*, 2021; Fang *et al.*, 2022). In the first trimester of pregnancy, TGFβ signalling is primarily involved in the regulation of trophoblast invasion, remodelling of the spiral arteries, and development of the placental vasculature (Dietrich *et al.*, 2022; Li *et al.*, 2023a). Disturbances in TGFβ levels in maternal plasma have been associated with miscarriages (Ogasawara *et al.*, 2000; Dirisipam *et al.*, 2023), but TGFβ levels in the placental bed do not seem to be altered under these circumstances (Ball *et al.*, 2007). Emerging evidence suggest that various components of the TGFβ pathways have been frequently reported as altered in the pathogenesis of preeclampsia (PE) (Venkatesha *et al.*, 2006; Haider *et al.*, 2017; Zhao *et al.*, 2020b; Deng *et al.*, 2023), and might be potential therapeutic targets for intervention. As the pregnancy progresses, TGFβs take up the role as immune suppressors, promoting maternal tolerance of the semi-allogenic allograft during gestation (Ingman and Robertson, 2009). Additionally, TGFβ family members are involved in regulating the function of placental endothelial cells (ECs) (Zhou *et al.*, 2019) and immune cells (Keskin *et al.*, 2007; Mercnik *et al.*, 2022; Vondra *et al.*, 2023). This comprehensive review highlights the TGFβ superfamily signalling and its key ligands, such as transforming growth factor β (TGFβ), bone morphogenetic proteins (BMPs), activins, inhibins, nodals, and growth differentiation factors (GDFs), in the context of placental function in normal and PE-compromised pregnancies. In consequence, all major cell types of the early and late placenta namely, trophoblasts, ECs, and immune cells, are the subject of these summaries. By highlighting the indispensable role of TGFβ family members, this review emphasizes the inherent biological diversity of placental cells and related cell models, which are, in addition, influenced by non-placental cell types that produce vital TGFβ ligands. While placental cells are the primary source of TGFβ ligands (Jones *et al.*, 2006b; Chuva de Sousa Lopes *et al.*, 2020), it is also crucial to note the involvement of other non-placental factors. For example, the release of TGFβ ligands from foetal or maternal tissues, that is the liver (Bidart *et al.*, 2012) or the endometrium (Jones *et al.*, 2006b), thereby, contributing to the complex network of TGFβ signalling in the human placenta (Table 1). In detail, this substantial contribution of TGFβ family ligands from maternal and foetal cell types beyond the placenta, influencing the maternal–placental interface, is essential for successful pregnancy initiation, and involves cellular interactions and networks, thereby intricately shaping placental function and development. Accounting for ligand bioavailability broadens the signalling window through the TGFβ receptor family, adding complexity to the understanding of the TGFβ cellular network and highlighting the critical importance of TGFβ signalling in the context of pregnancy.

**Table 1.** Tissue sources of TGFβ family ligands.

TGFβ superfamily	Ligand	Compartment	Tissue/cell type	Reference
TGFβ	TGFβ1	Placenta	CT	Lash *et al.*, 2005; Jones *et al.*, 2006b; Forbes *et al.*, 2010
			EVT	Jones *et al.*, 2006b; Ma *et al.*, 2020b; Arutyunyan *et al.*, 2023; Vondra *et al.*, 2023
			ST	Jones *et al.*, 2006b
			dNK	Lash *et al.*, 2006; Fraser *et al.*, 2012
			dM	Ning *et al.*, 2016; Wang *et al.*, 2022
			HBC	Schliefsteiner *et al.*, 2017, 2020; Pavlov *et al.*, 2020; Azari *et al.*, 2021; Mercnik *et al.*, 2022
		Maternal tissue	Decidua, Endometrium, Uterus	Jones *et al.*, 2006b; Zhang *et al.*, 2019; Yang *et al.*, 2021
	TGFβ2	Placenta	CT	Jones *et al.*, 2006b; Lash *et al.*, 2005
			EVT	Lyall *et al.*, 2001
			dM	Vondra *et al.*, 2023
			HBC	Pavlov *et al.*, 2020
		Maternal tissue	Decidua, Endometrium, Uterus	Simpson *et al.*, 2002
	TGFβ3	Placenta	EVT	Caniggia *et al.*, 1999
			HBC	Pavlov *et al.*, 2020
		Maternal tissue	Decidua, Endometrium, Uterus	Jones *et al.*, 2006b; Simpson *et al.*, 2002
BMP	BMP2	Placenta	EVT	Yi *et al.*, 2021
			HBC	Deng *et al.*, 2023
		Maternal tissue	Decidua	Yi *et al.*, 2021
	BMP4	Foetal tissue	Extraembryonic ectoderm	Roberts *et al.*, 2022
	BMP9	Maternal tissue	Liver	Bidart *et al.*, 2012
NODAL	NODAL	Maternal tissue	Uterus	Park *et al.*, 2012
ACTIVIN/INHIBIN	ACTIVIN A	Placenta	CT	Debieve *et al.*, 2000; Bearfield *et al.*, 2005; Mylonas *et al.*, 2006; Barber *et al.*, 2023
		Maternal tissue	Decidua	Keelan *et al.*, 1998
		Foetal tissue	Foetal membranes	Petraglia *et al.*, 1993; Keelan *et al.*, 1998; Barber *et al.*, 2023
	ACTIVIN B	Placenta	CT	Jones *et al.*, 2006a,b; Adu-Gyamfi *et al.*, 2020
	INHIBIN A	Placenta	CT	Debieve *et al.*, 2000; Bearfield *et al.*, 2005
		Maternal tissue	Decidua	Riley *et al.*, 2000
		Foetal tissue	Foetal membranes	Petraglia *et al.*, 1993; Riley *et al.*, 2000
	INHIBIN B	Placenta	CT	Mylonas *et al.*, 2006
			ST	Mylonas *et al.*, 2006
		Maternal tissue	Decidua	Riley *et al.*, 2000
		Foetal tissue	Foetal membranes	Riley *et al.*, 2000
GDF	GDF8	Placenta	CT	Peiris & Mitchell, 2012
			ST	Peiris & Mitchell, 2012
			EVT	Peiris *et al.*, 2014; Xie *et al.*, 2020
		Maternal tissue	Uterus, Endometrium	Peiris & Mitchell, 2012
	GDF11	Placenta	CT	Sugulle *et al.*, 2009
			ST	Sugulle *et al.*, 2009
	GDF15	Placenta	CT	Sugulle *et al.*, 2009
			ST	Marjono *et al.*, 2003; Jones *et al.*, 2006b; Sugulle *et al.*, 2009; Zeng *et al.*, 2023
			EVT	Segerer *et al.*, 2012; Zeng *et al.*, 2023
		Maternal tissues	Decidua	Marjono *et al.*, 2003; Segerer *et al.*, 2012

CT, cytotrophoblast; EVT, extravillous trophoblasts; ST, syncytiotrophoblast; dNK, decidual natural killer cells; dM, decidual macrophages; HBC, Hofbauer cells.

## Transforming growth factor beta signalling

Transforming growth factor beta (TGFβ) signalling is vital in tissue and cell homeostasis, regulating inflammatory and cellular processes such as proliferation, growth, differentiation, apoptosis, migration, or matrix formation (Moustakas *et al.*, 2002; Tzavlaki and Moustakas, 2020). Since the discovery of the first ligand namely TGFβ1 (Assoian *et al.*, 1983), more than thirty ligands have been described (Derynck and Budi, 2019). The characterization of TGFβ ligands has been based on structure and phylogeny (Heldin and Moustakas, 2016), sub-stratifying them into six major subfamilies: the TGFβ isoforms, BMPs, nodals, anti-Müllerian hormone (AMH), activins/inhibins, and GDFs (Derynck and Budi, 2019). This review focuses on the TGFβ subfamilies and their roles in healthy and preeclamptic placentas (Fig. 1). While AMH is vital for the ovarian reserve and for detecting polycystic ovary syndrome (Rudnicka *et al.*, 2021), its role in placental development remains unclear. Although AMH and its receptor are present in placental tissue (Novembri *et al.*, 2015), their direct significance and function in this context are yet to be understood, and therefore, this review will not delve into it.

All members of the TGFβ family act as homo- or heterodimers, in most cases formed through a disulfide bond (Heldin and Moustakas, 2016). Activation of the TGFβ superfamily begins with ligand dimer binding to transmembrane serine–threonine kinase receptors, which are divided into type I and type II classes. There are seven type I receptors, known as activin receptor-like kinases (ALK1–ALK7) and five type II receptors: activin type II receptor A and B (ActRIIA, ActRIIB), BMP receptor II (BMPRII), transforming growth factor β receptor II (TGFβRII), and anti-Müllerian hormone receptor II (AMHRII) (Sierra-Filardi *et al.*, 2011; Heldin and Moustakas, 2016; Derynck and Budi, 2019; Tzavlaki and Moustakas, 2020). Co-receptors such as endoglin (Eng), BMP and activin membrane-bound inhibitor (BAMBI), betaglycan (TGFΒRIII), cripto, CD109, repulsive guidance molecule b (RGMb), and neuropilin-1 further affect the signalling (Vogt *et al.*, 2011; Tzavlaki and Moustakas, 2020).

### Canonical Smad pathway

TGFβ family members exhibit the conventional activation of canonical Smad (derived from the fusion of the Caenorhabditis elegans Sma genes and the Drosophila Mad—Mothers against decapentaplegic—genes) mediated pathways (Moustakas and Heldin, 2009; Derynck and Budi, 2019; Tzavlaki and Moustakas, 2020). The Smad family consist of eight Smad proteins, which are divided into three distinct subgroups: receptor-activated Smads (R-Smads: Smad1, Smad2, Smad3, Smad5, and Smad8), the common Smad (C-Smad4), and inhibitory Smads (I-Smads: Smad6 and Smad7) (Heldin *et al.*, 1997; Wrighton *et al.*, 2009).

To initiate the signalling cascade, a dimeric TGFβ ligand interacts with the specific type I and type II receptors to form a heterotetrameric complex. Upon a complex formation, type II receptors activate the type I receptor by trans-phosphorylation (Heldin and Moustakas, 2016). This phosphorylation occurs in the juxtamembrane domain of the type I receptor and activates its kinase function (Moustakas and Heldin, 2009; Heldin and Moustakas, 2016; Yan *et al.*, 2018). Followed by a successful receptor activation, the R-Smads are recruited and activated by the type I receptor to form cytosolic heteromeric trimers together with Smad4 (Tzavlaki and Moustakas, 2020). Smad4 functions as a cofactor that enhances ligand-induced transcription by stabilizing the interaction of R-Smads with DNA (Hill, 2016; Kamato *et al.*, 2020). Although Smad4 is not essential for nuclear transport, it is often translocated together with R-Smads. In the nucleus, Smads form transcriptional complexes with specific cofactors and regulatory proteins, thereby influencing the expression of target genes (Miyazono *et al.*, 2005). TGFβ ligands can activate both the Smad2/3 and Smad1/5/8 pathways in a context-dependent manner. In particular, Smad1, Smad5, and Smad8 are primarily R-Smads activated by BMP type I receptors, whereas Smad2 and Smad3 are known to be activated by activin, GDF, and TGFβ type I receptors (Rochette *et al.*, 2020). However, it is important to emphasize that activation of the Smad2/3 or the Smad1/5/8 pathway is context dependent and varies according to ligand binding, receptor activation, and conditions (Heldin and Moustakas, 2016; Rochette *et al.*, 2020; Tzavlaki and Moustakas, 2020).

Post-translational modifications of Smads fine-tune the responses to TGFβ ligands. Phosphorylation, ubiquitination, sumoylation, and other modifications occur to control Smad stability and function. In the nucleus, R-Smads are constantly dephosphorylated, leading to dissociation of Smad complexes and the export of inactive Smads to the cytoplasm (Derynck and Zhang, 2003; Vogt *et al.*, 2011). Moreover, Smad signalling is regulated by positive and negative regulators, including I-Smads, the co-repressors Ski and SnoN and the Smurf family of E3 ubiquitin ligases (Luo, 2017). The I-Smads are strongly induced by TGFβ receptor activation, and their induction provides an auto-inhibitory feedback mechanism for ligand-induced signalling (Yan *et al.*, 2016). Smad6 and Smad7 compete with R-Smads for receptor interaction. They can also induce receptor degradation or interfere with Smad DNA binding (Yan *et al.*, 2009, 2016). In addition, Smads are involved in crosstalk with other signalling pathways, such as the Wingless-related integration site (Wnt), Hippo, Notch, Hedgehog, and Nuclear Factor κB (NF-κB) pathways, often in a cell type-specific manner, which plays an important role in the regulation of various biological responses (Luo, 2017).

### Non-canonical pathway

In addition to canonical Smad signalling, TGFβ-related ligands can also regulate other signalling pathways, that may influence both Smad-mediated and Smad-independent responses (Vogt *et al.*, 2011). Smad-independent signalling or non-canonical TGFβ signalling involves the activation of several downstream pathways, including: c-JUN N-terminal kinase (JNK), Rho-like GTPases, phosphatidylinositol 3-kinase (PI3K)—protein kinase B (Akt), p38 mitogen-activated kinase (MAPK), and extracellular signal-regulated kinase (ERK) MAPK (Luo, 2017; van Caam *et al.*, 2017). The MAPK and Smad pathways often directly or reciprocally regulate each other’s activities and output. For example, ERK and GSK3b kinases can phosphorylate the R-Smads in the linker region, to either enhance or diminish Smad mediated transcriptional activation (Fuentealba *et al.*, 2007; Hough *et al.*, 2012). Moreover, activation of non-canonical signalling leads to the differential recruitment of cofactors to transcriptional complexes containing the Smads, thereby influencing the selectivity, potency, and longevity of Smads-induced gene expression (Abdollah *et al.*, 1997; Derynck and Budi, 2019). TGFβRI mediated phosphorylation of TAK1 (TGFβ activated kinase 1), which is an important mediator of p38 MAPK pathway, has been implicated in the regulation of apoptosis, cell migration, and cell differentiation (Wendt *et al.*, 2009; Derada Troletti *et al.*, 2019). TGFβ family ligands induce a rapid activation of Rho-like GTPases resulting in control of cytoskeletal dynamics and cell motility (Derynck and Zhang, 2003; Derynck and Budi, 2019). Moreover, TGFβ can indirectly activate PI3K-Akt signalling through miRNAs (Zhang, 2017). In turn, inhibition of PI3K-Akt signalling has been shown to prevent TGFβ-induced Smad3 activation and the association with Smad4 (Runyan *et al.*, 2004). PI3K-Akt signalling has been shown to antagonize TGFβ/Smad signalling to promote cell survival through an Akt kinase-dependent mechanism (Zhang, 2017).

### TGFβ superfamily ligands

#### TGFβ subfamily

TGFβ exists in three known isoforms: TGFβ1, TGFβ2, and TGFβ3, all of which are secreted into the extracellular space in an inactive, latent form (Jenkins, 2008). The TGFβ isoforms contain highly conserved regions but differ in several amino acid sequences (Kubiczkova *et al.*, 2012; Poniatowski *et al.*, 2015). Despite sequence similarities, the isoforms bind differently to the receptors. TGFβ2 binds to the TGFβRII through different residues than TGFβ1 and TGFβ3, and presentation of TGFβ2 to the receptor requires the presence of a co-receptor, either betaglycan or Eng (Heldin and Moustakas, 2016), whereas TGFβ1 and TGFβ3 can bind directly to TGFβRII (Laverty *et al.*, 2009; Huang *et al.*, 2014). Although the isoforms signal via similar signalling pathways, the cellular effect and outcome may differ. TGFβ1 is the most abundant and ubiquitously expressed isoform, and is predominantly expressed in the cells of the immune system, where it acts as a potent immunoregulator (Yoshimura *et al.*, 2010; Kubiczkova *et al.*, 2012). Activation of latent TGFβ is an important checkpoint of TGFβ bioavailability (Jenkins, 2008). To prevent access to TGFβ receptors, ligands are synthesized as precursors (18) consisting of a pro-domain, called latency associated polypeptide (LAP) and the active TGFβ. The precursors can be cleaved into mature dimeric proteins, often linked by disulfide bonds during processing through the secretory pathway. Proteases play a role in the process of indirect or direct activation of extracellular TGFβ. Some matrix metalloproteinases (MMPs), such as MMP2 and MMP9 can directly cleave and activate latent TGFβ, whereas membrane-type matrix metalloproteinase (MT-MMP) interacts with integrin-mediated TGFβ activation pathways (Jenkins, 2008). Active TGFβ is involved in the synthesis of many of its own activators, such as thrombospodin-1 (TSP-1), furins, and metalloproteinases (De Caestecker *et al.*, 1998), creating autocrine feedback loops in TGFβ bioavailability and signalling.

Upon activation of the ligands, they bind to their respective receptors. TGFΒRII is primarily involved in the basic signal transduction of the TGFβ subfamily as a type II receptor (Moustakas and Heldin, 2009; Heldin and Moustakas, 2016). In most cell types, TGFβ signals via ALK5 or TGFβRI as a type I receptor, whereas in ECs signal transduction can occur via both ALK5 and ALK1 (Goumans *et al.*, 2002, 2003). Signalling via ALK5 activates Smad2/3 signalling (Tzavlaki and Moustakas, 2020), which is considered the most canonical TGFβ signalling, while stimulation of ALK1 leads to activation of Smad1/5/8 (Goumans *et al.*, 2003). The coreceptor Eng is required for TGFβ signalling via ALK1. Other coreceptors, like betaglycan or TGFΒRIII, may also regulate signalling by either facilitating or inhibiting ligand–receptor interaction, adding another layer of regulation and specificity to the signalling cascade (Heldin *et al.*, 1997; Moustakas and Heldin, 2009; Heldin and Moustakas, 2016; Tzavlaki and Moustakas, 2020).

#### BMP subfamily

Since their discovery as ectopic bone inducers, BMPs have been shown to affect a wide variety of cell types and processes beyond bone physiology (Wang *et al.*, 2014). They are important morphogens in embryogenesis and development and have also been shown to regulate adult tissue homeostasis (Wang *et al.*, 2014). Many processes in early development depend on BMP signalling gradients for cell growth, apoptosis, and differentiation (Zou and Niswander, 1996; Kobayashi *et al.*, 2005; Jones *et al.*, 2006b; Stewart *et al.*, 2010; Wang *et al.*, 2014). Based on the sequence similarity and their function, BMPs are divided into several subgroups: (i) BMP2/4, (ii) BMP5/6/7/8a/8b, (iii) BMP9/10, and (iv) BMP12/13/14 (Bragdon *et al.*, 2011). Normally, BMPs are synthesized as pro-peptides, and after secretion and cleavage, BMPs bind to either the extracellular matrix, soluble antagonists, co-receptors, or transmembrane serine/threonine kinase receptors (Sieber *et al.*, 2009). Similar to TGFβ, BMPs bind to heteromeric receptor complexes composed of type I and type II (BMPRII, ActRII, ActRIIB) transmembrane serine/threonine kinase receptors and activate the corresponding signalling pathway (Von Bubnoff and Cho, 2001; Miyazono *et al.*, 2005; Xiao *et al.*, 2007; Sieber *et al.*, 2009). In general, BMPs are thought to bind to the type I receptors ALK1/2/3/6 and activate the Smad1/5/8 pathway (Tzavlaki and Moustakas, 2020).

#### Nodal subfamily

Nodal is a multifunctional cytokine that plays a central role in the embryonic stages of mammals and is important in regulating placental development. It does this by suppressing trophoblast proliferation and inhibiting both extravillous trophoblast (EVT) formation and invasion of the decidua (Sarkar *et al.*, 2015). Nodal binds to the type I receptors ALK4/ALK7 and the type II receptors ActRIIA and ActRIIB to induce Smad2/3 activation (Reissmann *et al.*, 2001; Nadeem *et al.*, 2011). The signalling process is modulated by cripto (TDGF1), which acts as a co-receptor for nodal. The regulation of nodal activity by cripto is complex, as cripto can act both as a membrane-bound and a soluble co-receptor, with opposing effects on nodal-induced signal transduction (Reissmann *et al.*, 2001; Yeo and Whitman, 2001; Pauklin and Vallier, 2015).

#### Activin/inhibin subfamily

Activins and inhibins, which belong to the TGFβ subfamily, are versatile proteins with a wide range of physiological functions, including gonadal function, hormonal homeostasis, development, reproduction, and tissue homeostasis (Jones *et al.*, 2006b; Tsuchida *et al.*, 2009; Namwanje and Brown, 2016). Both activins and inhibins are composed of β-subunits, with activins forming dimers of two β-subunits and inhibins forming heterodimers of α- and β-subunits (Pryor-Koishi *et al.*, 2007). Heteromeric complexes of type II receptors (ActRIIA and ActRIIB) and type I receptors (mainly ALK4, but also ALK2 and ALK7) are involved in activin signal transduction (Schneider-Kolsky *et al.*, 2002). Upon binding of activin to the respective type II receptor, the type I receptors are activated and initiate the phosphorylation of intracellular Smad2/3. These newly formed complexes, together with Smad4, are translocated to the nucleus and modulate the expression of specific target genes (Moustakas and Heldin, 2009). Inhibins counteract activin signalling by binding one of the type II receptors to the type III receptor, betaglycan. This interaction binds the II receptors in an inactive complex, inhibiting further signal transduction (Namwanje and Brown, 2016).

#### GDF subfamily

Within the GDF subfamily of TGFβ signalling, a limited number of factors, in particular GDF8 and GDF11 play critical roles in early placental development and function. GDF8 and GDF11, due to their structural similarities (Wu *et al.*, 2022), primarily use the ActRIIA and ActRIIB type II receptors, which are coupled to the ALK4 and ALK5 type I receptors and activate the Smad2/3 signalling pathways. In addition, their signalling is modulated by extracellular antagonists, in particular follistatin-related protein-3 (FSTL3) (Walker *et al.*, 2017). On the other hand, GDF15, also known as macrophage inhibitory cytokine 1 (MIC-1), is a distant member of the family and its mechanism of action seems not to involve TGFβ receptors activation (Mullican *et al.*, 2017; Olsen *et al.*, 2017). The main effects of GDF15 have been related to metabolic regulation through the regulation of PI3K/Akt/ERK kinases (Rochette *et al.*, 2020).

## TGFβ signalling in PE

PE is an inflammatory syndrome, and its incidence is on the rise globally. According to the World Health Organization (WHO), the incidence of PE ranges between 2% and 10% of pregnancies worldwide (Gestational Hypertension and Preeclampsia: ACOG Practice Bulletin, Number 222, 2020). It is a major cause of preterm birth and intrauterine growth restriction (Phipps *et al.*, 2019; Raguema *et al.*, 2020). PE is a complex and heterogeneous condition and the underlying pathophysiology is not yet fully understood (Huppertz, 2008b; Than *et al.*, 2018). The syndrome is classified according to the time of diagnosis, before or after 34-week gestation, and subdivided into early-onset (EO) and late-onset (LO) PE (von Dadelszen *et al.*, 2009; Tranquilli *et al.*, 2013; Wójtowicz *et al.*, 2019). While EOPE and LOPE share a number of common, mainly clinical, characteristics, they have different maternal and foetal outcomes, suggesting different underlying etiologies (Tranquilli *et al.*, 2013; Rana *et al.*, 2019; Parada-Niño *et al.*, 2022a). EOPE is often considered as a more severe form, associated with inadequate early placentation, placental dysfunction, reduced placental volume, intrauterine growth restriction and immune maladaptation, and carries a higher risk of future disease for the mother and offspring (Valensise *et al.*, 2008; Li *et al.*, 2014b, 2022). LOPE, on the other hand, is primarily considered a maternal disorder with milder complications, associated with maternal endothelial dysfunction and is generally associated with a more favourable foetal outcome (Valensise *et al.*, 2008). Despite these differences, the placental-derived molecular mechanisms of EO and LOPE remain unknown, and consequently there is a lack of sufficient molecular criteria to distinguish between the PE subtypes (Valensise *et al.*, 2008; Wu *et al.*, 2019; Stepan *et al.*, 2020). One of the key features associated with PE is impaired trophoblast invasion and endothelial dysfunction, both of which contribute to abnormal placental development (Huppertz, 2008b; Than *et al.*, 2018). Studies have shown that villous trophoblasts of PE placentas develop an immature phenotype, both ultrastructurally and biochemically, compared to normal placentas. In particular, EVT cells in the decidua of PE women have a less invasive and more proliferative phenotype than those from uncomplicated pregnancies (Redline and Patterson, 1995).

Placentation, the process of formation and development of the placenta, is crucial for a successful pregnancy. Early in a healthy pregnancy, EVTs invade deep into the endometrial lining of the uterus and remodel the uterine spiral arteries into highly conductive vessels (Pijnenborg *et al.*, 1991; Vanwijk *et al.*, 2000; Brosens *et al.*, 2011). This remodelling ensures an optimal and high flow of oxygenated blood to the uteroplacental bed, which is critical for placental and foetal development (Pijnenborg *et al.*, 1991; Meekins *et al.*, 1994; Brosens *et al.*, 2011; Lyall *et al.*, 2013). In normal placental development, the spiral arteries undergo significant changes during remodelling, including an increase in terminal luminal diameter and loss of elastic and muscular components. These changes extend into the inner third of the myometrium and result in a loss of vascular smooth muscle condensation near the myometrial decidual junction (Vanwijk *et al.*, 2000; Brosens *et al.*, 2011). However, this process is incomplete in PE. Abnormal placentation in PE is characterized by impaired trophoblast invasion, particularly EVT dysfunction, resulting in shallow invasion and a failure to remodel the spiral arteries (Pijnenborg *et al.*, 1991; Lyall *et al.*, 2013; Lin *et al.*, 2020). Terminal dilatation of the spiral arteries is less extensive and smooth muscle removal is inadequate and does not extend beyond the decidua (Brosens *et al.*, 2011). This failed remodelling of the spiral arteries contributes to placental hypoxia, oxidative stress, and endoplasmic reticulum stress, disrupting normal blood flow patterns and reducing nutrient and oxygen delivery to the placenta (Burton *et al.*, 2009b). The resulting ischaemia and hypoxia of the placenta are important factors in the development of PE. The impaired blood flow and inadequate nutrient supply trigger a cascade of events leading to the induction of the possible pathophysiological features that may later develop into the clinical symptoms of PE (Burton *et al.*, 2009a; Brosens *et al.*, 2011). Inadequate placentation, shallow trophoblast invasion, and impaired immune (Mercnik *et al.*, 2022) and endothelial function (Zhou *et al.*, 2019) in PE emphasize its complexity and the need to understand the underlying mechanisms driving its development.

TGFβ signalling has been implicated in the pathophysiology of PE, but its exact role is still not understood. Several TGFβ ligands and cofactors have been suggested as potential biomarkers in the development of PE. Endoglin (Eng) has received much attention, as elevated levels of soluble Eng (sEng) are found in both the serum and placenta of PE patients. These elevated levels correlate with the severity of the syndrome, and can be detected before the onset of clinical manifestations (Venkatesha *et al.*, 2006; Perucci *et al.*, 2014; Leavey *et al.*, 2016). Different mechanisms have been proposed to the increase in soluble Eng expression in PE. For example, the PE placenta is associated with a deficiency of antioxidants such as heme-oxygenase, superoxide dismutase, and catalase. This deficiency leads to in increased oxidative stress, which in turn increases the production of Eng and secretion of its soluble form in the PE placenta (Gregory *et al.*, 2014). Additionally, oxysterols, agonists of liver X receptors have been shown to induce Eng expression in the placenta affected by PE (Henry-Berger *et al.*, 2008; Margioula-Siarkou *et al.*, 2021). While Eng is best known for its interaction with TGFβ1 and TGFβ3 isoforms, it has the ability to interact with other ligands of the TGFβ superfamily, such as BMPs (Barbara *et al.*, 1999).

The literature on the cellular origin, expression, and function of TGFβ family ligands in PE remains controversial and incomplete (Table 2). Contradictory published data on TGFβ isoform levels in combination with a not always well-defined classification of the PE samples used do not allow a selective role of TGFβ in this pathophysiology. Interestingly in placental tissue, Xu *et al.* (2016) observed a significant upregulation of the Smad2/3 pathway induced by TGFβ in the placenta of EOPE, suggesting a potential role of TGFβ ligands in the pathology of this condition. TGFβ1 is elevated in the plasma and serum of PE patients (Djurovic *et al.*, 1997; Benian *et al.*, 2002; Hennessy *et al.*, 2002; Shaarawy *et al.*, 2016), and has been associated with an increase in diastolic blood pressure (Benian *et al.*, 2002). Consistent with this, higher levels of active TGFβ1 have been observed in both, EO- and LO-PE subtypes of PE, when compared to normotensive controls (Djurovic *et al.*, 1997). However, Hennessy *et al.* (2002) found lower serum TGFβ1 levels in women with PE, possibly due to the differences in sampling methods. Similar to TGFβ1, TGFβ2 is elevated in the serum of patients with PE (Shaarawy *et al.*, 2016). The expression of GDF8 and its antagonist FSTL3 was significantly increased in the maternal serum of PE subjects compared to controls (Pryor-Koishi *et al.*, 2007). The upregulation of FSTL3 and GDF8 was also confirmed in the PE placenta, suggesting that the tissue itself may be a source of circulating GDF8 and FSTL3 in PE (Guo *et al.*, 2012). Of note, inhibin A and activin A, but not inhibin B, are elevated in women with PE (Yair *et al.*, 2001; Mylonas *et al.*, 2006). As activin A levels are also elevated in PE placentas, it is reasonable to speculate that secreted placental activin A contributes, at least in part, to these differences observed in the maternal circulation (Manuelpillai *et al.*, 2001). Using an optimized method for co-expression network analysis, Tejera *et al.*, identified a number of TGFβ superfamily genes that are upregulated in PE placenta. In addition to *ENG*, an increased expression of *INHBA* (encoding inhibin A) and *ACVR1* (encoding the BMP receptor ALK2) was quantified (Tejera *et al.*, 2013). *ACVR1*, as well as *ACVR2A* (encoding ActRIIA), was already found to be upregulated in PE placenta and maternal circulation (Inkeri Lokki *et al.*, 2017; Li *et al.*, 2022). In particular, *ACVR1* mRNA levels in decidua correlate with the severity of PE (Yong *et al.*, 2014). Furthermore, in a study conducted in a Brazilian cohort, *ACVR2A* was associated with the development of EOPE (Ferreira *et al.*, 2015). Further analysis using single-nucleotide polymorphisms identified a common maternal PE susceptibility locus on chromosome 2q22-23, that affects ACVR2A expression and contributes to the development of PE (Roten *et al.*, 2008). Despite recent developments, it is still crucial to further elucidate the role of TGFβ signalling and dissect the interactions within the different branches of the TGFβ pathway to gain a deeper understanding of their contribution to the pathogenesis of PE, and to unravel their potential as targets or biomarkers for this critical condition. Given the proposed different subtypes of PE, it is highly recommended to study the TGFβ signalling pathways in early- and late-onset PE cohorts separately in future (Valensise *et al.*, 2008; Wu *et al.*, 2019). This focused approach may deepen our understanding of the disease, elucidate specific mechanisms, and reveal potential therapeutic targets specific to each subgroup.

**Table 2.** Systemic and tissue levels of TGFβ superfamily ligands in PE.

TGFβ superfamily ligand	Subtype of PE	Type of control	Sample	Changes of ligand levels in PE	Reference
TGFβ1	Mild/Severe EO/LOPE	Normotensive pregnancy (Unmatched for GA)	Plasma	Elevated in all PE subtypes	Perucci *et al.*, 2014
	PE	Normotensive pregnancy (Matched for GA)	Plasma	Elevated	Benian *et al.*, 2002; Peraçoli *et al.*, 2008
	Mild/Severe/PE+ FGR	Normotensive pregnancy (Unmatched for GA)	Plasma	Active TGFβ elevated in all PE subtypes	Djurovic *et al.*, 1997
	PE	Normotensive pregnancy (Matched for GA)	Serum	No difference in total or active TGFβ1	Huber *et al.*, 2002
	PE	Normotensive pregnancy (Unmatched for GA)	Serum	No difference in total or active TGFβ1	Hennessy *et al.*, 2002
	Mild/Severe PE	Normotensive pregnancy (Matched for GA)	Serum	Decreased levels in mild PE, elevated in severe PE	Xu *et al.*, 2017
	PE	Normotensive pregnancy (Matched for GA)	Placenta	Elevated	Benian *et al.*, 2002
	PE	Normotensive pregnancy (Unmatched for GA)	Placenta	Elevated	Zhang *et al.*, 2019
	PE	Preterm normotensive pregnancy	Placenta	Elevated	Yi *et al.*, 2021
	PE	Normotensive pregnancy (Unmatched for GA)	Placenta	No difference	Hennessy *et al.*, 2002
TGFβ2	Mild/Severe PE/Eclampsia	Normotensive pregnancy (Matched for GA)	Serum	Elevated in Severe PE and Eclampsia	Shaarawy *et al.*, 2016
TGFβ3	PE	Normotensive pregnancy (Matched for GA)	Placenta	Elevated	Caniggia *et al.*, 1999
	EOPE	Preterm normotensive pregnancy	Placenta	Reduced	Yi *et al.*, 2021
BMP2	EOPE	Preterm normotensive pregnancy	Placenta	Reduced	Yi *et al.*, 2021
BMP4	PE	Normotensive pregnancy (Unmatched for GA)	Serum	No difference	Gallardo-Vara *et al.*, 2020
	EOPE	Preterm normotensive pregnancy	Placenta	Reduced	Yi *et al.*, 2021
BMP5	EOPE	Preterm normotensive pregnancy	Placenta	Reduced	Yi *et al.*, 2021
Nodal	PE	Preterm normotensive pregnancy (Matched for GA)	Placenta	Elevated	Nadeem *et al.*, 2011
Activin A	Mild/Severe PE/Gestational hypertension	Normotensive pregnancy (Matched for GA)	Serum	Elevated in severe PE > mild PE and gestational hypertension	Xu *et al.*, 2017
	PE	Normotensive pregnancy (Matched for GA)	Serum	Elevated	Yair *et al.*, 2001
	PE	Normotensive pregnancy (Unmatched for GA)	Serum	Elevated	Manuelpillai *et al.*, 2001
	PE	Normotensive pregnancy (Unmatched for GA)	Placenta	Elevated	Manuelpillai *et al.*, 2001
Inhibin A	Mild/Severe PE/Gestational hypertension	Normotensive pregnancy (Matched for GA)	Serum	Elevated in severe PE > mild PE and gestational hypertension	Xu *et al.*, 2017
	PE	Normotensive pregnancy (Matched for GA)	Serum	Elevated	Yair *et al.*, 2001
	EOPE	Preterm normotensive pregnancy	Placenta	Elevated	Yi *et al.*, 2021
Inhibin B	PE	Normotensive pregnancy (Matched for GA)	Serum	No difference	Yair *et al.*, 2001
	EOPE	Preterm normotensive pregnancy	Placenta	Elevated	Yi *et al.*, 2021
Myostatin (GDF8)	Mild/Severe PE	Normotensive pregnancy (Matched for GA)	Serum	Elevated	Guo *et al.*, 2012
	Mild/Severe PE	Normotensive pregnancy (Matched for GA)	Placenta	Elevated	Guo *et al.*, 2012
Endoglin	Mild/Severe PE	Normotensive pregnancy (Unmatched for GA)	Plasma	Increasing with the severity of PE	Perucci *et al.*, 2014
	Mild/Severe PE	Normotensive pregnancy (Matched for GA)	Serum	Increasing with the severity of PE	Venkatesha *et al.*, 2006; Xu *et al.*, 2017; Zhang *et al.*, 2020
	Mild/Severe PE	Normotensive pregnancy (Unmatched for GA)	Serum	Increasing with the severity of PE	Leaños-Miranda *et al.*, 2019; Gallardo-Vara *et al.*, 2020
	Mild/Severe PE	Normotensive pregnancy (Matched for GA)	Placenta	Increasing with the severity of PE	Venkatesha *et al.*, 2006
Follistatin related gene protein	PE	Normotensive pregnancy (Matched for GA)	Serum	Elevated	Pryor-Koishi *et al.*, 2007
	PE	Normotensive pregnancy (Matched for GA)	Placenta	Elevated	Pryor-Koishi *et al.*, 2007
FSTL3	Mild/Severe PE	Normotensive pregnancy (Matched for GA)	Serum	Elevated	Guo *et al.*, 2012
	Mild/Severe PE	Normotensive pregnancy (Matched for GA)	Placenta	Elevated	Guo *et al.*, 2012

EOPE, early onset PE; LOPE, late onset PE; FGR, fetal growth restriction; GA, gestational age.

## The TGFβ superfamily and placental cells: from early weeks until pregnancy term

### Trophoblast cells

**Figure 2.**
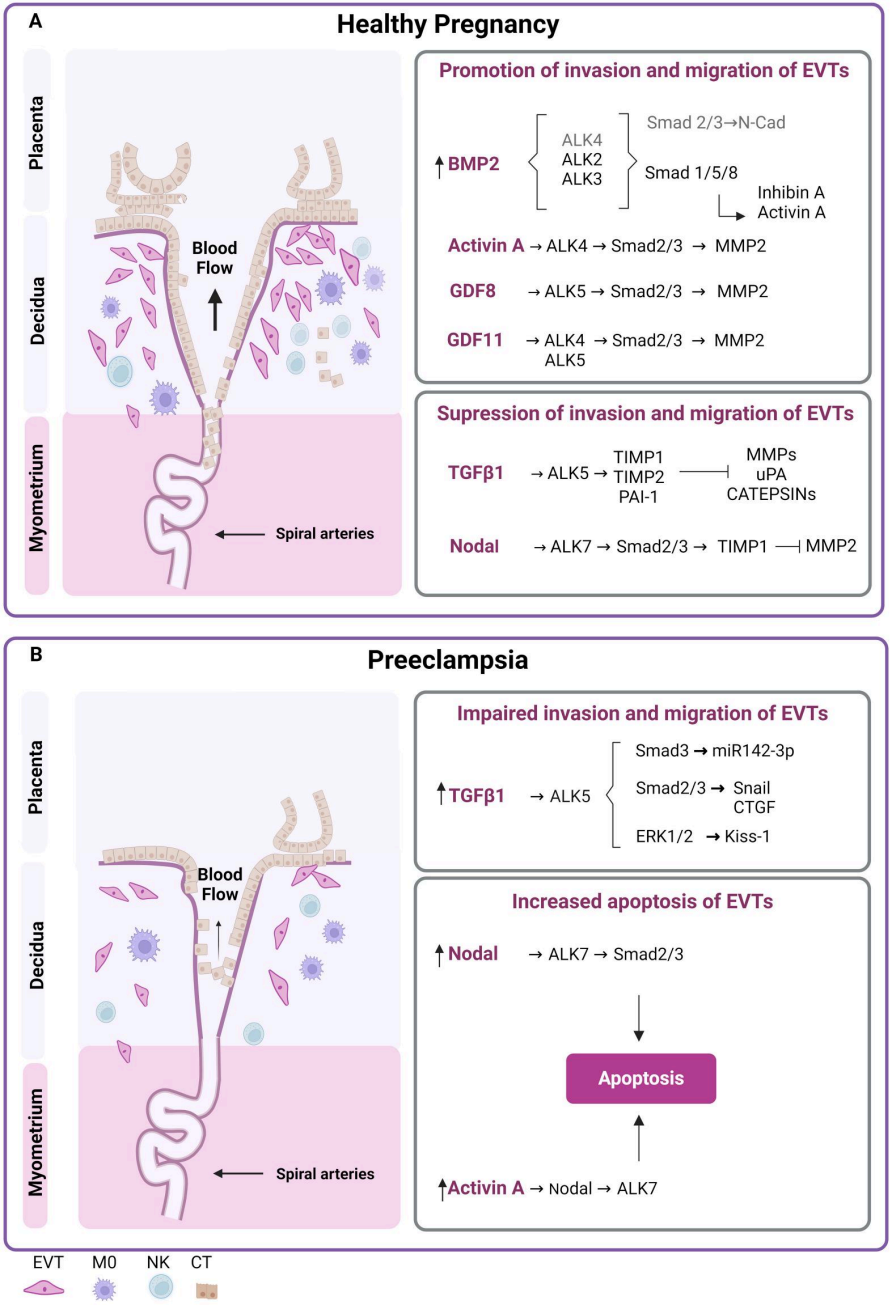
**Summary of the TGFβ family induced signalling in EVT.** During placentation, the coordinated migration and invasion of EVTs is critical for the maintenance of a successful pregnancy. During the first trimester, CTs undergo an EMT-like process and differentiate into EVTs. These EVTs migrate and invade the decidua and maternal spiral arteries in the myometrium. Highly regulated interactions between EVTs and surrounding cells, including NK cells, macrophages (M0), and stromal cells, play an important role in the immunological acceptance and depth of trophoblast invasion. In a normal pregnancy (**A**), TGFβ signalling regulates the process of trophoblast invasion. Different ligands, such as BMPs, activins, and GDFs, are involved in promoting EVT invasion and migration. For example, BMP2 can directly or indirectly activate different receptors (ALK2, ALK3, or ALK4), subsequently inducing Smad1/5/8 or Smad2/3 activation, depending on the context of signalling and receptor binding. Activin A binds to ALK4 and activates Smad2/3, leading to the activation of metalloproteinase 2 (MMP2). GDF8 and GDF11 also activate Smad2/3 and downstream MMP2. To maintain a balance in trophoblast invasion, the secretion of inhibitors or molecules that attenuate their activity regulates the activity of trophoblast proteases. TGFβ and Nodal subfamilies are involved in the induction of TIMPs, with TGFΒs signalling through ALK5 and Nodals signalling through ALK7. In PE (**B**), trophoblasts fail to invade the spiral arteries adequately, resulting in reduced blood flow and impaired utero-placental perfusion. The impaired invasion appears to be the first change on cellular level that contributes to the pathogenesis of PE. TGFβ1 inhibits cell invasion through ALK5 and Smad2/3 induction, and contributes to the upregulation of miR142-3p and Snail expression in PE. Non-canonical ERK1/2 signalling may also be involved in inhibiting invasion by inducing Kiss-1 expression. Interestingly, elevated levels of Activin A in PE trigger apoptosis via the Nodal/ALK7 pathway rather than by inducing MMP expression. Elevated levels of Nodal, on the other hand, do not inhibit EVT migration but rather induce apoptosis via the ALK7 pathway. M0, macrophages.

Considering the complex role of the placenta in foetal–maternal communication, it may not be surprising that its diverse functions at different stages of normal and abnormal pregnancies involve the coordinated actions of several cell types. Within the first weeks of pregnancy, the human placenta generates epithelial trophoblasts with diverse biological roles including in the attachment of the conceptus to the uterine wall, the establishment of early histotrophic response, and the adaption of the maternal uterine vasculature. Different types of trophoblasts, including stem cells, progenitors, and differentiated subtypes with multiple functions develop (Knöfler *et al.*, 2019). Cytotrophoblasts (CTs), progenitor cells located in the first trimester placental villi, fuse to form the multinucleated syncytium, which plays a key role in nutrient transport and hormone production (Gauster *et al.*, 2022). Thereafter CTs undergo a differentiation process leading to the formation of EVTs through a process similar to that which is well documented in cancer biology, known as the epithelial-to-mesenchymal transition (EMT) (Gonzalez and Medici, 2014; Davies *et al.*, 2016). During the EMT-like differentiation, EVTs partially lose their well-organized epithelial phenotype and transition to a migratory and invasive mesenchymal phenotype. Key features of this differentiation include changes in cell polarity and adhesion (Kokkinos *et al.*, 2010). Essentially, trophoblasts acquire mesenchymal characteristics by exhibiting migratory and invasive capabilities, upregulation of EMT transcription factors such as Snail, increased Wnt signalling, and downregulation of epithelial polarity genes such as Occludin and ZO1 (Knöfler and Pollheimer, 2013). It is important to emphasize that trophoblasts simultaneously maintain certain epithelial features, characterized by the expression of cytokeratin 7 and the absence of mesenchymal vimentin induction (Davies *et al.*, 2016; Haider *et al.*, 2017). These changes are critical for successful embryo implantation in the early weeks of pregnancy (Davies *et al.*, 2016; Haider *et al.*, 2017). These processes involve the migration of EVT cells into the maternal decidua and the remodelling of the maternal spiral arteries to ensure an adequate blood supply to the developing placenta (Haider *et al.*, 2022). Dysregulation of trophoblast proliferation and EVT invasion may contribute to conditions like PE, placenta accreta, and recurrent miscarriage (Incebiyik *et al.*, 2014). Among the signalling molecules involved in regulating trophoblast function, members of the TGFβ superfamily play significant and distinct roles during the first trimester of pregnancy (Fig. 2). Research on the TGFβ superfamily and trophoblast function at pregnancy term is relatively limited, and further investigation is necessary to gain a comprehensive understanding of the pregnancy progression and its potential implications for maternal and foetal health.

#### TGFβ signalling in trophoblasts

The balance between factors that promote or inhibit cell invasion is essential for a normal placentation. The TGFβ ligands TGFβ1, TGB2, and TGFβ3, exert several biological effects in trophoblasts and in the regulation of placental invasion (Jones *et al.*, 2006b). As mentioned above, TGFβs bind to the type I receptor ALK5 and activate the Smad2/3 signalling cascade (Budi *et al.*, 2017). The expression of some TGFβ signalling components, Smad2/3/4, TGFβRI, TGFβRII, TGFβ1, and TGFβ2, is highest in trophoblasts during the first trimester and decreases during pregnancy (Xuan *et al.*, 2007). All three forms of TGFβ contribute, at least in part, through different mechanisms to the inhibition of trophoblast invasion and migration (Irving and Lala, 1995; Caniggia *et al.*, 1999; Karmakar and Das, 2002; Tse *et al.*, 2002; Jones *et al.*, 2006b). Consistently, TGFβ3 expression decreases around the ninth week of gestation, correlating with a switch from trophoblast invasion and migration to a proliferative state, characterized by increased fibronectin synthesis (Caniggia *et al.*, 1999). For successful invasion, trophoblasts secrete proteases such as matrix metalloproteases (MMPs), urokinase-type plasminogen activators (uPA), and cysteine proteases such as cathepsins (Lash *et al.*, 2005; Li *et al.*, 2021). The activity of trophoblast proteases is tightly regulated by natural inhibitors (Librach *et al.*, 1994; Karmakar and Das, 2002), such as plasminogen activator inhibitor 1 (PAI-1) and tissue inhibitors of metalloproteinases (TIMPs). TGFβ1, TGFβ2, and TGFβ3 induce the activation of PAI-1 and TIMPs (Lash *et al.*, 2005), thereby increasing cell adhesion to the extracellular matrix (ECM) (Irving and Lala, 1995). Local inflammatory mediators, such as IL1β and TGFβ1, are balanced to further regulate the activity of these enzymes. In this sense, IL-1β promotes the expression of uPA and MMP9, thereby promoting invasion, whereas TGFβ1 upregulates TIMP-1, TIMP-2, and PAI-1, which in contrast inhibit the action of uPA and MMP9 (Laiho *et al.*, 1987; Graham, 1997). Additionally, IL1β induces the expression of MT-MMP1 or MMP14, a collagenase involved in ECM degradation, but exposure to TGFβ1 abolishes the expression of MT-MMP in JEG-3 cells (Librach *et al.*, 1994; Karmakar and Das, 2002).

Another important milestone towards a successful pregnancy is the proper invasion of the EVT. Using RNAseq analysis, Haider *et al.* (2022) identified that TGFβ/Smad2/3 signalling governs the differentiation of placental EVTs, as demonstrated in primary EVTs and trophoblast organoids. The same group characterized different Smad2/3 phosphorylated residues in trophoblast subtypes. Thus, while C-terminal Smad2/3 phosphorylation was proposed to regulate EVT function and differentiation, Smads2 linker phosphorylation was mainly associated with proliferative primary CTs (Haider *et al.*, 2017). In immortalized HTR8/SVneo cells, cell invasion required the expression of the cell membrane junction protein VE-cadherin. Notably, incubation with TGF-β1 induced the activation of the Smad2/3 signalling and the expression of the Snail and Slug transcription factors, and genetic inhibition of Snail was sufficient to prevent invasion of primary trophoblasts and the HTR8/SVneo cell line (Cheng *et al.*, 2013). Conversely, a recent study showed how TGFβ1 can activate the non-canonical ALK5-ERK1/2 pathway and inhibit invasion in primary EVTs and HTR8/SVneo cells, by inducing downstream kisspeptins (Fang *et al.*, 2022). Interestingly, elevated levels of kisspeptin-1 have been observed in PE placenta, suggesting their potential contribution to disrupted PE pathology (Kapustin *et al.*, 2020). In addition, miR142-3p, which is highly upregulated in PE placenta, has been shown to induce trophoblast invasion by activating the TGFβ1/Smad3 signalling pathway in the HTR8/SVneo cell line (Liu *et al.*, 2019). As mentioned above, in some cell types the TGFβ co-receptor Eng is able to balance Smad signalling between ALK1-Smad1/5/8 and ALK5-Smad2/3. In response to TGFβ1 and/or TGFβ3, Eng was shown to be essential to prevent EVT invasion, and Eng inhibition triggered EVT outgrowth, migration, and fibronectin production in villous explants (Caniggia *et al.*, 1997b). Indeed, TGFβ3 triggers the premature invasive phenotype of the trophoblast, and its downregulation rescues the abnormal invasive phenotype observed in PE placentas (Caniggia *et al.*, 1999; Cowden Dahl *et al.*, 2005). Furthermore, a critical link between TGFβ3 and hypoxia is emerging in normal placental development, where elevated oxygen levels at 10–12 weeks of gestation downregulate HIF-1α and TGFβ3 thereby regulating trophoblast invasiveness (Tal, 2012). However, as demonstrated in JAR, JEG-3, and villous explants in PE, unsuccessful suppression of HIF-1α increases placental TGFβ3 expression, leading to the aberrant trophoblast invasion and persistent placental hypoxia, causing a detrimental loop where chronic/continuous hypoxia further stimulates HIF-1α and TGFβ3 expression (Caniggia *et al.*, 1999; Schäffer *et al.*, 2003; Nishi *et al.*, 2004; Xu *et al.*, 2019).

ECM remodelling is a critical aspect of a successful pregnancy, intricately regulating trophoblast invasion and placental development (Huppertz, 2008b). Furthermore, the composition of ECM determines its effect on trophoblast invasion (Huppertz, 2008a). A study by Xu *et al.* (2019) investigated lysyl oxidase (LOX) and LOX-like protein 2 (LOX2L) signalling components involved in ECM remodelling and HTR8/SVneo trophoblast invasion in PE placentas. The TGFβ1/Smad3 pathway has been shown to inhibit LOX and LOXL2-dependent induction of collagen production, thereby suppressing the migration and invasion of trophoblasts. Downregulation of LOX and LOXL2 has also been observed in PE placenta (Xu *et al.*, 2019). Additionally, connective tissue growth factor (CTGF), also known as CNN2, is an important downstream target of TGFβ-Smad2/3 signalling and plays a role in extracellular matrix deposition. Elevated levels of CTGF have been detected in the placenta and serum of patients with severe PE and foetal growth restriction, suggesting its potential involvement in regulating of trophoblast invasion in PE. Importantly, genetic knockdown of CTGF partially prevented TGFβ1-induced inhibition of invasion of primary trophoblasts and the HTR8/SVneo cell line (Oh *et al.*, 2009; Chen *et al.*, 2012; Cheng *et al.*, 2017).

In summary, studies have implicated various components of the TGFβ signalling pathway, including Smad2/3, TGFβ receptors, and downstream effectors such as VE-cadherin and kisspeptins, in trophoblast differentiation and invasion. Dysregulation of TGFβ signalling, as seen in PE, may contribute to impaired trophoblast invasion and placental dysfunction.

#### BMP signalling in trophoblasts

The BMP subfamily is one of the most important signalling pathways in developmental processes, as it is involved in trophoblast differentiation and invasion, cell growth, and apoptosis (Jones *et al.*, 2006b). BMP2 and BMP4 play important roles in placental development and particularly in trophoblast invasion. BMP2 is localized to all trophoblast subtypes and to the decidua during the first trimester of pregnancy, with its levels increasing during this period (Yi *et al.*, 2021). *In vitro* models such as the use of primary trophoblasts and the cell line HTR8/SVneo have demonstrated the action of BMP2 in regulating human trophoblast invasion and spiral artery remodelling (Zhao *et al.*, 2018b, 2020a,b). During normal pregnancy, there are increased serum levels of BMP2, some of which is secreted by trophoblasts (You *et al.*, 2021). Of note, a recent study identified Hofbauer cells (HBCs) as an additional source of BMP2. Crosstalk between HBCs and trophoblast leads to upregulation of BMP6 via ALK3-Smad2/3-Smad4 signalling in primary trophoblasts, promoting their invasion and vascular mimicry (Deng *et al.*, 2023). In trophoblasts, BMP2 has been shown to regulate EMT-like process, cell adhesion, and invasion (Luo, 2017). To promote trophoblast differentiation, BMP2 regulates the expression of adhesion molecules and genes involved in ECM remodelling in primary trophoblast and HTR8/SVneo (Yi *et al.*, 2021). BMP2 upregulates the expression of ECM-associated genes, including *COL6A1, COL7A1, ITGA2, ITGA6, ADAM12*, and *MMP11* (Yi *et al.*, 2021). The expression of the EMT transcription factor Snail (Luo, 2017) is induced by BMP2 through the induction of lncRNA NR026833.1, which binds to miR502-5p and upregulates Snail expression in primary trophoblasts (You *et al.*, 2021). Snail, in turn, induces *MMP2*, a key molecule involved in trophoblast invasion at 6–8 weeks of gestation (You *et al.*, 2021). In primary trophoblasts and in the HTR8/SVneo cell line, BMP2 also promotes invasion and endothelial-like tube formation through an *ID1*–IGFBP3–Slug axis (Zhao *et al.*, 2020b).

The mechanisms by which BMP2 regulates trophoblast invasion are complex. BMP2 binding activates non-canonical Smad2/3 signalling leading to the induction of N-cadherin in primary EVTs and HTR8/SVneo cells (Zhao *et al.*, 2018b). Pharmacological inhibition of ALK2/3, but not ALK4/5/7, effectively diminished the induction of Smad2/3 phosphorylation in response to BMP2 (Zhao *et al.*, 2018a,b). Interestingly, ALK3 is required for BMP2 to induce inhibin A and activin A production in primary EVTs, which may act in an autocrine manner (Zhao *et al.*, 2018a). Other factors, such as the adhesion molecule AMIGO2 and the membrane-bound BMP and activin inhibitor (BAMBI), are involved in BMP2-induced trophoblast invasion and WNT/β-catenin signalling crosstalk in primary trophoblast and HTR8/SVneo (Zhao *et al.*, 2020a; Yi *et al.*, 2021). In EOPE placenta, particularly in trophoblasts, BMP2 levels are decreased, as are the ligands BMP4 and BMP5. In contrast, the expression of the BMP type II receptor BMPR2 is increased (Yi *et al.*, 2021), perhaps as a compensatory mechanism. Consistent with the downregulation of *BMP2* mRNA levels in EOPE trophoblasts (Yi *et al.*, 2021), the expression of lncRNA NR026833.1 is downregulated in EOPE (You *et al.*, 2021). The downregulation of *BMP2* mRNA and lncRNA NR026833.1 expression in EOPE suggests potential mechanisms contributing to trophoblast dysfunction in PE.

BMP4 is used to stimulate the differentiation of various human pluripotent stem cells (hPSCs) origins, including embryonic stem cells (ESCs) and induced pluripotent stem cells (iPSCs), into different trophoblast cell models (Kojima *et al.*, 2017; Jang *et al.*, 2022; Soncin *et al.*, 2022). To introduce BMP4 to hPSCs induces the formation of self-renewing trophoblast stem cells (TSCs), which possess the capability to undergo further differentiation into EVTs and ST (Jang *et al.*, 2022; Soncin *et al.*, 2022). Notably, Kojima *et al.* (2017) have demonstrated that the concentration-dependent stimulation of BMP4 in human-induced pluripotent stem cells (hiPSCs) leads to the direct differentiation of cells into EVTs and STs without the self-renewing TSC stage. Due to the challenges of obtaining human first trimester tissue samples and the potential biological differences between species, differentiated hPSCs have been used as an alternative model to study trophoblast biology. Successful differentiation of hPSCs into trophoblast-like stem cells requires the inhibition of activin/nodal signalling by BMP4 stimulation (Xu *et al.*, 2002). Furthermore, inhibition of activin/nodal signalling via BMP4 activates FGR signalling, leading to hPSC differentiation into the β-human chorionic gonadotropin (βhCG) hormone-secreting multinucleated ST (Sudheer *et al.*, 2012). In particular, BMP4 activates amniotic and/or mesodermal markers under certain conditions. Therefore, its utilization as a differentiation agent remains controversial, and different models and protocols have been proposed (Roberts *et al.*, 2018; Knöfler *et al.*, 2019; Io *et al.*, 2021).

In summary, the BMP subfamily, particularly BMP2 and BMP4, plays a critical role in placental development, trophoblast invasion and differentiation. The understanding of BMP signalling in PE trophoblasts remains poor, and further studies are needed to clarify the role of BMPs in trophoblast function.

#### Nodal signalling in trophoblasts

Nodal is a multifunctional cytokine, of key importance during mammalian embryonic development, and involved in the regulation of placental development by inhibiting trophoblast proliferation, EVT formation, and EVT invasion of the decidua (Sarkar *et al.*, 2015). During early pregnancy, nodal and ALK7 are expressed in villous and EVT cells, and their levels are strongly upregulated in PE placentas (Nadeem *et al.*, 2011). In a healthy pregnancy, high levels of nodal/ALK7 inhibit trophoblast proliferation and invasion, and may contribute to syncytialization. Subsequently, reduced levels of nodal/ALK7 allow trophoblast proliferation and invasion. Mechanistically, in HTR8/SVneo and villous explants, Nodal/ALK7 activates the Smad2/3 signalling cascade and upregulates TIMP-1, which subsequently inhibits MMP2 and MMP9 (Nadeem *et al.*, 2011).

Nodal levels are regulated by molecules involved in trophoblast proliferation, such as Lefty (Li *et al.*, 2018), miR-378a-5p (Parada-Niño *et al.*, 2022), miR-376 (Fu *et al.*, 2013), and miR-454 (Adu-Gyamfi *et al.*, 2020). miR-376c inhibits both ALK5 and ALK7 and consequently impairs TGFβ/nodal signalling, leading to increased cell proliferation and invasion. Interestingly, miR376c levels are reduced in placentas and plasma of women with PE (Fu *et al.*, 2013). In PE, where nodal/ALK7 is overexpressed, the activation of Smad2/3 signalling leads to impaired trophoblast invasion and increased apoptosis as shown in HTR8/SVneo and villous explants (Nadeem *et al.*, 2011). The inhibition of JEG-3, JAR, and Bewo proliferation by Nodal is partly mediated by the p27-cyclin E/Cdk2 pathway, resulting in G1-cell cycle arrest (Munir *et al.*, 2004). TGFβ1 can inhibit activin/nodal-induced EVT formation shown in TSC model (Sarkar *et al.*, 2015), while activin A enhances nodal signalling (Yu *et al.*, 2012). Maternal Nodal can induce foeto-placental nodal signalling and modulate the PE susceptibility gene *STOX1* through activin A secretion at the maternal interface (Thulluru *et al.*, 2013). As both activin A and nodal have been shown to be elevated in PE, it has been suggested that their interaction promotes trophoblast apoptosis through activation of the nodal/ALK7 signalling in primary trophoblasts and HTR8/SVneo cell line (Yu *et al.*, 2012).

To sum up, nodal plays a critical role in placental development by regulating trophoblast proliferation, invasion, and apoptosis.

#### Activin and inhibin signalling pathways in trophoblasts

Activins and inhibins are members of the TGFβ subfamily and have a similar structure (Pryor-Koishi *et al.*, 2007). Activin A is the predominant form of activins in pregnancy and is expressed in the syncytiotrophoblast (ST) and underlying CT layers throughout pregnancy. Activin A and inhibin A are secreted by the placenta, decidua, and foetal membranes and are found in the maternal circulation, amniotic fluid, and umbilical cord blood (Florio *et al.*, 2004). Its type I receptors, ALK4 and ALK7, are expressed in the ST during the first and second trimesters and predominantly in the ECs of the villi during the third trimester (Schneider-Kolsky *et al.*, 2002).

Activin A is highly involved in the placentation, acting in an autocrine or paracrine manner, being expressed in the newly formed ST and/or playing a role in the aggregation and syncytialization of CT cells. In the first trimester, activin A stimulates primary trophoblast differentiation towards an invasive phenotype (Bearfield *et al.*, 2005). Villous explant studies have demonstrated that activin A promotes CT outgrowth and fusion, EVT formation, and EVT invasion into the decidua (Caniggia *et al.*, 1997a). To facilitate the foeto-maternal interface, CT differentiate towards the invasive and migratory EVT phenotype in a complex and regulated EMT-like process (Kokkinos *et al.*, 2010). Molecules of the TGFβ superfamily, including activin A, are recognized as potent inducers of the EMT-like process (Li *et al.*, 2014c; Tzavlaki and Moustakas, 2020). Indeed, BMP2 can induce the production, of activin A, leading to the upregulation of N-cadherin, an important marker of EMT (Zhao *et al.*, 2018a,b). Activin A mediated invasion of primary EVTs and HTR8/SVneo is linked to the ALK4-Smad2/3 signalling pathway, leading to the activation of either Snail or Slug and their downstream molecules MMP2, MMP9, and MMP26 (Li *et al.*, 2015a; Adu-Gyamfi *et al.*, 2020; Zhu *et al.*, 2021). Interestingly, excessive levels of activin A can induce apoptosis via the nodal-ALK7 pathway rather than promoting MMP expression (Adu-Gyamfi *et al.*, 2020).

Notably, as shown in primary EVTs and HTR8/SVneo during trophoblast invasion, both TGFβ1 and activin A are capable of signalling through ALK5 and ALK4 receptors, respectively, thereby inducing the Snail transcription factor. Of note, TGFβ has been shown to activate Smad-independent signalling pathways, including MAPK (ERK, p38, and JNK), PI3K, and Rho-like GTPases, whereas activin A preferentially signals via Smad dependent pathways (Matsuzaki, 2011; Li *et al.*, 2015a; Zhu *et al.*, 2021).

Inhibin A, produced by the CT, acts as a feedback mechanism in EVT invasion by antagonizing the production of activin A-activated metalloproteinases (MMP2, MMP7, and MMP9) (Jones *et al.*, 2006a; Adu-Gyamfi *et al.*, 2020). Follistatin, produced by the developing placenta, is an activin A soluble antagonist that regulates EVT outgrowth in villous explants (Caniggia *et al.*, 1997a; Petraglia *et al.*, 1994). Another regulator of activin A is follistatin-related protein (FLRG), which is primarily expressed by the decidua and placental ECs (Ciarmela *et al.*, 2003). FLRG levels are increased in the ST of PE placentas and are associated with low birth weight (Pryor-Koishi *et al.*, 2007). Although follistatin and FLRG give similar signals, their expression differs during pregnancy. While follistatin expression decreases throughout the pregnancy, FLRG expression continues to increase until delivery (Wang *et al.*, 2003; Pryor-Koishi *et al.*, 2007). In PE, placental levels of both activin A and inhibin A are elevated (Yi *et al.*, 2021). Interestingly, hypoxia can enhance the activity of both Inhibin A and Activin A, but their expression is not influenced under low oxygen levels in placental cells (Manuelpillai *et al.*, 2003), suggesting that triggers other than hypoxic conditions may modulate the expression of activin and inhibin ligands during PE.

Both TGFβ1 and activin A are involved in the regulation of βhCG production by primary trophoblasts, although their effects may vary depending on the signalling pathways that they induce and the stage of pregnancy. TGFβ1 has an inhibitory effect, whereas activin A stimulates the expression and secretion of βhCG (Song *et al.*, 1996). The effects of activin A are tightly controlled by the antagonistic effects of inhibin A (Petraglia *et al.*, 1989).

#### GDF signalling cascade

Among the GDF subfamily of the TGFβ signalling, only a few factors have been identified to play a role in the development and function of the early placenta, including GDF8, GDF11, and GDF15. GDF8, also known as myostatin, and its receptor ACVR2B are localized in EVTs in the first and third trimester placenta and contribute to the regulation of normal placentation, by inducing proliferation and migration of primary EVTs and HTR8/SVneo (Peiris *et al.*, 2014). Interestingly, GDF8 can induce the expression of FSTL3, an antagonist of GDF8 signalling, to further enhance invasion in primary EVTs and the HTR8/SVneo cell line via the ALK5-Smad2/3 pathway (Xie *et al.*, 2020). Additionally, the activated ALK5-Smad2/3 pathway induces the expression of *MMP2* in HTR8/SVneo cell line (Fang *et al.*, 2021), thereby stimulating EVTs invasion, providing insight into early placental development, as MMP2 is primarily active between 6–8 weeks of gestation.

GDF11, which is structurally similar to GDF8, induces cell invasion by using the ALK4 and ALK5 type I receptors and subsequently activates Smad2/3 and the expression of *MMP2* in primary EVTs and in HTR8/SVneo cells (Wu *et al.*, 2022). In addition, GDF11 regulates the transcriptional regulator ID2, which is required for MMP2 expression and invasion of EVTs (Wu *et al.*, 2022).

Another member of the GDF subfamily, GDF15, also known as MIC-1, is abundantly expressed in the placenta, and has been localized to the CT and ST in particular (Sugulle *et al.*, 2009). Using the HTR-8/Neo cell line and villous explants, fragile X-related protein 1 (FXR1) was been shown to regulate GDF15 expression (Hong *et al.*, 2022). The maturation and processing of GDF15 are mediated by the activity of MMP26, which is co-localized on villous and EVT cells. GDF15 has been shown to promote EVT apoptosis and inhibit proliferation in the HTR8/SVneo cell line (Li *et al.*, 2014a). However, the mechanisms by which GDF15 modulates TGFβ signalling responses in the placenta remain unclear.

In summary, GDFs are mainly involved in the regulation of EVT invasion. Indeed, the exact mechanisms and the significance in healthy and pathological pregnancies are still unknown and require further investigation.

#### Trophoblast models to study TGFβ signalling

To comprehensively study TGFβ superfamily signalling in trophoblasts, several placental *in vitro* and *ex vivo* models are used, each with unique strengths and limitations (Table 3). These models, including primary trophoblasts, cell lines, placental explants, TSCs, organoids, placenta-on-chip, and animal models, provide valuable insights into trophoblast biology (reviewed in Horii *et al.*, 2020; Sheridan *et al.*, 2020; Haider and Beristain, 2023; Li *et al.*, 2023b; Liu *et al.*, 2023). The choice of the appropriate cell model is crucial, depending on the experimental design and specific research questions related to trophoblast development, differentiation, and function (Table 4). Despite the challenges in achieving universally reproducible models, understanding these models is essential for interpreting TGFβ-induced placental disorders. Each model has strengths and limitations that must be carefully considered when interpreting the TGFβ signalling dynamics. Given the ethical constraints of studying first trimester placental tissue, continued progress in placental research is essential. In addition, the development of new 2D and 3D models is needed to better mimic the multicellular environment as *in vivo* and improve our understanding of TGFβ signalling in trophoblasts.

**Table 3.** Summary of published models to study TGFβ signalling in trophoblasts.

TGFβ superfamily	Ligand	Model system								Studied trophoblast function	Reference
TGFβ	TGFβ1	Primary trophoblast								Differentiation of placental EVTs into intestitial EVTs	Haider *et al.*, 2022
										TGFβ targets expression	Haider *et al.*, 2017
										Invasion	Oh *et al.*, 2009; Chen *et al.*, 2012; Cheng *et al.*, 2013, 2017; Fang *et al.*, 2022
		Placental explants								Invasion	Caniggia *et al.*, 1997b
		Cell lines	JEG-3							Invasion	Karmakar & Das, 2002
			HTR8/SVneo							Invasion	Oh *et al.*, 2009; Chen *et al.*, 2012; Cheng *et al.*, 2013, 2017; Liu *et al.*, 2019; Xu *et al.*, 2019; Fang *et al.*, 2022
		Organoids								Differentiation of placental EVTs into intestitial EVTs	Haider *et al.*, 2022
	TGFβ3	Cell lines		JAR						Invasion	Nishi *et al.*, 2004
				JEG-3						Invasion	Nishi *et al.*, 2004
		Placental explants								Invasion	Caniggia *et al.*, 1997b, 1999
BMP	BMP2	Primary trophoblasts								Invasion	Zhao *et al.*, 2018a,b, 2020a,b; You *et al.*, 2021; Deng *et al.*, 2023
										Differentiation	Yi *et al.*, 2021; You *et al.*, 2021
		Cell lines		HTR8/SVneo						Invasion	Zhao *et al.*, 2018b, 2020a,b
										Endothelial-like tube formation	Zhao *et al.*, 2020b
	BMP4	Trophoblast stem cells (TSC)								Derivation of TSCs from pluripotent stem cells	Sudheer *et al.*, 2012; Kojima *et al.*, 2017; Jang *et al.*, 2022; Soncin *et al.*, 2022
Nodal	Nodal	Primary trophoblasts								Apoptosis	Yu *et al.*, 2012
		Placental explants								Invasion	Nadeem *et al.*, 2011; Fu *et al.*, 2013
										Proliferation	Fu *et al.*, 2013
		Cell lines			JAR					Proliferation	Munir *et al.*, 2004
					JEG-3					Proliferation	Munir *et al.*, 2004
					Bewo					Proliferation	Munir *et al.*, 2004
					HTR8/SVneo					Invasion	Nadeem *et al.*, 2011; Fu *et al.*, 2013
										Proliferation	Fu *et al.*, 2013; Li *et al.*, 2018
										Apoptosis	Yu *et al.*, 2012
		Trophoblast stem cells								EVT formation	Sarkar *et al.*, 2015
Activin/Inhibin	Activin A	Primary trophoblasts								Differentiation	Caniggia *et al.*, 1997a; Bearfield *et al.*, 2005
										Invasion	Li *et al.*, 2015a; Adu-Gyamfi *et al.*, 2020; Zhu *et al.*, 2021
										Apoptosis	Yu *et al.*, 2012
										Hormone production	Song *et al.*, 1996
		Placental explants								Differentiation	Caniggia *et al.*, 1997a
										EVT formation	Caniggia *et al.*, 1997a
										Invasion	Caniggia *et al.*, 1997a
		Cell lines				HTR8/SVneo				Invasion	Li *et al.*, 2015a; Adu-Gyamfi *et al.*, 2020; Zhu *et al.*, 2021
										Apoptosis	Yu *et al.*, 2012
	Inhibin A	Placental explants								EVT growth	Caniggia *et al.*, 1997a; Petraglia *et al.*, 1994
GDF	GDF8	Primary trophoblast								Invasion	Xie *et al.*, 2020
		Cell lines					HTR8/SVneo			Invasion	Xie *et al.*, 2020; Fang *et al.*, 2021; Chen *et al.*, 2023
	GDF11	Primary trophoblast								Invasion	Wu *et al.*, 2022
		Cell lines						HTR8/SVneo		Invasion	Wu *et al.*, 2022
	GDF15	Placental Explants								Ligand expression	Hong *et al.*, 2022
		Cell lines							HTR8/SVneo	Ligand expression	Hong *et al.*, 2022
										Apoptosis	Li *et al.*, 2014a
										Proliferation	Li *et al.*, 2014a

EVT, extravillous trophoblasts; TSC, trophoblast stem cells.

**Table 4.** Comparison of known models to study TGFβ biology in the placenta trophoblast biology.

Model	Origin	Strengths	Limitations	References
Primary trophoblasts	First and third trimester placenta	Preserve original genetic characteristics Closely resemble the *in vivo* status Accessible samples obtained from term healthy and pathophysiological pregnancies Well established isolation, characterization, and cryopreservation protocols.	Limited proliferation ability Limited self-renewal Mixed cell populations (CTs, STs…) Reduced invasiveness and motility of EVTs at term Difficult to acquire second trimester placental samples Lack of physiological microenvironment and other cell types	Tannetta *et al.*, 2008; Li *et al.*, 2023b; Liu *et al.*, 2023
Placental explants	First and third trimester placenta	Retain the *in vivo* tissue structure and integrity Study cell-to-cell interactions and responses Investigate cellular metabolism under healthy and pathophysiological conditions Flow-cultured explants preserve the tissue intactness	Shorter cultivation time in the static settings Limited self-renewal Genetic manipulation	Kupper *et al.*, 2021; Li *et al.*, 2023b; Liu *et al.*, 2023
Cell lines	Derived from: Choriocarcinoma: JAR JEG-3 Bewo Primary first trimester extravillous trophoblasts transfected with simian virus 40 large T-antigen (SV40T): HTR8/SVneo	Display (some) properties of EVTs Easy to maintain Genetic manipulation Stable and reproducible Time-dependent experimental settings allow dynamic studies of placental development Used to study invasion, proliferation and regulation	Immortalized cells Genetic alterations and phenotypical differences as primary trophoblasts Mimic specific phenotypes of EVTs or STs Limited functional differentiation Cell lines observed signalling do not completely manifest the pathway signalling in primary trophoblasts	Abou-Kheir *et al.*, 2017; Msheik *et al.*, 2019; Pastuschek *et al.*, 2021; Li *et al.*, 2023b; Liu *et al.*, 2023
Trophoblast stem cells (TSC)	Derived from: Human placental trophoblast tissue Human pluripotent stem cells (PSCs)/human embryonic stem cells (ESCs)/human-induced pluripotent stem cells (iPSCs) and human ePSCs stimulated with inducing factors as BMP4	Normal karyotype Unlimited proliferation Multi-directional differentiation Similar gene expression as primary trophoblasts Studies on pathophysiological cells as in PE Easy genetic manipulation Cell fusion/differentiation is inducible	Genetic heterogeneity Widespread imprint erasure High demanding complex culturing process Higher costs	Xu *et al.*, 2002; Koel *et al.*, 2017; Okae *et al.*, 2018; Dong *et al.*, 2020; Sheridan *et al.*, 2021; Liu *et al.*, 2023
Trophoblast organoids	First trimester primary trophoblasts/JEG-3/third trimester trophoblasts/TSC	Structural and transcriptional similarity to placental villi, allowing biological dynamics to mimic developmental processes Capable of proliferation and self-renewal, fusion and secretion of placental hormones Long-term culturing and cryopreservation	Organoids represent expression profile of first trimester placental tissue, therefore might not be suitable as a model for third trimester placenta Combination of primary cells and immortalized cell lines Complex culturing system Costly	Haider *et al.*, 2018; Turco *et al.*, 2018; Chuva de Sousa Lopes *et al.*, 2020; Sheridan *et al.*, 2020; Dietrich *et al.*, 2021; Karvas *et al.*, 2022
Placenta-on-chip	Primary trophoblast/ JEG-3, Co-culture with endothelial cells, fibroblasts	Microfluidics system Control of culture microenvironment Real-time trophoblast differentiation Study of placental barrier, metabolism, transport, and cell-to-cell interactions	Combination of primary cells and immortalized cell lines Costly	Lee *et al.*, 2016; Park *et al.*, 2022; Li *et al.*, 2023b; Liu *et al.*, 2023
Animal models	Rodents, non-human primates	Study TGFβ signalling in the context of tissue microenvironment Investigate *in vivo* responses to TGFβ signalling provides insights into systemic effects	Anatomical and physiological differences between species The structural and functional differences in placental anatomy across pregnancy Genetic manipulation Mimic pathophysiological conditions as PE Costly Ethics	Makris *et al.*, 2016; Marshall *et al.*, 2018; Carter, 2020; Li *et al.*, 2023b

EVT, extravillous trophoblast.

Primary trophoblasts isolated from the first or third trimester placenta are life-like cells for studying TGFβ signalling in placental development, as they retain their original genetic properties (Li *et al.*, 2023b). However, their limited proliferation and lack of self-renewal make genetic manipulation difficult (Tannetta *et al.*, 2008). To overcome this, researchers often combine them with cell lines such as JEG-3, Bewo, JAR, and HTR8/SVneo. Although originated from choriocarcinoma (JEG-3, Bewo, JAR) or transfected with simian virus 40 large T antigen (HTR8/SVneo), these cell lines are often used to study trophoblast differentiation and invasion, because of their ease of maintenance, stability, and reproducibility by genetic manipulation (Li *et al.*, 2023b; Liu *et al.*, 2023). However, it is important to note that immortalized cell lines may have genetic alterations and phenotypic differences compared to primary trophoblasts (Abou-Kheir *et al.*, 2017; Pastuschek *et al.*, 2021). Their *in vitro* properties may not fully reflect *in vivo* signalling, although they are genetically manipulated and reproducible (Msheik *et al.*, 2019; Pastuschek *et al.*, 2021). On the other hand, placental explants, retain the *in vivo* structure for studying cell-to-cell interactions in healthy and pathological pregnancies, such as PE (Li *et al.*, 2023b). When exposed to flow, placental explants retain tissue integrity, further expanding their potential uses (Kupper *et al.*, 2021). However, explants have limitations in terms of difficulties in gene manipulation, lack of self-renewal, and shorter incubation times in static culture (Li *et al.*, 2023b). In addition, explant cultures maintain cells in their tissue context. This offers the advantage to mimic more closely the *in vivo* situation, but may require additional work to attribute observed differences and changes to a specific cell type within the tissue.

TSCs are emerging as a valuable 2D *in vitro* model for studying trophoblast biology. TSCs can be derived from two primary sources: first trimester chorionic villi (CT) and blastocysts (Okae *et al.*, 2018). TSCs are cultured in a specific medium containing activin/nodal/TGFβ pathway inhibitors (e.g. A83-01), Rho-associated protein inhibitors (e.g. Y27632), histone deacetylase inhibitors (e.g. valproic acid), Wnt pathway inducers (e.g. CHIR99021), and epidermal growth factor (EGF) (Okae *et al.*, 2018). This facilitates the long-term expansion of CT-TSCs, which show transcriptional similarities to primary trophoblasts. Forskolin induces TSC fusion into syncytia, and upon removal of Wnt activator, TSCs differentiate into invasive matrix-degrading HLA-G+ EVTs (Okae *et al.*, 2018). Alternatively, TSCs can be derived from hPSCs, including ESCs, iPSCs, or expanded potential stem cells (ePSCs) (Li *et al.*, 2023b). As outlined in the earlier section on BMP signalling in trophoblasts, differentiation induced by BMP4 and activin inhibitors yields TSCs with characteristics similar to primary trophoblasts, although HLA expression patterns may differ from primary trophoblast (Xu *et al.*, 2002; Amita *et al.*, 2013; Koel *et al.*, 2017; Roberts *et al.*, 2018; Horii *et al.*, 2019; Sheridan *et al.*, 2021). Of note, TSCs exhibit normal karyotypes, unlimited proliferation, and versatile differentiation potential, making them adaptable for straightforward genetic manipulation, despite their potential genetic heterogeneity (Li *et al.*, 2023b; Liu *et al.*, 2023).

Development of trophoblast organoids provide an efficient *in vitro* model to study human placental development (Haider *et al.*, 2018; Turco *et al.*, 2018). Trophoblast organoids derived from primary CT cells in early pregnancy show differentiation into EVTs and STs as well as self-renewal capacity (Haider *et al.*, 2018). These organoids secrete placenta-specific peptides such as hCG and GDF15 and closely resemble first trimester trophoblasts (Turco *et al.*, 2018; Sheridan *et al.*, 2021). Cultured on matrigel with TGFβ and BMP signalling inhibitors (A83-01 and Noggin, respectively), EGF, and enhanced Wnt signalling, the organoids show prolonged expansion potential (Haider *et al.*, 2018). Similar to TSCs, removal of the Wnt inducer leads to re-differentiation of ST organoids into HLA-G+ EVTs (Haider *et al.*, 2018). Inhibition of TGFβ supports early invasive EVT development, whereas promotion of TGFβ signalling is critical for mature decidual EVT expression, highlighting the central role of decidual cell-derived TGFβ in controlling the EVT environment (Haider *et al.*, 2022). Trophoblast organoids present notable advantages, including continuous proliferative capacity and the capability to be cryopreserved and thawed (Sheridan *et al.*, 2020). Despite these benefits, a notable limitation involves the inverted architecture of CT and ST within organoids compared to placental villi (Haider *et al.*, 2018). However, this issue can be addressed by cultivating organoids in suspension culture with gentle agitation (Yang *et al.*, 2023). Furthermore, organoids mirror the expression profile of first-trimester placental tissue, potentially limiting their suitability as a model for third-trimester placenta. However, the complex culturing system they require provides an opportunity to study placental pathologies (Chuva de Sousa Lopes *et al.*, 2020; Karvas *et al.*, 2022).

In addition to 3D organoid models, placenta-on-chip models offer precise control of the microenvironment for real-time trophoblast differentiation and placental barrier studies (Lee *et al.*, 2016). While maintaining a placenta-on-a-chip system can increase the costs, the fluid shear stress environment within the system closely mimics *in vivo* conditions and provides an alternative to static cultures. This system allows the study of co-cultures with different cell types to investigate cell-to-cell communication and the placental barrier (Park *et al.*, 2022; Li *et al.*, 2023b; Liu *et al.*, 2023). Unlike the aforementioned organoids, in these on-chip models, first trimester and term placental cells can be used alike, making them a versatile tool for different stages of pregnancy. Animal models, including rodents and non-human primates, allow the study of *in vivo* responses and provide critical insight into the systemic effects of signalling pathways within the tissue environment (Li *et al.*, 2023b). Despite their widespread use, it is important to recognize potential limitations. These can arise from species differences in anatomy and physiology, resulting in structural and functional differences in placental architecture (Carter, 2020). In addition, the challenges of genetic modification, higher costs, and ethical concerns should be considered when using animal models (Makris *et al.*, 2016; Marshall *et al.*, 2018).

### Endothelial cells

The human placenta is a unique source of ECs that are specifically adapted to support vascular needs of the developing foetus. Indeed, several distinct subtypes of first trimester placental EC have been identified, highlighting their functional and metabolic heterogeneity (Zhou *et al.*, 2019). Upregulation of specific TGFβ signalling components (i.e. Smad1, Smad4, BMPR2) has demonstrated the importance of TGFβ in determining EC subtypes and an angiogenic functions (Li *et al.*, 2023a). In general, the TGFβ family maintains the endothelium in a quiescent state and regulates vascular development and barrier function (Possomato-Vieira and Khalil, 2016). Dysregulated TGFβ signalling, together with other factors, leads to the endothelial dysfunction, increased vascular tone, and altered vascular permeability observed in PE (Venkatesha *et al.*, 2006). Among the TGFβ family members, TGFβ1 is one of the most studied factors in endothelial biology, being involved in angiogenesis, cell migration, and blood vessel formation and repair, by inducing tube formation and sprouting (Lebrin *et al.*, 2004; Li *et al.*, 2023a). Indeed, neutralization of TGFβ1 leads to impaired endothelium-mediated vasodilation and increased expression of surface adhesion molecules, resulting in increased leukocyte adhesion (Walshe *et al.*, 2009). Under healthy conditions, TGFβ induces the activation of Smad2/3 signalling via ALK5 and TGFΒRII (Goumans *et al.*, 2003). In addition, the membrane-anchored co-receptor Eng is able to fine tune this complex, by enhancing the affinity for ALK1 and promoting a mixed receptor complex containing ALK1/ALK5 (Goumans *et al.*, 2003; Lebrin *et al.*, 2004). This complex leads to the activation of downstream Smad1/5/8 signalling, thereby promoting angiogenesis (Goumans *et al.*, 2002; Lebrin *et al.*, 2004, 2005). By stimulating different Smad pathways, ALK1 and ALK5 have opposite effects on the behaviour of ECs (Goumans *et al.*, 2002). While ALK5 induces PAI-1, a negative regulator of proliferation and migration, and consequently mediates the production of fibronectin and collagen (Lebrin *et al.*, 2005), ALK1 induces the expression of ID-1, which promotes EC migration and proliferation (Goumans *et al.*, 2002; Lebrin *et al.*, 2005). The interplay between ALK5 and ALK1 allows fine regulation of endothelial function in physiological and pathological processes.

The dysregulation of certain members of the TGFβ family in placental EC biology has attracted considerable attention, particularly with the regard to Eng, a highly upregulated TGFβ accessory receptor that may be associated with endothelial dysfunction (Venkatesha *et al.*, 2006; Leaños-Miranda *et al.*, 2019). Although Eng is essential for vascular homeostasis, its elevated levels in certain inflammatory pathological conditions, such as in PE, contribute to impaired angiogenesis and aberrant vascular development (Venkatesha *et al.*, 2006). During inflammation, MMP12 and/or MMP14 cleave the extracellular domain of Eng, which triggers the excessive release of soluble Eng (sEng) in ECs (Tzavlaki and Moustakas, 2020). By interfering with the binding of TGFβ1 to its receptors TGFβRI and TGFβRII, sEng interrupts with downstream signalling events including inhibiting eNOS activation and vasodilation (Venkatesha *et al.*, 2006). Further, pro-angiogenic factors, such as angiopoietin 1 (Ang-1), vascular endothelial growth factor A (VEGF-A), and fibroblast growth factor receptor 2 (FGR-2) prevent the release of sEng through Akt signalling, a key upstream pathway of VEGF-A (Cudmore *et al.*, 2012). However, in PE, there is an increase in sEng levels, accompanied by increased sFlt-1 (a soluble form of the VEGFR1, encoded by the *FLT1* gene) and decreased Akt phosphorylation, further exacerbating the condition by antagonizing VEGF-A activity and compromising EC (Venkatesha *et al.*, 2006; Cudmore *et al.*, 2012).

The balance between the endothelial BMP and TGFβ signalling pathways is essential in many cardiovascular diseases (Goumans and ten Dijke, 2018). In addition to regulating TGFβ activity, sEng can promote the interaction of BMP9 and BMP10 with ALK1 in EC (Lawera *et al.*, 2019). Interestingly, BMP9 upregulates *ENG* mRNA expression and protein expression in EC, where Eng modulates TGFβ1 function, highlighting the role of Eng as a mediator between BMP and TGFβ signalling in EC (Venkatesha *et al.*, 2006). Although sEng was initially thought to be a ligand trap for BMP9 signalling, preventing BMP9 signalling (Castonguay *et al.*, 2011), Lawera *et al.* (2019) have shown that sEng does not inhibit BMP9 signalling but rather binds BMP9 in a complex and increases the affinity for ALK1, thereby enhancing Smad1/5/8 signalling in ECs. Of note, the sEng/BMP9 complex is highly expressed in PE subjects compared with to normotensive controls. Notably, a negative feedback mechanism has been described whereby sEng stimulates BMP4 expression, which is inhibited by the natural BMP antagonist Noggin. Given that BMP4 contributes to endothelial dysfunction, sEng-BMP4 may be an interesting target for hypertension therapeutics (Gallardo-Vara *et al.*, 2020).

Inflammatory conditions, such as those observed in PE, can trigger signalling pathways and molecular changes that induce endothelial-to-mesenchymal transition (EndoMT) (Derada Troletti *et al.*, 2019). EndoMT is characterized by profound morphological, functional, and molecular changes in the endothelial phenotype (Sánchez-Duffhues *et al.*, 2019). The morphological changes are driven by changes in cell polarity and cytoskeletal rearrangement as ECs differentiate into mesenchymal cells (Ma *et al.*, 2021). During the process of EndoMT, the expression of endothelial markers such as CD31, TIE-1, TIE-2, and VE-cadherin is downregulated, whereas the expression of mesenchymal Vimentin, N-Cadherin, and fibronectin is upregulated (Cho *et al.*, 2018). The phenotypic shift is accompanied by functional changes, such as increased cell migration, and decreased barrier function. It should be noted that EndoMT is a gradual, reversible, and dynamic process, often triggered by paracrine molecules (Ma *et al.*, 2020a). TGFβ signalling is one of the most potent inducers of EndoMT (Ma *et al.*, 2021). Specifically, in human umbilical cord vein ECs (HUVEC), TGFβ1 initiates EndoMT through the phosphorylation of Smad3, leading to the development of the mesenchymal phenotype (Evrard *et al.*, 2016). For example, co-culture of trophoblasts derived from PE placentas and HUVEC of healthy placentas had an effect on the disrupted barrier integrity of the ECs. In addition to observed changes in cell polarity and morphology, the disruption was manifested by the loss of VE-cadherin and occludin, key adhesion molecules (Li *et al.*, 2015b; Wang *et al.*, 2004). Understanding the molecular pathways underlying EndoMT in the context of PE will provide new insights into this pathology that may be exploited for therapeutic gain.

The role of foetal sex linked to effects on the placental endothelium and the development of PE is a subject of increasing research interest. Interestingly, a study conducted by Zhou *et al.* has demonstrated that female HUVECs are more susceptible to the effects of PE, than male HUVECs. This sexual dimorphism of PE affects the endothelium, with PE dysregulating EC migration, impairing vascularization, and disrupting angiogenesis in female foetal ECs. Conversely, in male foetal ECs, PE upregulates genes associated with cell growth failure, blood pressure, and chronic heart failure (Zhou *et al.*, 2019). Remarkably, a manuscript by Zhou showed that TGFβ1 stimulation resulted in increased cell proliferation only in female PE ECs, whereas normotensive controls showed a decreased response. On the other hand, stimulation with TGFβ1 led to a reduction in cell permeability in normotensive female foetal ECs, and not in PE female ECs (Zhou *et al.*, 2019). The identification of sex-specific PE dysregulated gene networks in the foetus has significant implications for early foetal programming and may be associated with the development of cardiovascular disease in children born from PE pregnancies. As endothelial dysfunction is a hallmark of several cardiovascular diseases, an early identification of molecular networks may have a therapeutic potential to prevent long-term cardiovascular risk in individuals exposed to PE during pregnancy.

### Immune cells at the foeto-maternal interface and within the foeto-placental unit

Successful pregnancy requires tolerance between the immune system and the allogeneic foetus (Svensson-Arvelund *et al.*, 2015). Key immune populations at the foeto-maternal interface include regulatory T cells (Tregs), natural killer (NK) cells, and decidual macrophages (dM), while placental macrophages (HBCs) are located in the foetoplacental unit. Despite their location, immune cells coordinate their actions by secreting cytokines to establish immunological tolerance to the foetus, and contribute to vascular remodelling, yet maintain tissue homeostasis (Yagel, 2009; Alijotas-Reig *et al.*, 2014; Reyes *et al.*, 2017; Vondra *et al.*, 2023). TGFβ serves as an important immune regulator during pregnancy (Svensson-Arvelund *et al.*, 2015; Yang *et al.*, 2021). In the first trimester, TGFβ signalling is central to promoting differentiation of Tregs, regulating NK cell function and maintaining the balance between M1 and M2 macrophages at the foeto-maternal interface (Yang *et al.*, 2021). Furthermore, during the third trimester of pregnancy, TGFβ is essential for maintaining tissue homeostasis by regulating an anti-inflammatory phenotype of HBCs (Schliefsteiner *et al.*, 2017, 2020; Mercnik *et al.*, 2022). Perturbances in these regulatory mechanisms are associated with pregnancy complications such as PE or miscarriage (Reyes and Golos, 2018; Yang *et al.*, 2021). Understanding these immune cell interactions and functions is critical to understanding the immune dynamics during pregnancy and their impact on a successful and healthy outcome.

#### Regulatory T-cells

Tregs are pivotal to a successful pregnancy outcome, as they orchestrate maternal tolerance by ‘education’ of other immune cells (Alijotas-Reig *et al.*, 2014). They exist as thymus-derived and peripherally induced Tregs. In pregnancy, specific recruitment of peripheral maternal Tregs to the decidua seems to take place (Tilburgs *et al.*, 2009); after an initial influx of Tregs to the decidua in the first trimester, where Tregs make up about 10% of the leukocyte population, the Treg density plateaus and even decreases again towards term (Williams *et al.*, 2009).

Specifically for the induction of peripheral Tregs, TGFβ is needed (Wilczynski *et al.*, 2008; Yang *et al.*, 2021). FOXP3 expression is hallmark of Tregs, although FOXP3− subsets exist (Krop *et al.*, 2020). TGFβ1induces the production of CD4+/FOXP3+ Tregs, and these Tregs can in turn secrete TGFβ1and participate in immune regulation by acting on other cells and themselves, in para- and autocrine manners (Huang *et al.*, 2020). Of note, several foeto-placental cell types appear to be able to induce Tregs via TGFβ. Ma *et al.* (2020b) found that endovascular EVT released TGFβ which was able to induce maternal Treg formation. Oettel *et al.* (2016) demonstrated that even umbilical ECs release TGFβ which turned maternal T-cells into Tregs, and observed interesting sex-specific differences, with female HUVECs showing a stronger capacity to do so.

Furthermore, TGFβ balances the ratio of Tregs to other T-cell populations; specifically, a tight ratio between Th17 cells and Tregs at the foeto-maternal interface must be maintained for successful pregnancy, as observed from shifts in the Th17/Tregs ratio in patients with recurrent spontaneous miscarriages, and TGFβ is an important factor in its regulation (Wu *et al.*, 2014). A similar shift in TH17/Treg subsets has been observed in PE too, but was not linked to TGFβ in this study (Eghbal-Fard *et al.*, 2019).

In women suffering from PE, changes in Treg frequency and distribution between decidual tissue and periphery have been found (Sasaki *et al.*, 2007). Similar to NK cells, most research on Tregs focusses on Tregs accumulating in the decidua. Quinn *et al.* (2011) specifically investigated decidua from early-onset as opposed to late-onset PE and controls, and found that Tregs were lowest in EOPE. Little data are available as to the presence Tregs within villous tissue. In a recent histopathological study of different placental pathologies, it was found that FOXP3− reactivity within villous tissue was reduced in PE and IUGR compared to controls, while being increased in gestational diabetes (Emirdar *et al.*, 2021).

In summary, Tregs are pivotal for establishing maternal tolerance during pregnancy, orchestrating the immune balance through recruitment and modulation via TGFβ signalling. Imbalances in Treg populations observed in PE, possibly linked with TGFβ signalling, emphasize the crucial role of Treg regulation in maintaining a successful pregnancy.

#### NK cells

NK cells are an integral part of the endometrial innate immune milieu and play an important role in maintaining pregnancy (Yagel, 2009). During pregnancy, the majority of leukocytes are decidual natural killer (dNK) cells. These cells are recruited from the periphery in a CXCR4/CXCL12 dependent manner by EVT cells (Hanna *et al.*, 2003) and then are converted to CD56^bright^/CD16− cells by TGFβ, which is secreted, for example, by decidual stromal cells (Keskin *et al.*, 2007). dNK cells exhibit low cytotoxicity compared to other NK cell subsets and rather contribute to vascular remodelling at the foeto-maternal interface by autocrine release of TGFβ, along with VEGF, angiopoietin, and MMP2 and MMP9 (Lash *et al.*, 2006; Fraser *et al.*, 2012). PE is associated with abnormal TGFβ levels in the decidua (Yang *et al.*, 2021); these elevated levels serve as influential factors modulating dNK cell phenotype and function. Abnormal TGFβ levels, particularly TGFβ1 of Tregs, can disrupt dNK cell function and potentially influence the pathophysiology of PE (Zhang *et al.*, 2019). Further comprehensive insights on dNK cells, particularly their involvement in PE, can be found in recent reviews (Liu *et al.*, 2021; Wei and Yang, 2023). Here we have focused on understanding the role of TGFβ signalling in the context of dNK cells.

However, the plethora of research on NK cells in pregnancy has not focussed on NK cells in the decidua or placenta, but on peripheral NK cells in maternal (and less frequently, foetal cord) blood. For the maternal circulation, there are conflicting data on whether the number of peripheral NK cells is increased, decreased, or maintained at PE, and whether these NK cells are more, less, or as cytotoxic as their counterparts from healthy pregnancies (reviewed in Wei and Yang, 2023). Concerning the foetal circulation, two independent studies supported that in cord blood from PE neonates, activated NK cells appear to be expanded compared to regulatory NK cells, thus initiating an inflammatory response (Sohlberg *et al.*, 2014; Loewendorf *et al.*, 2015). However, neither of these studies looked specifically at TGFβ or BMP signalling and are only mentioned here for completeness.

#### Macrophages

In pregnancy, placental macrophages are a heterogeneous population of immune cells, involved in the maternal–foetal immune regulation, placental cell invasion, angiogenesis, and tissue remodelling (Houser *et al.*, 2011; Reyes and Golos, 2018). Two main types of macrophages are present at all stages of pregnancy. The first type is the decidual macrophages (dM), located in either in the decidua basalis (dBAM) or in the decidua parientalis (dPAM), and the second type are the foeto-placental macrophages, called HBCs (Houser *et al.*, 2011). Regardless of their specific location, their main function is to act as gatekeepers of placental homeostasis (Aneman *et al.*, 2020). Macrophages are keen to polarize into different polarization states in response to microenvironmental stimuli, namely pro-inflammatory M1 and anti-inflammatory M2 (Murray and Wynn, 2011; Murray *et al.*, 2014). The polarization state determines functionality of HBCs, and both dM and HBCs shifts during pregnancy as a function of placental requirements. As the pregnancy progresses, both dM and HBCs develop specific M1/M2 phenotypes, with macrophages in the first trimester tending to exhibit a more pro-inflammatory polarization (Mezouar *et al.*, 2021), but there is a shift towards M2 as pregnancy progresses towards term (Yao *et al.*, 2019). Inappropriate macrophage activation and polarization has been associated with pregnancy disorders such as PE, recurrent pregnancy loss, or chorioamnionitis (Reyes and Golos, 2018). During the first trimester, activated dM secrete a variety of molecules, including tumour necrosis factor (TNF)α and TGFβ1, which may affect regulatory functions of surrounding trophoblasts. In PE, an accumulation of activated dM is observed in proximity of the spiral arteries (Ning *et al.*, 2016). This suggests a potential role for dM in inhibiting trophoblast invasion. M2 polarized dM, secrete high levels of TGFβ1. As discussed above, TGFβ1 is a potent inhibitor of trophoblast invasion and affects the production of MMPs, which are enzymes involved in tissue remodelling (Ning *et al.*, 2016). Conversely, the secretome of EVTs has been shown to regulate the function of dM. In a study by Vondra *et al.* (2023), the secretome of EVTs containing TGFβ induced the proliferation of dBAM, but not dPAM, through the upregulation of macrophage colony stimulating factor (M-CSF). Additionally, factors secreted by EVT such as TGFβ, M-CSF, and IL10 have been implicated in the regulation of M2 polarization of dM (Aldo *et al.*, 2014; Svensson-Arvelund *et al.*, 2015). Trophoblast-derived IL6 has also been shown to induce a specific M2 polarization of dM by activating the STAT3 signalling, resulting in high expression of CD206, CCL18, IL10, and TGFβ (Ding *et al.*, 2021). Furthermore, abnormal levels of decorin, a molecule produced by first trimester dM, have been associated with the development of pregnancy related disorders such as PE, recurrent pregnancy loss, and foetal growth restriction (Nandi *et al.*, 2016). Decorin regulates the toll like receptor 2 (TLR2)/TLR4-MyD88-NF-κB signalling pathway (Wang *et al.*, 2022), which alters mitochondrial metabolism, and promotes a pro-inflammatory M1 phenotype switch of dM, and reduced TGFβ secretion (Wang *et al.*, 2022).

In summary, studies performed during the first trimester highlight the dynamic interaction between EVTs and dM, where both cell types produce high levels of TGFβ and mutually influence each other’s function. Investigating the interplay between EVTs and dM and their modulation of the TGFβ signalling may provide insights into the early pathogenesis of PE, where TGFβ signalling is impaired.

During the third trimester of pregnancy, TGFβ acts as vital immunoregulator, preserving the homeostatic phenotype of macrophages (Svensson-Arvelund *et al.*, 2015). TGFβs, as major drivers of M2 polarization, are regulators of the function of dM and HBCs (Murray *et al.*, 2014). Both, dM and HBCs constitutively express TGFβ1 (Schliefsteiner *et al.*, 2017, 2020; Pavlov *et al.*, 2020; Mercnik *et al.*, 2022). While dMs only produce the pro-form of TGFβ1, HBCs also secrete the active form of TGFβ1 (Schliefsteiner *et al.*, 2017; Pavlov *et al.*, 2020; Mercnik *et al.*, 2022). In addition, dPAM and dBAM also secrete TGFβ2 (Vondra *et al.*, 2023). HBCs secrete TGFβ2 and TGFβ3, although TGFβ1 is the major ligand secreted by HBCs (Pavlov *et al.*, 2020). The contribution of TGFβ to the tolerogenic and homeostatic functions of HBCs has been demonstrated particularly in the context of infections and inflammatory pathologies that occur during the pregnancy (Azari *et al.*, 2021). For example, during infection with *Lysteria monocytogeneses*, HBCs undergo pro-inflammatory reprograming. At the same time however, HBCs increase the secretion of anti-inflammatory TGFβ1, which serves to prevent maternal anti-foetal adaptive immunity and further promotes the anti-inflammatory environment (Azari *et al.*, 2021). Additionally, the immunoregulatory actions of TGFβ1 by HBCs have been found to limit HIV-1 replication in HBCs when exposed to exogenous TGFβ (Johnson and Chakraborty, 2012). Furthermore, the increased secretion of TGFβ1 in EO—and LOPE HBCs is associated with the maintenance of anti-inflammatory phenotype of both inflammatory conditions, as TGFβ is widely recognized for its role in resolving inflammation (Mercnik *et al.*, 2022).

These findings highlight the important role of TGFβ signalling in modulating the immune responses and maintaining the immune homeostasis in HBCs during the pregnancy and in providing protection against excessive inflammation such as in PE.

## Conclusion and future perspectives

**Figure 3.**
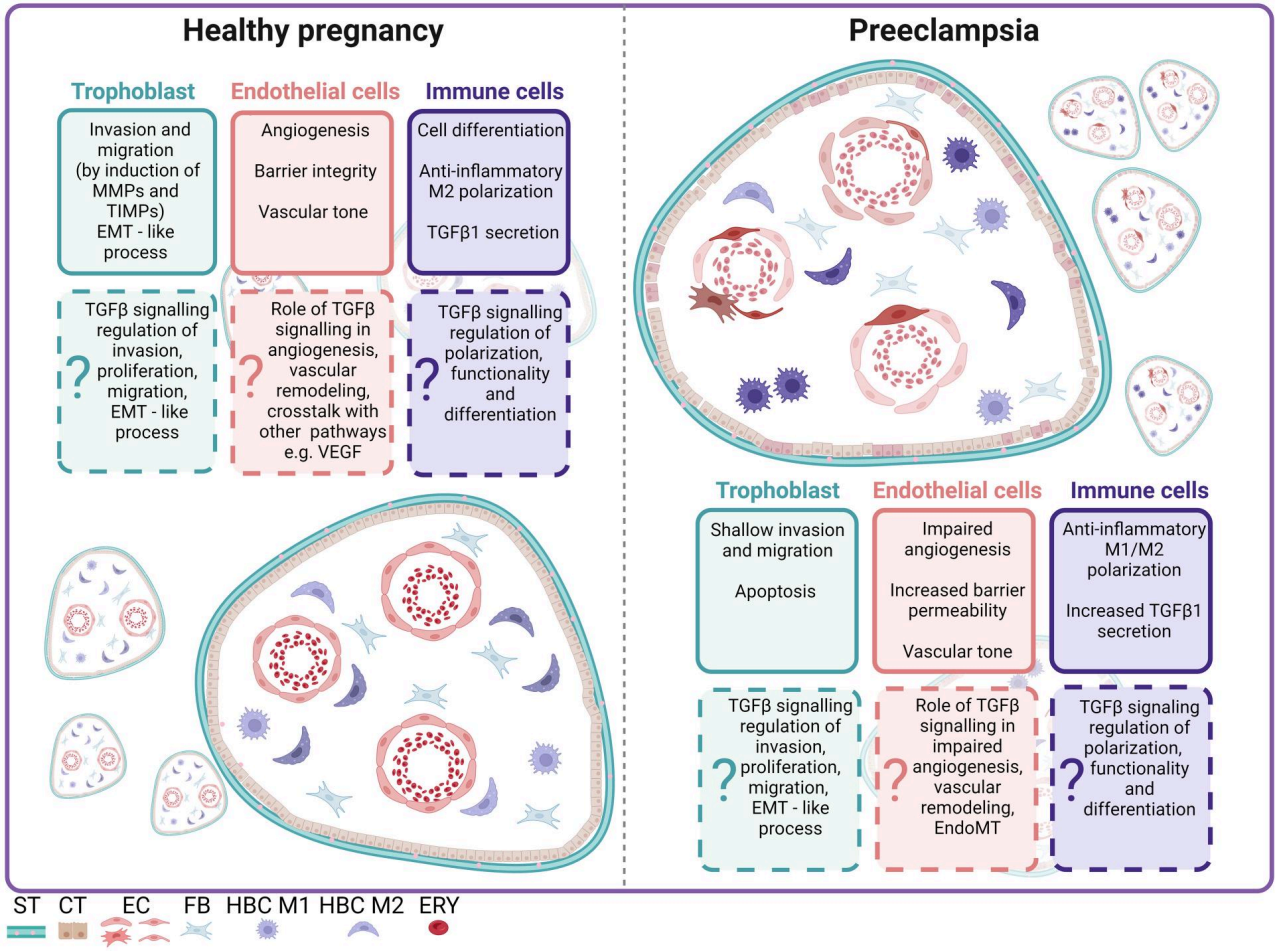
**The unequal importance of TGFβ signalling in PE-compromised placental cells.** The figure provides a cross-sectional representation of the placental villous structure, illustrating a comparison between a healthy (CTR) placenta and a preeclamptic (PE) placenta. The main placental cell types, including trophoblasts, ECs, and immune cells, are depicted within the structure, highlighting their specific known roles in the respective placenta. Question marks (?) indicate the unknown and challenging aspects of TGFβ signalling in these specific cell types. These questions relate to the precise mechanisms and effects of TGFβ signalling in trophoblast function, the impaired vascular remodelling and angiogenesis of ECs, and the phenotypic changes observed in immune cells during PE. FB, fibroblasts; HBC M1, M1 polarized Hofbauer cells; HBC M2, M2 polarized Hofbauer cells; ERY, erythrocytes.

We have summarized how TGFβ signalling plays an essential spatio-temporal role in regulating the function of all placental cells, including trophoblasts, foeto-placental EC, and immune cells. TGFβ ligands contribute to processes such as trophoblast invasion, vascularization, immune tolerance, and tissue remodelling, to ensure successful placental development during pregnancy (Dietrich *et al.*, 2022; Li *et al.*, 2023a). Dysregulation of TGFβ signalling has been linked to the pathogenesis of PE, a complex disorder characterized by shallow trophoblast invasion, defective vascular remodelling, decreased uteroplacental perfusion (Haider *et al.*, 2017), and impaired EC (Zhou *et al.*, 2019) and macrophages (Mercnik *et al.*, 2022). Despite significant progress, there are still many unknowns and challenges in understanding TGFβ signalling in the context of PE (Fig. 3). In trophoblasts, the specific mechanisms by which TGFβ ligands regulate invasion and EMT-like process remain poorly understood. In EC, the precise role of TGFβ signalling in impaired vascular remodelling, angiogenesis, and possible endothelial-to-mesenchymal transition (EndoMT) requires further investigation. TGFβ serves as an important immune regulator during pregnancy and plays a central role in Treg differentiation and regulation of NK cell function, which is poorly understood. In macrophages, the influence of TGFβ signalling on polarization, cytokine production, and tissue remodelling during PE remains to be elucidated. Further research is needed to unravel the dysregulation of TGFβ ligands, the regulation of receptor activation, the crosstalk with other signalling pathways, and the epigenetic regulation in PE. Of note, distinguishing between the different clinically manifest subtypes of PE is critical to advancing our understanding of TGFβ signalling in this syndrome. Understanding the interactions and dysregulation of TGFβ signalling in these cell types is essential to uncover the underlying mechanisms that contribute to the development of PE. This knowledge may facilitate the development of improved *in vitro* and *in vivo* models to study PE and identify potential therapeutic targets within the TGFβ signalling pathway.

## Data Availability

There are no new data associated with this article.

## References

[dmae007-B1] Abdollah S , Macías-Silva M, Tsukazaki T, Hayashi H, Attisano L, Wrana JL. TβRI phosphorylation of Smad2 on Ser465 and Ser467 is required for Smad2-Smad4 complex formation and signaling. J Biol Chem 1997;272:27678–27685. 9346908 10.1074/jbc.272.44.27678

[dmae007-B2] Abou-Kheir W , Barrak J, Hadadeh O, Daoud G. HTR-8/SVneo cell line contains a mixed population of cells. Placenta 2017;50:1–7. 28161053 10.1016/j.placenta.2016.12.007

[dmae007-B3] Adu-Gyamfi EA , Ding YB, Wang YX. Regulation of placentation by the transforming growth factor beta superfamily. Biol Reprod 2020;102:18–26. 31566220 10.1093/biolre/ioz186

[dmae007-B4] Aldo PB , Racicot K, Craviero V, Guller S, Romero R, Mor G. Trophoblast induces monocyte differentiation into CD14+/CD16+ macrophages. Am J Reprod Immunol 2014;72:270–284. 24995492 10.1111/aji.12288 PMC4230492

[dmae007-B5] Alijotas-Reig J , Llurba E, Gris JM. Potentiating maternal immune tolerance in pregnancy: a new challenging role for regulatory T cells. Placenta 2014;35:241–248. 24581729 10.1016/j.placenta.2014.02.004

[dmae007-B6] Amita M , Adachi K, Alexenko AP, Sinha S, Schust DJ, Schulz LC, Roberts RM, Ezashi T. Complete and unidirectional conversion of human embryonic stem cells to trophoblast by BMP4. Proc Natl Acad Sci USA 2013;110:E1212–E1221. 23493551 10.1073/pnas.1303094110 PMC3612666

[dmae007-B7] Aneman I , Pienaar D, Suvakov S, Simic TP, Garovic VD, McClements L. Mechanisms of key innate immune cells in early- and late-onset preeclampsia. Front Immunol 2020;11:1864. 33013837 10.3389/fimmu.2020.01864 PMC7462000

[dmae007-B8] Arutyunyan A , Roberts K, Troulé K, Wong FCK, Sheridan MA, Kats I, Garcia-Alonso L, Velten B, Hoo R, Ruiz-Morales ER et al Spatial multiomics map of trophoblast development in early pregnancy. Nature 2023;616:143–151. 36991123 10.1038/s41586-023-05869-0 PMC10076224

[dmae007-B9] Assoian RK , Komoriya A, Meyers CA, Miller DM, Sporn MB. Transforming growth factor-beta in human platelets. Identification of a major storage site, purification, and characterization. J Biol Chem 1983;258:7155–7160. 6602130

[dmae007-B10] Azari S , Johnson LJ, Webb A, Kozlowski SM, Zhang X, Rood K, Amer A, Seveau S. Hofbauer cells spread listeria monocytogenes among placental cells and undergo pro-inflammatory reprogramming while retaining production of tolerogenic factors. mBio 2021;12:e0184921. 34399615 10.1128/mBio.01849-21 PMC8406333

[dmae007-B11] Ball E , Robson SC, Ayis S, Lyall F, Bulmer JN. Expression of TGF beta in the placental bed is not altered in sporadic miscarriage. Placenta 2007;28:965–971. 17531316 10.1016/j.placenta.2007.03.004

[dmae007-B12] Barbara NP , Wrana JL, Letarte M. Endoglin is an accessory protein that interacts with the signaling receptor complex of multiple members of the transforming growth factor-β superfamily. J Biol Chem 1999;274:584–594. 9872992 10.1074/jbc.274.2.584

[dmae007-B13] Barber CV , Yo JH, Rahman RA, Wallace EM, Palmer KR, Marshall SA. Activin A and pathologies of pregnancy: a review. Placenta 2023;136:35–41. 37028223 10.1016/j.placenta.2023.03.008

[dmae007-B14] Bearfield C , Jauniaux E, Groome N, Sargent IL, Muttukrishna S. The secretion and effect of inhibin A, activin A and follistatin on first-trimester trophoblasts *in vitro*. Eur J Endocrinol 2005;152:909–916. 15941932 10.1530/eje.1.01928

[dmae007-B15] Benian A , Madazli R, Aksu F, Uzun H, Aydin S. Plasma and placental levels of interleukin-10, transforming growth factor-β1, and epithelial-cadherin in preeclampsia. Obstet Gynecol 2002;100:327–331. 12151158 10.1016/s0029-7844(02)02077-x

[dmae007-B16] Bidart M , Ricard N, Levet S, Samson M, Mallet C, David L, Subileau M, Tillet E, Feige JJ, Bailly S. BMP9 is produced by hepatocytes and circulates mainly in an active mature form complexed to its prodomain. Cell Mol Life Sci 2012;69:313–324. 21710321 10.1007/s00018-011-0751-1 PMC11114909

[dmae007-B17] Bragdon B , Moseychuk O, Saldanha S, King D, Julian J, Nohe A. Bone morphogenetic proteins: a critical review. Cell Signal 2011;23:609–620. 20959140 10.1016/j.cellsig.2010.10.003

[dmae007-B18] Brosens I , Pijnenborg R, Vercruysse L, Romero R. The “Great Obstetrical Syndromes” are associated with disorders of deep placentation. Am J Obstet Gynecol 2011;204:193–201. 21094932 10.1016/j.ajog.2010.08.009 PMC3369813

[dmae007-B501] Budi EH , Duan D, Derynck R. Transforming Growth Factor-β Receptors and Smads: Regulatory Complexity and Functional Versatility. Trends Cell Biol 2017;27:658–672. 28552280 10.1016/j.tcb.2017.04.005

[dmae007-B19] Burton GJ , Woods AW, Jauniaux E, Kingdom JCP. Rheological and physiological consequences of conversion of the maternal spiral arteries for uteroplacental blood flow during human pregnancy. Placenta2009a;30:473–482. 19375795 10.1016/j.placenta.2009.02.009 PMC2697319

[dmae007-B20] Burton GJ , Yung HW, Cindrova-Davies T, Charnock-Jones DS. Placental endoplasmic reticulum stress and oxidative stress in the pathophysiology of unexplained intrauterine growth restriction and early onset preeclampsia. Placenta2009b;30(Suppl A):S43–S48. 19081132 10.1016/j.placenta.2008.11.003 PMC2684656

[dmae007-B500] Caniggia I , Grisaru-Gravnosky S, Kuliszewsky M, Post M, Lye SJ. Inhibition of TGF-beta 3 restores the invasive capability of extravillous trophoblasts in preeclamptic pregnancies. J Clin Invest 1999;103:1641–1650. 10377170 10.1172/JCI6380 PMC408387

[dmae007-B21] Caniggia I , Lye SJ, Cross JC. Activin is a local regulator of human cytotrophoblast cell differentiation. Endocrinology1997a;138:3976–3986. 9275089 10.1210/endo.138.9.5403

[dmae007-B22] Caniggia I , Taylor CV, Ritchie JWK, Lye SJ, Letarte M. Endoglin regulates trophoblast differentiation along the invasive pathway in human placental villous explants. Endocrinology1997b;138:4977–4988. 9348229 10.1210/endo.138.11.5475

[dmae007-B23] Carter AM. Animal models of human pregnancy and placentation: alternatives to the mouse. Reproduction 2020;160:R129–R143. 33112767 10.1530/REP-20-0354

[dmae007-B24] Castonguay R , Werner ED, Matthews RG, Presman E, Mulivor AW, Solban N, Sako D, Pearsall RS, Underwood KW, Seehra J et al Soluble endoglin specifically binds bone morphogenetic proteins 9 and 10 via its orphan domain, inhibits blood vessel formation, and suppresses tumor growth. J Biol Chem 2011;286:30034–30046. 21737454 10.1074/jbc.M111.260133 PMC3191044

[dmae007-B25] Chen J , Song T, Yang S, Meng Q, Han X, Wu Z, Cheng JC, Fang L. Snail mediates GDF-8-stimulated human extravillous trophoblast cell invasion by upregulating MMP2 expression. Cell Commun Signal 2023;21:1–13. 37143106 10.1186/s12964-023-01107-2 PMC10158255

[dmae007-B26] Chen X , Liu Y, Xu X, Chen H. Correlation of Cyr61 and CTGF in placentas from the late pre-eclamptic pregnancy. J Perinat Med 2012;40:199–200. 22905356 10.1515/jpm-2011-0035

[dmae007-B27] Cheng JC , Chang HM, Leung PC. TGF-β1 inhibits human trophoblast cell invasion by upregulating connective tissue growth factor expression. Endocrinology 2017;158:3620–3628. 28977597 10.1210/en.2017-00536

[dmae007-B28] Cheng JC , Chang HM, Leung PCK. Transforming growth factor-β1 inhibits trophoblast cell invasion by inducing snail-mediated down-regulation of vascular endothelial-cadherin protein. J Biol Chem 2013;288:33181–33192. 24106276 10.1074/jbc.M113.488866 PMC3829165

[dmae007-B29] Cho JG , Lee A, Chang W, Lee MS, Kim J. Endothelial to mesenchymal transition represents a key link in the interaction between inflammation and endothelial dysfunction. Front Immunol 2018;9:294. 29515588 10.3389/fimmu.2018.00294 PMC5826197

[dmae007-B30] Chuva de Sousa Lopes SM , Alexdottir MS, Valdimarsdottir G. The TGFβ family in human placental development at the fetal-maternal interface. Biomolecules 2020;10:453. 32183218 10.3390/biom10030453 PMC7175362

[dmae007-B31] Ciarmela P , Florio P, Toti P, Franchini A, Maguer-Satta V, Ginanneschi C, Ottavian E, Petraglia F. Human placenta and fetal membranes express follistatin-related gene mRNA and protein. J Endocrinol Invest 2003;26:641–645. 14594115 10.1007/BF03347022

[dmae007-B32] Cindrova-Davies T , Sferruzzi-Perri AN. Human placental development and function. Semin Cell Dev Biol 2022;131:66–77. 35393235 10.1016/j.semcdb.2022.03.039

[dmae007-B33] Cowden Dahl KD , Fryer BH, Mack FA, Compernolle V, Maltepe E, Adelman DM, Carmeliet P, Simon MC. Hypoxia-inducible factors 1α and 2α regulate trophoblast differentiation. Mol Cell Biol 2005;25:10479–10491. 16287860 10.1128/MCB.25.23.10479-10491.2005 PMC1291235

[dmae007-B34] Cudmore MJ , Ahmad S, Sissaoui S, Ramma W, Ma B, Fujisawa T, Al-Ani B, Wang K, Cai M, Crispi F et al Loss of Akt activity increases circulating soluble endoglin release in preeclampsia: identification of inter-dependency between Akt-1 and heme oxygenase-1. Eur Heart J 2012;33:1150–1158. 21411816 10.1093/eurheartj/ehr065 PMC3341632

[dmae007-B35] De Caestecker MP , Parks WT, Frank CJ, Castagnino P, Bottaro DP, Roberts AB, Lechleider RJ. Smad2 transduces common signals from receptor serinethreonine and tyrosine kinases. Genes Dev 1998;12:1587–1592. 9620846 10.1101/gad.12.11.1587 PMC316877

[dmae007-B36] Debieve F , Pampfer S, Thomas K. Inhibin and activin production and subunit expression in human placental cells cultured *in vitro*. Mol Hum Reprod 2000;6:743–749. 10908285 10.1093/molehr/6.8.743

[dmae007-B37] Deng J , Zhao HJ, Zhong Y, Hu C, Meng J, Wang C, Lan X, Wang X, Chen ZJ, Yan J et al H3K27me3-modulated Hofbauer cell BMP2 signalling enhancement compensates for shallow trophoblast invasion in preeclampsia. EBioMedicine 2023;93:104664. 37331163 10.1016/j.ebiom.2023.104664 PMC10300321

[dmae007-B38] Derada Troletti C , Fontijn RD, Gowing E, Charabati M, van Het Hof B, Didouh I, van der Pol SMA, Geerts D, Prat A, van Horssen J et al Inflammation-induced endothelial to mesenchymal transition promotes brain endothelial cell dysfunction and occurs during multiple sclerosis pathophysiology. Cell Death Dis 2019;10:45–13. 30718504 10.1038/s41419-018-1294-2 PMC6361981

[dmae007-B39] Derynck R , Budi EH. Specificity, versatility, and control of TGF-B family signaling. Sci Signal 2019;12:eaav5183. 30808818 10.1126/scisignal.aav5183 PMC6800142

[dmae007-B40] Derynck R , Zhang YE. Smad-dependent and Smad-independent pathways in TGF-B family signalling. Nature 2003;425:577–584. 14534577 10.1038/nature02006

[dmae007-B41] Dietrich B , Haider S, Meinhardt G, Pollheimer J, Knöfler M. WNT and NOTCH signaling in human trophoblast development and differentiation. Cell Mol Life Sci 2022;79:1–16. 10.1007/s00018-022-04285-3 PMC9106601 35562545

[dmae007-B42] Dietrich B , Kunihs V, Haider S, Pollheimer J, Knöfler M. 3-Dimensional JEG-3 choriocarcinoma cell organoids as a model for trophoblast expansion and differentiation. Placenta 2021;104:243–246. 33461068 10.1016/j.placenta.2020.12.013

[dmae007-B43] Ding J , Yang C, Cheng Y, Wang J, Zhang S, Yan S, He F, Yin T, Yang J. Trophoblast-derived IL-6 serves as an important factor for normal pregnancy by activating Stat3-mediated M2 macrophages polarization. Int Immunopharmacol 2021;90:106788. 32718866 10.1016/j.intimp.2020.106788

[dmae007-B44] Dirisipam K , Madduru D, Jahan P, Gujrati D. Can circulating levels of transforming growth factor-β1 in early pregnancy serve as a predictive marker of unfavourable outcome? Placenta 2023;137:65–69. 37086573 10.1016/j.placenta.2023.04.004

[dmae007-B45] Djurovic S , Schjetlein R, Wisløff F, Haugen G, Husby H, Berg K. Plasma concentrations of Lp(a) lipoprotein and TGF-β1 are altered in preeclampsia. Clin Genet 1997;52:371–376. 9520129 10.1111/j.1399-0004.1997.tb04356.x

[dmae007-B46] Dong C , Beltcheva M, Gontarz P, Zhang B, Popli P, Fischer LA, Khan SA, Park KM, Yoon EJ, Xing X et al Derivation of trophoblast stem cells from naïve human pluripotent stem cells. Elife 2020;9:e52504. 32048992 10.7554/eLife.52504 PMC7062471

[dmae007-B47] Davies JE , Pollheimer J, Yong HEJ, Kokkinos MI, Kalionis B, Knöfler M, Murthi P. Epithelial-mesenchymal transition during extravillous trophoblast differentiation. Cell Adh Migr 2016;10:310–321. 27070187 10.1080/19336918.2016.1170258 PMC4951171

[dmae007-B48] Eghbal-Fard S , Yousefi M, Heydarlou H, Ahmadi M, Taghavi S, Movasaghpour A, Jadidi-Niaragh F, Yousefi B, Dolati S, Hojjat-Farsangi M et al The imbalance of Th17/Treg axis involved in the pathogenesis of preeclampsia. J Cell Physiol 2019;234:5106–5116. 30277561 10.1002/jcp.27315

[dmae007-B49] Emirdar V , Ekizceli G, Dilber Y, Inan S, Sanci M. Immunolocalization of FOXP3, JAK1 and STAT5 in preeclamptic, intrauterine growth restricted and gestational diabetic human placentas. Aegean J Obstet Gynecol 2021;3:73–77.

[dmae007-B50] Evrard SM , Lecce L, Michelis KC, Nomura-Kitabayashi A, Pandey G, Purushothaman K-R, d’Escamard V, Li JR, Hadri L, Fujitani K et al Endothelial to mesenchymal transition is common in atherosclerotic lesions and is associated with plaque instability. Nat Commun 2016;7:11853–11816. 27340017 10.1038/ncomms11853 PMC4931033

[dmae007-B51] Fang L , Wang Z, Wu Z, Yan Y, Gao Y, Li Y, Cheng JC, Sun YP. GDF-8 stimulates trophoblast cell invasion by inducing ALK5-SMAD2/3-mediated MMP2 expression. Reproduction 2021;162:331–338. 34432647 10.1530/REP-21-0197

[dmae007-B52] Fang L , Yan Y, Gao Y, Wu Z, Wang Z, Yang S, Cheng JC, Sun YP. TGF-β1 inhibits human trophoblast cell invasion by upregulating kisspeptin expression through ERK1/2 but not SMAD signaling pathway. Reprod Biol Endocrinol 2022;20:1–12. 35101033 10.1186/s12958-022-00902-9 PMC8802482

[dmae007-B53] Ferreira LC , Gomes CEM, Araújo ACP, Bezerra PF, Duggal P, Jeronimo SMB. Association between ACVR2A and early-onset preeclampsia: replication study in a Northeastern Brazilian population. Placenta 2015;36:186–190. 25499008 10.1016/j.placenta.2014.11.007

[dmae007-B54] Florio P , Luisi S, Ciarmela P, Severi FM, Bocchi C, Petraglia F. Inhibins and activins in pregnancy. Mol Cell Endocrinol 2004;225:93–100. 15451573 10.1016/j.mce.2004.02.018

[dmae007-B55] Forbes K , Souquet B, Garside R, Aplin JD, Westwood M. Transforming growth factor-β (TGFβ) receptors I/II differentially regulate TGFβ1 and IGF-binding protein-3 mitogenic effects in the human placenta. Endocrinology 2010;151:1723–1731. 20172969 10.1210/en.2009-0896

[dmae007-B56] Fraser R , Whitley GS, Johnstone AP, Host AJ, Sebire NJ, Thilaganathan B, Cartwright JE. Impaired decidual natural killer cell regulation of vascular remodelling in early human pregnancies with high uterine artery resistance. J Pathol 2012;228:322–332. 22653829 10.1002/path.4057 PMC3499663

[dmae007-B57] Fu G , Ye G, Nadeem L, Ji L, Manchanda T, Wang Y, Zhao Y, Qiao J, Wang YL, Lye S et al MicroRNA-376c impairs transforming growth factor-β and nodal signaling to promote trophoblast cell proliferation and invasion. Hypertension 2013;61:864–872. 23424236 10.1161/HYPERTENSIONAHA.111.203489

[dmae007-B58] Fuentealba LC , Eivers E, Ikeda A, Hurtado C, Kuroda H, Pera EM, De Robertis EM. Integrating patterning signals: Wnt/GSK3 regulates the duration of the BMP/Smad1 signal. Cell 2007;131:980–993. 18045539 10.1016/j.cell.2007.09.027 PMC2200633

[dmae007-B59] Gallardo-Vara E , Gamella-Pozuelo L, Perez-Roque L, Bartha JL, Garcia-Palmero I, Casal JI, López-Novoa JM, Pericacho M, Bernabeu C. Potential role of circulating endoglin in hypertension via the upregulated expression of BMP4. Cells 2020;9:988. 32316263 10.3390/cells9040988 PMC7226995

[dmae007-B60] Gauster M , Moser G, Wernitznig S, Kupper N, Huppertz B. Early human trophoblast development: from morphology to function. Cell Mol Life Sci 2022;79:1–18. 10.1007/s00018-022-04377-0 PMC9167809 35661923

[dmae007-B61] Gestational Hypertension and Preeclampsia: ACOG Practice Bulletin, Number 222. Obstet Gynecol 2020;135:e237–e260. 32443079 10.1097/AOG.0000000000003891

[dmae007-B62] Gonzalez DM , Medici D. Signaling mechanisms of the epithelial-mesenchymal transition. Sci Signal 2014;7:re8. 25249658 10.1126/scisignal.2005189 PMC4372086

[dmae007-B63] Goumans MJ , ten Dijke P. TGF-β signaling in control of cardiovascular function. Cold Spring Harb Perspect Biol 2018;10:a022210. 28348036 10.1101/cshperspect.a022210 PMC5793760

[dmae007-B64] Goumans MJ , Valdimarsdottir G, Itoh S, Lebrin F, Larsson J, Mummery C, Karlsson S, ten Dijke P. Activin receptor-like kinase (ALK)1 is an antagonistic mediator of lateral TGFβ/ALK5 signaling. Mol Cell 2003;12:817–828. 14580334 10.1016/s1097-2765(03)00386-1

[dmae007-B65] Goumans MJ , Valdimarsdottir G, Itoh S, Rosendahl A, Sideras P, Ten Dijke P. Balancing the activation state of the endothelium via two distinct TGF-β type I receptors. EMBO J 2002;21:1743–1753. 11927558 10.1093/emboj/21.7.1743 PMC125949

[dmae007-B66] Graham CH. Effect of transforming growth factor-β on the plasminogen activator system in cultured first trimester human cytotrophoblasts. Placenta 1997;18:137–143. 9089774 10.1016/s0143-4004(97)90085-0

[dmae007-B67] Gregory AL , Xu G, Sotov V, Letarte M. Review: the enigmatic role of endoglin in the placenta. Placenta 2014;35(Suppl):S93–S99. 24252708 10.1016/j.placenta.2013.10.020

[dmae007-B68] Guo J , Tian T, Lu D, Xia G, Wang H, Dong M. Alterations of maternal serum and placental follistatin-like 3 and myostatin in pre-eclampsia. J Obstet Gynaecol Res 2012;38:988–996. 22568578 10.1111/j.1447-0756.2011.01823.x

[dmae007-B69] Haider S , Beristain AG. Human organoid systems in modeling reproductive tissue development, function, and disease. Hum Reprod 2023;38:1449–1463. 37119533 10.1093/humrep/dead085

[dmae007-B70] Haider S , Kunihs V, Fiala C, Pollheimer J, Knöfler M. Expression pattern and phosphorylation status of Smad2/3 in different subtypes of human first trimester trophoblast. Placenta 2017;57:17–25. 28864007 10.1016/j.placenta.2017.06.003

[dmae007-B71] Haider S , Lackner AI, Dietrich B, Kunihs V, Haslinger P, Meinhardt G, Maxian T, Saleh L, Fiala C, Pollheimer J. Transforming growth factor-β signaling governs the differentiation program of extravillous trophoblasts in the developing human placenta. Proc Natl Acad Sci USA 2022;119:e2120667119. 35867736 10.1073/pnas.2120667119 PMC9282384

[dmae007-B72] Haider S , Meinhardt G, Saleh L, Kunihs V, Gamperl M, Kaindl U, Ellinger A, Burkard TR, Fiala C, Pollheimer J et al Self-renewing trophoblast organoids recapitulate the developmental program of the early human placenta. Stem Cell Reports 2018;11:537–551. 30078556 10.1016/j.stemcr.2018.07.004 PMC6092984

[dmae007-B73] Hanna J , Wald O, Goldman-Wohl D, Prus D, Markel G, Gazit R, Katz G, Haimov-Kochman R, Fujii N, Yagel S et al CXCL12 expression by invasive trophoblasts induces the specific migration of CD16− human natural killer cells. Blood 2003;102:1569–1577. 12730110 10.1182/blood-2003-02-0517

[dmae007-B74] Heldin C-H , Miyazono K, ten Dijke P. TGF-signalling from cell membrane to nucleus through SMAD proteins. Nature 1997;390:465–471. 9393997 10.1038/37284

[dmae007-B75] Heldin CH , Moustakas A. Signaling receptors for TGF-β family members. Cold Spring Harb Perspect Biol 2016;8:a022053. 27481709 10.1101/cshperspect.a022053 PMC4968163

[dmae007-B76] Hennessy A , Orange S, Willis N, Painter DM, Child A, Horvath JS. Transforming growth factor-β1 does not relate to hypertension in pre-eclampsia. Clin Exp Pharmacol Physiol 2002;29:968–971. 12366387 10.1046/j.1440-1681.2002.03763.x

[dmae007-B77] Henry-Berger J , Mouzat K, Baron S, Bernabeu C, Marceau G, Saru JP, Sapin V, Lobaccaro JMA, Caira F. Endoglin (CD105) expression is regulated by the liver X receptor alpha (NR1H3) in human trophoblast cell line JAR. Biol Reprod 2008;78:968–975. 18276933 10.1095/biolreprod.107.066498

[dmae007-B78] Hill CS. Transcriptional control by the SMADs. Cold Spring Harb Perspect Biol 2016;8:a022079. 27449814 10.1101/cshperspect.a022079 PMC5046698

[dmae007-B79] Hong W , Chen J-H, Ma H-J, Li X-CLi-Li. Fragile X-related protein 1 (FXR1) promotes trophoblast migration at early pregnancy via downregulation of GDF-15 expression. Reprod Sci 2022;29:110–121. 34291416 10.1007/s43032-021-00693-1 PMC8677690

[dmae007-B80] Horii M , Bui T, Touma O, Cho HY, Parast MM. An improved two-step protocol for trophoblast differentiation of human pluripotent stem cells. Curr Protoc Stem Cell Biol 2019;50:e96. 31479595 10.1002/cpsc.96 PMC6730560

[dmae007-B81] Horii M , Touma O, Bui T, Parast MM. Modeling human trophoblast, the placental epithelium at the maternal fetal interface. Reproduction 2020;160:R1–R11. 32485667 10.1530/REP-19-0428 PMC7286067

[dmae007-B82] Hough C , Radu M, Doré JJE. TGF-beta induced Erk phosphorylation of Smad linker region regulates Smad signaling. PLoS One 2012;7:e42513. 22880011 10.1371/journal.pone.0042513 PMC3412844

[dmae007-B83] Houser BL , Tilburgs T, Hill J, Nicotra ML, Strominger JL. Two unique human decidual macrophage populations. J Immunol 2011;186:2633–2642. 21257965 10.4049/jimmunol.1003153 PMC3712354

[dmae007-B84] Huang N , Chi H, Qiao J. Role of regulatory T cells in regulating fetal-maternal immune tolerance in healthy pregnancies and reproductive diseases. Front Immunol 2020;11:1023. 32676072 10.3389/fimmu.2020.01023 PMC7333773

[dmae007-B85] Huang T , Schor SL, Hinck AP. Biological activity differences between TGF-β1 and TGF-β3 correlate with differences in the rigidity and arrangement of their component monomers terms of use. Biochemistry 2014;53:5749. 10.1021/bi500647d PMC4165442 25153513

[dmae007-B8500] Huber A, , Hefler L, , Tempfer C, , Zeisler H, , Lebrecht A, , Husslein P. Transforming growth factor-beta 1 serum levels in pregnancy and pre-eclampsia. Acta Obstet Gynecol Scand 2002;81:168–171. 11942909 10.1034/j.1600-0412.2002.810214.x

[dmae007-B86] Huppertz B. The anatomy of the normal placenta. J Clin Pathol2008a;61:1296–1302. 18755720 10.1136/jcp.2008.055277

[dmae007-B87] Huppertz B. Placental origins of preeclampsia: challenging the current hypothesis. Hypertension2008b;51:970–975. 18259009 10.1161/HYPERTENSIONAHA.107.107607

[dmae007-B88] Incebiyik A , Kocarslan S, Camuzcuoglu A, Hilali NG, Incebiyik H, Camuzcuoglu H. Trophoblastic E-cadherin and TGF-beta expression in placenta percreta and normal pregnancies. J Matern Fetal Neonatal Med 2014;29:126–129. 25471088 10.3109/14767058.2014.989203

[dmae007-B89] Ingman WV , Robertson SA. The essential roles of TGFB1 in reproduction. Cytokine Growth Factor Rev 2009;20:233–239. 19497778 10.1016/j.cytogfr.2009.05.003

[dmae007-B502] Io S , Kabata MIO, Iemura Y, Semi K, Morone N, Minagawa A, Wang BO, Okamoto I, Nakamura T, Kojima Y et al Capturing human trophoblast development with naive pluripotent stem cells in vitro. Cell Stem Cell 2021;28:1023–1039.e13. 33831365 10.1016/j.stem.2021.03.013

[dmae007-B90] Irving JA , Lala PK. Functional role of cell surface integrins on human trophoblast cell migration: regulation by TGF-β, IGF-II, and IGFBP-1. Exp Cell Res 1995;217:419–427. 7535237 10.1006/excr.1995.1105

[dmae007-B91] Jang YJ , Kim M, Lee BK, Kim J. Induction of human trophoblast stem-like cells from primed pluripotent stem cells. Proc Natl Acad Sci USA 2022;119:e2115709119. 35537047 10.1073/pnas.2115709119 PMC9171790

[dmae007-B92] Jenkins G. The role of proteases in transforming growth factor-β activation. Int J Biochem Cell Biol 2008;40:1068–1078. 18243766 10.1016/j.biocel.2007.11.026

[dmae007-B93] Johnson EL , Chakraborty R. Placental Hofbauer cells limit HIV-1 replication and potentially offset mother to child transmission (MTCT) by induction of immunoregulatory cytokines. Retrovirology 2012;9:101–111. 23217137 10.1186/1742-4690-9-101 PMC3524025

[dmae007-B94] Jones RL , Findlay JK, Farnworth PG, Robertson DM, Wallace E, Salamonsen LA. Activin A and inhibin A differentially regulate human uterine matrix metalloproteinases: potential interactions during decidualization and trophoblast invasion. Endocrinology2006a;147:724–732. 16282351 10.1210/en.2005-1183

[dmae007-B95] Jones RL , Stoikos C, Findlay JK, Salamonsen LA. TGF-β superfamily expression and actions in the endometrium and placenta. Reproduction2006b;132:217–232. 16885531 10.1530/rep.1.01076

[dmae007-B96] Kamato D , Bich Do H, Osman N, Ross BP, Mohamed R, Xu S, Little PJ. Smad linker region phosphorylation is a signalling pathway in its own right and not only a modulator of canonical TGF-β signalling. Cell Mol Life Sci 2020;77:243–251. 31407020 10.1007/s00018-019-03266-3 PMC11104920

[dmae007-B97] Kapustin RV , Drobintseva AO, Alekseenkova EN, Onopriychuk AR, Arzhanova ON, Polyakova VO, Kvetnoy IM. Placental protein expression of kisspeptin-1 (KISS1) and the kisspeptin-1 receptor (KISS1R) in pregnancy complicated by diabetes mellitus or preeclampsia. Arch Gynecol Obstet 2020;301:437–445. 31811415 10.1007/s00404-019-05408-1

[dmae007-B98] Karmakar S , Das C. Regulation of trophoblast invasion by IL-1β and TGF-β1. Am J Reprod Immunol 2002;48:210–219. 12516631 10.1034/j.1600-0897.2002.01151.x

[dmae007-B99] Karvas RM , Khan SA, Verma S, Yin Y, Kulkarni D, Dong C, Park K, Mi Chew B, Sane E, Fischer LA et al Stem-cell-derived trophoblast organoids model human placental development and susceptibility to emerging pathogens. Cell Stem Cell 2022;29:810–825.e8. 35523141 10.1016/j.stem.2022.04.004 PMC9136997

[dmae007-B100] Keelan JA , Groome NP, Mitchell MD. Regulation of activin-A production by human amnion, decidua and placenta *in vitro* by pro-inflammatory cytokines. Placenta 1998;19:429–434. 9699965 10.1016/s0143-4004(98)90084-4

[dmae007-B101] Keskin DB , Allan DSJ, Rybalov B, Andzelm MM, Stern JNH, Kopcow HD, Koopman LA, Strominger JL. TGFβ promotes conversion of CD16+ peripheral blood NK cells into CD16-NK cells with similarities to decidual NK cells. Proc Natl Acad Sci USA 2007;104:3378–3383. 17360654 10.1073/pnas.0611098104 PMC1805591

[dmae007-B102] Knöfler M , Haider S, Saleh L, Pollheimer J, Gamage TKJB, James J. Human placenta and trophoblast development: key molecular mechanisms and model systems. Cell Mol Life Sci 2019;76:3479–3496. 31049600 10.1007/s00018-019-03104-6 PMC6697717

[dmae007-B103] Knöfler M , Pollheimer J. Human placental trophoblast invasion and differentiation: a particular focus on Wnt signaling. Front Genet 2013;4:190. 24133501 10.3389/fgene.2013.00190 PMC3783976

[dmae007-B104] Kobayashi T , Lyons KM, McMahon AP, Kronenberg HM. BMP signaling stimulates cellular differentiation at multiple steps during cartilage development. Proc Natl Acad Sci USA 2005;102:18023–18027. 16322106 10.1073/pnas.0503617102 PMC1312369

[dmae007-B105] Koel M , Võsa U, Krjutškov K, Einarsdottir E, Kere J, Tapanainen J, Katayama S, Ingerpuu S, Jaks V, Stenman UH et al Optimizing bone morphogenic protein 4-mediated human embryonic stem cell differentiation into trophoblast-like cells using fibroblast growth factor 2 and transforming growth factor-β/activin/nodal signalling inhibition. Reprod Biomed Online 2017;35:253–263. 28647356 10.1016/j.rbmo.2017.06.003

[dmae007-B106] Kojima J , Fukuda A, Taira H, Kawasaki T, Ito H, Kuji N, Isaka K, Umezawa A, Akutsu H. Efficient production of trophoblast lineage cells from human induced pluripotent stem cells. Lab Invest 2017;97:1188–1200. 28287635 10.1038/labinvest.2016.159

[dmae007-B107] Kokkinos MI , Murthi P, Wafai R, Thompson EW, Newgreen DF. Cadherins in the human placenta—epithelial–mesenchymal transition (EMT) and placental development. Placenta 2010;31:747–755. 20659767 10.1016/j.placenta.2010.06.017

[dmae007-B108] Krop J , Heidt S, Claas FHJ, Eikmans M. Regulatory T cells in pregnancy: it is not all about FoxP3. Front Immunol 2020;11:1182. 32655556 10.3389/fimmu.2020.01182 PMC7324675

[dmae007-B109] Kubiczkova L , Sedlarikova L, Hajek R, Sevcikova S. TGF-β – an excellent servant but a bad master. J Transl Med 2012;10:1–24. 22943793 10.1186/1479-5876-10-183 PMC3494542

[dmae007-B110] Kupper N , Pritz E, Siwetz M, Guettler J, Huppertz B. Placental villous explant culture 2.0: flow culture allows studies closer to the in vivo situation. Int J Mol Sci 2021;22:7464. 34299084 10.3390/ijms22147464 PMC8308011

[dmae007-B111] Laiho M , Saksela O, Keski-Oja J. Transforming growth factor-beta induction of type-1 plasminogen activator inhibitor. Pericellular deposition and sensitivity to exogenous urokinase. J Biol Chem 1987;262:17467–17474. 3121597

[dmae007-B112] Lash GE , Otun HA, Innes BA, Bulmer JN, Searle RF, Robson SC. Inhibition of trophoblast cell invasion by TGFB1, 2, and 3 is associated with a decrease in active proteases. Biol Reprod 2005;73:374–381. 15858216 10.1095/biolreprod.105.040337

[dmae007-B113] Lash GE , Schiessl B, Kirkley M, Innes BA, Cooper A, Searle RF, Robson SC, Bulmer JN. Expression of angiogenic growth factors by uterine natural killer cells during early pregnancy. J Leukoc Biol 2006;80:572–580. 16816146 10.1189/jlb.0406250

[dmae007-B114] Laverty HG , Wakefield LM, Occleston NL, O’Kane S, Ferguson MWJ. TGF-β3 and cancer: a review. Cytokine Growth Factor Rev 2009;20:305–317. 19656717 10.1016/j.cytogfr.2009.07.002 PMC7294566

[dmae007-B115] Lawera A , Tong Z, Thorikay M, Redgrave RE, Cai J, van Dinther M, Morrell NW, Afink GB, Charnock-Jones DS, Arthur HM et al Role of soluble endoglin in BMP9 signaling. Proc Natl Acad Sci USA 2019;116:17800–17808. 31431534 10.1073/pnas.1816661116 PMC6731690

[dmae007-B116] Leaños-Miranda A , Navarro-Romero CS, Sillas-Pardo LJ, Ramírez-Valenzuela KL, Isordia-Salas I, Jiménez-Trejo LM. Soluble endoglin as a marker for preeclampsia, its severity, and the occurrence of adverse outcomes. Hypertension 2019;74:991–997. 31446801 10.1161/HYPERTENSIONAHA.119.13348

[dmae007-B117] Leavey K , Benton SJ, Grynspan D, Kingdom JC, Bainbridge SA, Cox BJ. Unsupervised placental gene expression profiling identifies clinically relevant subclasses of human preeclampsia. Hypertension 2016;68:137–147. 27160201 10.1161/HYPERTENSIONAHA.116.07293

[dmae007-B118] Lebrin F , Deckers M, Bertolino P, Ten Dijke P. TGF-β receptor function in the endothelium. Cardiovasc Res 2005;65:599–608. 15664386 10.1016/j.cardiores.2004.10.036

[dmae007-B119] Lebrin F , Goumans MJ, Jonker L, Carvalho RLC, Valdimarsdottir G, Thorikay M, Mummery C, Arthur HM, Ten Dijke P. Endoglin promotes endothelial cell proliferation and TGF-β/ALK1 signal transduction. EMBO J 2004;23:4018–4028. 15385967 10.1038/sj.emboj.7600386 PMC524335

[dmae007-B120] Lee JS , Romero R, Han YM, Kim HC, Kim CJ, Hong JS, Huh D. Placenta-on-a-chip: a novel platform to study the biology of the human placenta. J Matern Fetal Neonatal Med 2016;29:1046–1054. 26075842 10.3109/14767058.2015.1038518 PMC5625348

[dmae007-B121] Li H , Cao R, Bai L, Qiao XM, Zhao YQ. Lefty promotes the proliferation and invasion of trophoblast cells by inhibiting nodal expression. Cell Biol Int 2018;42:1259–1264. 29663570 10.1002/cbin.10976

[dmae007-B122] Li H , Peng H, Hong W, Wei Y, Tian H, Huang X, Jia L, Zheng J, Duan T, He Q et al Human placental endothelial cell and trophoblast heterogeneity and differentiation revealed by single-cell RNA sequencing. Cells2023a;12:87. 10.3390/cells12010087 PMC9818681 36611882

[dmae007-B123] Li S , Wang Y, Cao B, Wu Y, Ji L, Li YX, Liu M, Zhao Y, Qiao J, Wang H et al Maturation of growth differentiation factor 15 in human placental trophoblast cells depends on the interaction with matrix metalloproteinase-26. J Clin Endocrinol Metab2014a;99:E2277–E2287. 25093616 10.1210/jc.2014-1598

[dmae007-B124] Li X , Li ZH, Wang YX, Liu TH. A comprehensive review of human trophoblast fusion models: recent developments and challenges. Cell Death Discov2023b;9:1–14. 37816723 10.1038/s41420-023-01670-0 PMC10564767

[dmae007-B125] Li X , Liu L, Whitehead C, Li J, Thierry B, Le TD, Winter M. Identifying preeclampsia-associated genes using a control theory method. Brief Funct Genomics 2022;21:296–309. 35484822 10.1093/bfgp/elac006 PMC9328024

[dmae007-B126] Li XL , Chen TT, Dong X, Gou WL, Lau S, Stone P, Chen Q. Early onset preeclampsia in subsequent pregnancies correlates with early onset preeclampsia in first pregnancy. Eur J Obstet Gynecol Reprod Biol2014b;177:94–99. 24784713 10.1016/j.ejogrb.2014.03.043

[dmae007-B127] Li Y , Klausen C, Cheng JC, Zhu H, Leung PCK. Activin A, B, and AB increase human trophoblast cell invasion by up-regulating N-cadherin. J Clin Endocrinol Metab2014c;99:E2216–E2225. 25105734 10.1210/jc.2014-2118

[dmae007-B128] Li Y , Klausen C, Zhu H, Leung PCK. Activin A increases human trophoblast invasion by inducing SNAIL-mediated MMP2 up-regulation through ALK4. J Clin Endocrinol Metab2015a;100:E1415–E1427. 26305619 10.1210/jc.2015-2134

[dmae007-B129] Li Y , Yan J, Chang HM, Chen ZJ, Leung PCK. Roles of TGF-β superfamily proteins in extravillous trophoblast invasion. Trends Endocrinol Metab 2021;32:170–189. 33478870 10.1016/j.tem.2020.12.005

[dmae007-B130] Li Y , Zhao YJ, Zou QY, Zhang K, Wu YM, Zhou C, Wang K, Zheng J. Preeclampsia does not alter vascular growth and expression of CD31 and vascular endothelial cadherin in human placentas. J Histochem Cytochem2015b;63:22–31. 25362142 10.1369/0022155414558063 PMC4395995

[dmae007-B131] Librach CL , Feigenbaum SL, Bass KE, Cui TY, Verastas N, Sadovsky Y, Quigley JP, French DL, Fisher SJ. Interleukin-1 beta regulates human cytotrophoblast metalloproteinase activity and invasion in vitro. J Biol Chem 1994;269:17125–17131. 8006017

[dmae007-B132] Lin C , He H, Cui N, Ren Z, Zhu M, Khalil RA. Decreased uterine vascularization and uterine arterial expansive remodeling with reduced matrix metalloproteinase-2 and -9 in hypertensive pregnancy. Am J Physiol Heart Circ Physiol 2020;318:H165–H180. 31834839 10.1152/ajpheart.00602.2019 PMC6985805

[dmae007-B133] Liu E , Liu Z, Zhou Y, Chen M, Wang L, Li J. MicroRNA-142-3p inhibits trophoblast cell migration and invasion by disrupting the TGF-β1/Smad3 signaling pathway. Mol Med Rep 2019;49:3775–3782. 10.3892/mmr.2019.9997 30864732

[dmae007-B134] Liu X , Wang G, Huang H, Lv X, Si Y, Bai L, Wang G, Li Q, Yang W. Exploring maternal-fetal interface with in vitro placental and trophoblastic models. Front Cell Dev Biol 2023;11:1279227. 38033854 10.3389/fcell.2023.1279227 PMC10682727

[dmae007-B135] Liu Y , Gao S, Zhao Y, Wang H, Pan Q, Shao Q. Decidual natural killer cells: a good nanny at the maternal-fetal interface during early pregnancy. Front Immunol 2021;12:663660. 34054831 10.3389/fimmu.2021.663660 PMC8149889

[dmae007-B136] Loewendorf AI , Nguyen TA, Yesayan MN, Kahn DA. Preeclampsia is characterized by fetal NK cell activation and a reduction in regulatory T cells. Am J Reprod Immunol 2015;74:258–267. 25962852 10.1111/aji.12393 PMC5008194

[dmae007-B137] Lokki AI , Daly E, Triebwasser M, Kurki MI, Roberson EDO, Häppölä P, Auro K, Perola M, Heinonen S, Kajantie E et al Protective low-frequency variants for preeclampsia in the Fms related tyrosine kinase 1 gene in the Finnish population. Hypertension 2017;70:365–371. 28652462 10.1161/HYPERTENSIONAHA.117.09406 PMC5535812

[dmae007-B138] Luo K. Signaling cross talk between TGF-β/Smad and other signaling pathways. Cold Spring Harb Perspect Biol 2017;9:a022137. 27836834 10.1101/cshperspect.a022137 PMC5204325

[dmae007-B139] Lyall F , Robson SC, Bulmer JN. Spiral artery remodeling and trophoblast invasion in preeclampsia and fetal growth restriction relationship to clinical outcome. Hypertension 2013;62:1046–1054. 24060885 10.1161/HYPERTENSIONAHA.113.01892

[dmae007-B140] Lyall F , Simpson H, Bulmer JN, Barber A, Robson SC. Transforming growth factor-β expression in human placenta and placental bed in third trimester normal pregnancy, preeclampsia, and fetal growth restriction. Am J Pathol 2001;159:1827–1838. 11696443 10.1016/s0002-9440(10)63029-5 PMC1867050

[dmae007-B141] Ma J , Sanchez-Duffhues G, Goumans MJ, ten Dijke P. TGF-β-induced endothelial to mesenchymal transition in disease and tissue engineering. Front Cell Dev Biol2020a;8:260. 32373613 10.3389/fcell.2020.00260 PMC7187792

[dmae007-B142] Ma J , van der Zon G, Gonçalves MAFV, van Dinther M, Thorikay M, Sanchez-Duffhues G, ten Dijke P. TGF-β-induced endothelial to mesenchymal transition is determined by a balance between SNAIL and ID factors. Front Cell Dev Biol 2021;9:616610. 33644053 10.3389/fcell.2021.616610 PMC7907445

[dmae007-B143] Ma Y , Yang Q, Fan M, Zhang L, Gu Y, Jia W, Li Z, Wang F, Li Y, Xia Wang J et al Placental endovascular extravillous trophoblasts (enEVTs) educate maternal T-cell differentiation along the maternal-placental circulation. Cell Prolif2020b;53:e12802. 32291850 10.1111/cpr.12802 PMC7260064

[dmae007-B144] Makris A , Yeung KR, Lim SM, Sunderland N, Heffernan S, Thompson JF, Iliopoulos J, Killingsworth MC, Yong J, Xu B et al Placental growth factor reduces blood pressure in a uteroplacental ischemia model of preeclampsia in nonhuman primates. Hypertension 2016;67:1263–1272. 27091894 10.1161/HYPERTENSIONAHA.116.07286 PMC4867111

[dmae007-B145] Manuelpillai U , Schneider-Kolsky M, Dole A, Wallace EM. Activin A and activin receptors in gestational tissue from preeclamptic pregnancies. J Endocrinol 2001;171:57–64. 11572790 10.1677/joe.0.1710057

[dmae007-B146] Manuelpillai U , Schneider-Kolsky M, Thirunavukarasu P, Dole A, Waldron K, Wallace EM. Effect of hypoxia on placental activin a, inhibin a and follistatin synthesis. Placenta 2003;24:77–83. 12495662 10.1053/plac.2002.0870

[dmae007-B147] Margioula-Siarkou G , Margioula-Siarkou C, Petousis S, Margaritis K, Vavoulidis E, Gullo G, Alexandratou M, Dinas K, Sotiriadis A, Mavromatidis G. The role of endoglin and its soluble form in pathogenesis of preeclampsia. Mol Cell Biochem 2021;477:479–491. 34783962 10.1007/s11010-021-04294-z

[dmae007-B148] Marjono AB , Brown DA, Horton KE, Wallace EM, Breit SN, Manuelpillai U. Macrophage inhibitory cytokine-1 in gestational tissues and maternal serum in normal and pre-eclamptic pregnancy. Placenta 2003;24:100–106. 12495665 10.1053/plac.2002.0881

[dmae007-B149] Marshall SA , Hannan NJ, Jelinic M, Nguyen TPH, Girling JE, Parry LJ. Animal models of preeclampsia: translational failings and why. Am J Physiol Regul Integr Comp Physiol 2018;314:R499–R508. 29212809 10.1152/ajpregu.00355.2017

[dmae007-B150] Matsuzaki K. Smad phosphoisoform signaling specificity: the right place at the right time. Carcinogenesis 2011;32:1578–1588. 21798854 10.1093/carcin/bgr172 PMC3204345

[dmae007-B151] Meekins JW , Pijnenborg R, Hanssens M, MCFadyen IR, van Asshe A. A study of placental bed spiral arteries and trophoblast invasion in normal and severe pre-eclamptic pregnancies. Br J Obstet Gynaecol 1994;101:669–674. 7947500 10.1111/j.1471-0528.1994.tb13182.x

[dmae007-B152] Mercnik MH , Schliefsteiner C, Fluhr H, Wadsack C. Placental macrophages present distinct polarization pattern and effector functions depending on clinical onset of preeclampsia. Front Immunol 2022;13:1095879. 36713449 10.3389/fimmu.2022.1095879 PMC9878680

[dmae007-B153] Mezouar S , Katsogiannou M, Ben Amara A, Bretelle F, Mege JL. Placental macrophages: origin, heterogeneity, function and role in pregnancy-associated infections. Placenta 2021;103:94–103. 33120051 10.1016/j.placenta.2020.10.017 PMC7568513

[dmae007-B154] Miyazono K , Maeda S, Imamura T. BMP receptor signaling: transcriptional targets, regulation of signals, and signaling cross-talk. Cytokine Growth Factor Rev 2005;16:251–263. 15871923 10.1016/j.cytogfr.2005.01.009

[dmae007-B155] Moustakas A , Heldin CH. The regulation of TGFβ signal transduction. Development 2009;136:3699–3714. 19855013 10.1242/dev.030338

[dmae007-B156] Moustakas A , Pardali K, Gaal A, Heldin CH. Mechanisms of TGF-β signaling in regulation of cell growth and differentiation. Immunol Lett 2002;82:85–91. 12008039 10.1016/s0165-2478(02)00023-8

[dmae007-B157] Msheik H , El Hayek S, Bari MF, Azar J, Abou-Kheir W, Kobeissy F, Vatish M, Daoud G. Transcriptomic profiling of trophoblast fusion using BeWo and JEG-3 cell lines. Mol Hum Reprod 2019;25:811–824. 31778538 10.1093/molehr/gaz061

[dmae007-B158] Mullican SE , Lin-Schmidt X, Chin CN, Chavez JA, Furman JL, Armstrong AA, Beck SC, South VJ, Dinh TQ, Cash-Mason TD et al GFRAL is the receptor for GDF15 and the ligand promotes weight loss in mice and nonhuman primates. Nat Med 2017;23:1150–1157. 28846097 10.1038/nm.4392

[dmae007-B159] Munir S , Xu G, Wu Y, Yang B, Lala PK, Peng C. Nodal and ALK7 inhibit proliferation and induce apoptosis in human trophoblast cells. J Biol Chem 2004;279:31277–31286. 15150278 10.1074/jbc.M400641200

[dmae007-B160] Murray PJ , Allen JE, Biswas SK, Fisher EA, Gilroy DW, Goerdt S, Gordon S, Hamilton JA, Ivashkiv LB, Lawrence T et al Macrophage activation and polarization: nomenclature and experimental guidelines. Immunity 2014;41:14–20. 25035950 10.1016/j.immuni.2014.06.008 PMC4123412

[dmae007-B161] Murray PJ , Wynn TA. Obstacles and opportunities for understanding macrophage polarization. J Leukoc Biol 2011;89:557–563. 21248152 10.1189/jlb.0710409 PMC3058818

[dmae007-B162] Mylonas I , Schiessl B, Jeschke U, Vogl J, Makrigiannakis A, Kuhn C, Kunze S, Schulze S, Kainer F, Friese K. Expression of inhibin/activin subunits alpha (-α), beta A (-β A) and beta B (-β B) in placental tissue of normal and intrauterine growth restricted (IUGR) pregnancies. J Mol Histol 2006;37:43–52. 16670820 10.1007/s10735-006-9029-6

[dmae007-B163] Mylonas I , Schiessl B, Jeschke U, Vogl J, Makrigiannakis A, Kuhn C, Schulze S, Kainer F, Friese K. Expression of inhibin/activin subunits alpha (-α), betaA (-βA), and betaB (-βB) in placental tissue of normal, preeclamptic, and HELLP pregnancies. Endocr Pathol 2006;17:19–33. 16760577 10.1385/ep:17:1:19

[dmae007-B164] Nadeem L , Munir S, Fu G, Dunk C, Baczyk D, Caniggia I, Lye S, Peng C. Nodal signals through activin receptor-like kinase 7 to inhibit trophoblast migration and invasion: implication in the pathogenesis of preeclampsia. Am J Pathol 2011;178:1177–1189. 21356369 10.1016/j.ajpath.2010.11.066 PMC3069932

[dmae007-B165] Namwanje M , Brown CW. Activins and inhibins: roles in development, physiology, and disease. Cold Spring Harb Perspect Biol 2016;8:a021881. 27328872 10.1101/cshperspect.a021881 PMC4930927

[dmae007-B166] Nandi P , Siddiqui MF, Lala PK. Restraint of trophoblast invasion of the uterus by decorin: role in pre-eclampsia. Am J Reprod Immunol 2016;75:351–360. 26554635 10.1111/aji.12449

[dmae007-B167] Ning F , Liu H, Lash GE. The role of decidual macrophages during normal and pathological pregnancy. Am J Reprod Immunol 2016;75:298–309. 26750089 10.1111/aji.12477

[dmae007-B168] Nishi H , Nakada T, Hokamura M, Osakabe Y, Itokazu O, Huang LE, Isaka K. Hypoxia-inducible factor-1 transactivates transforming growth factor-β3 in trophoblast. Endocrinology 2004;145:4113–4118. 15155569 10.1210/en.2003-1639

[dmae007-B169] Novembri R , Funghi L, Voltolini C, Belmonte G, Vannuccini S, Torricelli M, Petraglia F. Placenta expresses anti-Müllerian hormone and its receptor: sex-related difference in fetal membranes. Placenta 2015;36:731–737. 25972076 10.1016/j.placenta.2015.04.009

[dmae007-B170] Oettel A , Lorenz M, Stangl V, Costa SD, Zenclussen AC, Schumacher A. Human umbilical vein endothelial cells foster conversion of CD4+ CD25− FOXP3− T cells into CD4+ FOXP3+ regulatory T cells via transforming growth factor-β. Sci Rep 2016;6:23278. 26987775 10.1038/srep23278 PMC4796866

[dmae007-B171] Ogasawara MS , Aoki K, Aoyama T, Katano K, Iinuma Y, Ozaki Y, Suzumori K. Elevation of transforming growth factor-beta1 is associated with recurrent miscarriage. J Clin Immunol 2000;20:453–457. 11202235 10.1023/a:1026459800016

[dmae007-B172] Oh SY , Song SE, Seo ES, Kim KH, Choi SJ, Suh YL, Sadovsky Y, Roh CR. The expression of connective tissue growth factor in pregnancies complicated by severe preeclampsia or fetal growth restriction. Placenta 2009;30:981–987. 19762080 10.1016/j.placenta.2009.08.006

[dmae007-B173] Okae H , Toh H, Sato T, Hiura H, Takahashi S, Shirane K, Kabayama Y, Suyama M, Sasaki H, Arima T. Derivation of human trophoblast stem cells. Cell Stem Cell 2018;22:50–63.e6. 29249463 10.1016/j.stem.2017.11.004

[dmae007-B174] Olsen OE , Skjærvik A, Størdal BF, Sundan A, Holien T. TGF-β contamination of purified recombinant GDF15. PLoS One 2017;12:e0187349. 29161287 10.1371/journal.pone.0187349 PMC5697882

[dmae007-B175] Parada-Niño L , Castillo-León LF, Morel A. Preeclampsia, natural history, genes, and miRNAs associated with the syndrome. J Pregnancy 2022;2022:3851225. 35198246 10.1155/2022/3851225 PMC8860533

[dmae007-B176] Park CB , DeMayo FJ, Lydon JP, Dufort D. NODAL in the uterus is necessary for proper placental development and maintenance of pregnancy. Biol Reprod 2012;86:194. 22378764 10.1095/biolreprod.111.098277 PMC4480152

[dmae007-B177] Park JY , Mani S, Clair G, Olson HM, Paurus VL, Ansong CK, Blundell C, Young R, Kanter J, Gordon S et al A microphysiological model of human trophoblast invasion during implantation. Nat Commun 2022;13:1–18. 35292627 10.1038/s41467-022-28663-4 PMC8924260

[dmae007-B178] Pastuschek J , Nonn O, Gutiérrez-Samudio RN, Murrieta-Coxca JM, Müller J, Sanft J, Huppertz B, Markert UR, Groten T, Morales-Prieto DM. Molecular characteristics of established trophoblast-derived cell lines. Placenta 2021;108:122–133. 33810901 10.1016/j.placenta.2021.02.022

[dmae007-B179] Pauklin S , Vallier L. Activin/Nodal signalling in stem cells. Development 2015;142:607–619. 25670788 10.1242/dev.091769

[dmae007-B180] Pavlov OV , Selutin AV, Pavlova OM, Selkov SA. Two patterns of cytokine production by placental macrophages. Placenta 2020;91:1–10. 31941612 10.1016/j.placenta.2020.01.005

[dmae007-B181] Peiris HN , Mitchell MD. The expression and potential functions of placental myostatin. Placenta 2012;33:902–907. 22818745 10.1016/j.placenta.2012.06.021

[dmae007-B182] Peiris HN , Salomon C, Payton D, Ashman K, Vaswani K, Chan A, Rice GE, Mitchell MD. Myostatin is localized in extravillous trophoblast and up-regulates migration. J Clin Endocrinol Metab 2014;99:E2288–E2297. 25093622 10.1210/jc.2014-2615

[dmae007-B1800] Peraçoli MTS, , Menegon FTF, , Borges VTM, , De Araújo Costa RA, , Thomazini-Santos IA, , Peraçoli JC. Platelet aggregation and TGF-beta(1) plasma levels in pregnant women with preeclampsia. J Reprod Immunol 2008;79:79–84. 18805591 10.1016/j.jri.2008.08.001

[dmae007-B183] Perucci LO , Gomes KB, Freitas LG, Godoi LC, Alpoim PN, Pinheiro MB, Miranda AS, Teixeira AL, Dusse LM, Sousa LP. Soluble endoglin, transforming growth factor-beta 1 and soluble tumor necrosis factor alpha receptors in different clinical manifestations of preeclampsia. PLoS One 2014;9:e97632. 24851923 10.1371/journal.pone.0097632 PMC4031102

[dmae007-B184] Petraglia F , Anceschi MM, Calza L, Garuti GC, Fusaro P, Giardino L, Genazzani AR, Vale W. Inhibin and activin in human fetal membranes: evidence for a local effect on prostaglandin release. J Clin Endocrinol Metab 1993;77:542–548. 8345060 10.1210/jcem.77.2.8345060

[dmae007-B185] Petraglia F , Gallinelli A, Grande A, Florio P, Ferrari S, Genazzani AR, Ling N, DePaolo LV. Local production and action of follistatin in human placenta. J Clin Endocrinol Metab 1994;78:205–210. 8288705 10.1210/jcem.78.1.8288705

[dmae007-B186] Petraglia F , Vaughan J, Vale W. Inhibin and activin modulate the release of gonadotropin-releasing hormone, human chorionic gonadotropin, and progesterone from cultured human placental cells. Proc Natl Acad Sci USA 1989;86:5114–5117. 2662194 10.1073/pnas.86.13.5114 PMC297567

[dmae007-B187] Phipps EA , Thadhani R, Benzing T, Karumanchi SA. Pre-eclampsia: pathogenesis, novel diagnostics and therapies. Nat Rev Nephrol 2019;15:275–289. 30792480 10.1038/s41581-019-0119-6 PMC6472952

[dmae007-B188] Pijnenborg R , Anthony J, Davey DA, Rees A, Tiltman A, Vercruysse L, van Assche A. Placental bed spiral arteries in the hypertensive disorders of pregnancy. Br J Obstet Gynaecol 1991;98:648–655. 1883787 10.1111/j.1471-0528.1991.tb13450.x

[dmae007-B189] Poniatowski AA , Wojdasiewicz P, Gasik R, Szukiewicz D. Transforming growth factor beta family: insight into the role of growth factors in regulation of fracture healing biology and potential clinical applications. Mediators Inflamm 2015;2015:137823. 25709154 10.1155/2015/137823 PMC4325469

[dmae007-B190] Possomato-Vieira JS , Khalil RA. Mechanisms of endothelial dysfunction in hypertensive pregnancy and preeclampsia. Adv Pharmacol 2016;77:361–431. 27451103 10.1016/bs.apha.2016.04.008 PMC4965238

[dmae007-B191] Pryor-Koishi K , Nishizawa H, Kato T, Kogo H, Murakami T, Tsuchida K, Kurahashi H, Udagawa Y. Overproduction of the follistatin-related gene protein in the placenta and maternal serum of women with pre-eclampsia. BJOG 2007;114:1128–1137. 17617189 10.1111/j.1471-0528.2007.01425.x

[dmae007-B192] Quinn KH , Lacoursiere DY, Cui L, Bui J, Parast MM. The unique pathophysiology of early-onset severe preeclampsia: role of decidual T regulatory cells. J Reprod Immunol 2011;91:76–82. 21782252 10.1016/j.jri.2011.05.006

[dmae007-B193] Raguema N , Moustadraf S, Bertagnolli M. Immune and apoptosis mechanisms regulating placental development and vascularization in preeclampsia. Front Physiol 2020;11:98. 32116801 10.3389/fphys.2020.00098 PMC7026478

[dmae007-B194] Rana S , Lemoine E, Granger J, Karumanchi SA. Preeclampsia: pathophysiology, challenges, and perspectives. Circ Res 2019;124:1094–1112. 30920918 10.1161/CIRCRESAHA.118.313276

[dmae007-B195] Redline RW , Patterson P. Pre-eclampsia is associated with an excess of proliferative immature intermediate trophoblast. Hum Pathol 1995;26:594–600. 7774887 10.1016/0046-8177(95)90162-0

[dmae007-B196] Reissmann E , Jörnvall H, Blokzijl A, Andersson O, Chang C, Minchiotti G, Persico MG, Ibáñez CF, Brivanlou AH. The orphan receptor ALK7 and the Activin receptor ALK4 mediate signaling by nodal proteins during vertebrate development. Genes Dev 2001;15:2010–2022. 11485994 10.1101/gad.201801 PMC312747

[dmae007-B197] Reyes L , Golos TG. Hofbauer cells: their role in healthy and complicated pregnancy. Front Immunol 2018;9:2628. 30498493 10.3389/fimmu.2018.02628 PMC6249321

[dmae007-B198] Reyes L , Wolfe B, Golos T. Hofbauer cells: placental macrophages of fetal origin. Results Probl Cell Differ 2017;62:45–60. 28455705 10.1007/978-3-319-54090-0_3

[dmae007-B199] Riley SC , Leask R, Balfour C, Brennand JE, Groome NP. Production of inhibin forms by the fetal membranes, decidua, placenta and fetus at parturition. Hum Reprod 2000;15:578–583. 10686199 10.1093/humrep/15.3.578

[dmae007-B200] Roberts RM , Ezashi T, Sheridan MA, Yang Y. Specification of trophoblast from embryonic stem cells exposed to BMP4. Biol Reprod 2018;99:212–224. 29579154 10.1093/biolre/ioy070 PMC6044404

[dmae007-B201] Roberts RM , Ezashi T, Temple J, Owen JR, Soncin F, Parast MM. The role of BMP4 signaling in trophoblast emergence from pluripotency. Cell Mol Life Sci 2022;79:1–22. 10.1007/s00018-022-04478-w PMC10243463 35877048

[dmae007-B202] Rochette L , Zeller M, Cottin Y, Vergely C. Insights into mechanisms of GDF15 and receptor GFRAL: therapeutic targets. Trends Endocrinol Metab 2020;31:939–951. 33172749 10.1016/j.tem.2020.10.004

[dmae007-B203] Roten LT , Johnson MP, Forsmo S, Fitzpatrick E, Dyer TD, Brennecke SP, Blangero J, Moses EK, Austgulen R. Association between the candidate susceptibility gene ACVR2A on chromosome 2q22 and pre-eclampsia in a large Norwegian population-based study (the HUNT study). Eur J Hum Genet 2008;17:250–257. 18781190 10.1038/ejhg.2008.158 PMC2696227

[dmae007-B204] Rudnicka E , Kunicki M, Calik-Ksepka A, Suchta K, Duszewska A, Smolarczyk K, Smolarczyk R. Anti-Müllerian hormone in pathogenesis, diagnostic and treatment of PCOS. Int J Mol Sci 2021;22:12507. 34830389 10.3390/ijms222212507 PMC8619458

[dmae007-B205] Runyan CE , Schnaper HW, Poncelet AC. The phosphatidylinositol 3-kinase/Akt pathway enhances Smad3-stimulated mesangial cell collagen i expression in response to transforming growth factor-β1. J Biol Chem 2004;279:2632–2639. 14610066 10.1074/jbc.M310412200

[dmae007-B206] Sánchez-Duffhues G , García de Vinuesa A, van de Pol V, Geerts ME, de Vries MR, Janson SGT, van Dam H, Lindeman JH, Goumans MJ, ten Dijke P. Inflammation induces endothelial-to-mesenchymal transition and promotes vascular calcification through downregulation of BMPR2. J Pathol 2019;247:333–346. 30430573 10.1002/path.5193 PMC6590480

[dmae007-B207] Sarkar P , Randall SM, Collier TS, Nero A, Russell TA, Muddiman DC, Rao BM. Activin/nodal signaling switches the terminal fate of human embryonic stem cell-derived trophoblasts. J Biol Chem 2015;290:8834–8848. 25670856 10.1074/jbc.M114.620641 PMC4423675

[dmae007-B208] Sasaki Y , Darmochwal-Kolarz D, Suzuki D, Sakai M, Ito M, Shima T, Shiozaki A, Rolinski J, Saito S. Proportion of peripheral blood and decidual CD4+ CD25(bright) regulatory T cells in pre-eclampsia. Clin Exp Immunol 2007;149:139–145. 17459078 10.1111/j.1365-2249.2007.03397.x PMC1942015

[dmae007-B209] Schäffer L , Scheid A, Spielmann P, Breymann C, Zimmermann R, Meuli M, Gassmann M, Marti HH, Wenger RH. Oxygen-regulated expression of TGF-β3, a growth factor involved in trophoblast differentiation. Placenta 2003;24:941–950. 14580376 10.1016/s0143-4004(03)00166-8

[dmae007-B210] Schliefsteiner C , Ibesich S, Wadsack C. Placental Hofbauer cell polarization resists inflammatory cues in vitro. Int J Mol Sci 2020;21:736. 31979196 10.3390/ijms21030736 PMC7038058

[dmae007-B211] Schliefsteiner C , Peinhaupt M, Kopp S, Lögl J, Lang-Olip I, Hiden U, Heinemann A, Desoye G, Wadsack C. Human placental Hofbauer cells maintain an anti-inflammatory M2 phenotype despite the presence of gestational diabetes mellitus. Front Immunol 2017;8:888. 28824621 10.3389/fimmu.2017.00888 PMC5534476

[dmae007-B212] Schneider-Kolsky ME , Manuelpillai U, Waldron K, Dole A, Wallace EM. The distribution of activin and activin receptors in gestational tissues across human pregnancy and during labour. Placenta 2002;23:294–302. 11969340 10.1053/plac.2002.0787

[dmae007-B213] Segerer SE , Rieger L, Kapp M, Dombrowski Y, Müller N, Dietl J, Kämmerer U. MIC-1 (a multifunctional modulator of dendritic cell phenotype and function) is produced by decidual stromal cells and trophoblasts. Hum Reprod 2012;27:200–209. 22064648 10.1093/humrep/der358

[dmae007-B214] Shaarawy M , Meleigy ME, Rasheed K. Maternal serum transforming growth factor beta-2 in preeclampsia and eclampsia, a potential biomarker for the assessment of disease severity and fetal outcome. J Soc Gynecol Investig 2016;8:27–31. 11223354

[dmae007-B215] Sheridan MA , Fernando RC, Gardner L, Hollinshead MS, Burton GJ, Moffett A, Turco MY. Establishment and differentiation of long-term trophoblast organoid cultures from the human placenta. Nat Protoc 2020;15:3441–3463. 32908314 10.1038/s41596-020-0381-x

[dmae007-B216] Sheridan MA , Zhao X, Fernando RC, Gardner L, Perez-Garcia V, Li Q, Marsh SGE, Hamilton R, Moffett A, Turco MY. Characterization of primary models of human trophoblast. Development 2021;148:dev199749. 34651188 10.1242/dev.199749 PMC8602945

[dmae007-B217] Sieber C , Kopf J, Hiepen C, Knaus P. Recent advances in BMP receptor signaling. Cytokine Growth Factor Rev 2009;20:343–355. 19897402 10.1016/j.cytogfr.2009.10.007

[dmae007-B218] Sierra-Filardi E , Puig-Kröger A, Blanco FJ, Nieto C, Bragado R, Palomero MI, Bernabéu C, Vega MA, Corbí AL. Activin A skews macrophage polarization by promoting a proinflammatory phenotype and inhibiting the acquisition of anti-inflammatory macrophage markers. Blood 2011;117:5092–5101. 21389328 10.1182/blood-2010-09-306993

[dmae007-B219] Simpson H , Robson SC, Bulmer JN, Barber A, Lyall F. Transforming growth factor β expression in human placenta and placental bed during early pregnancy. Placenta 2002;23:44–58. 11869091 10.1053/plac.2001.0746

[dmae007-B220] Sohlberg E , Saghafian-Hedengren S, Bachmayer N, Hamad RR, Bremme K, Holmlund U. Pre-eclampsia affects cord blood NK cell expression of activation receptors and serum cytokine levels but not CB monocyte characteristics. Am J Reprod Immunol 2014;71:178–188. 24238151 10.1111/aji.12169

[dmae007-B221] Soncin F , Morey R, Bui T, Requena DF, Cheung VC, Kallol S, Kittle R, Jackson MG, Farah O, Chousal J et al Derivation of functional trophoblast stem cells from primed human pluripotent stem cells. Stem Cell Reports 2022;17:1303–1317. 35594858 10.1016/j.stemcr.2022.04.013 PMC9214048

[dmae007-B222] Song Y , Keelan J, France JT. Activin-A stimulates, while transforming growth factor β1 inhibits,chorionic gonadotrophin production and aromatase activity in cultured human placental trophoblasts. Placenta 1996;17:603–610. 8916209 10.1016/s0143-4004(96)80078-6

[dmae007-B223] Stepan H , Hund M, Andraczek T. Combining biomarkers to predict pregnancy complications and redefine preeclampsia. Hypertension 2020;75:918–926. 32063058 10.1161/HYPERTENSIONAHA.119.13763 PMC7098437

[dmae007-B224] Stewart A , Guan H, Yang K. BMP-3 promotes mesenchymal stem cell proliferation through the TGF-beta/activin signaling pathway. J Cell Physiol 2010;223:658–666. 20143330 10.1002/jcp.22064

[dmae007-B225] Sudheer S , Bhushan R, Fauler B, Lehrach H, Adjaye J. FGF inhibition directs BMP4-mediated differentiation of human embryonic stem cells to syncytiotrophoblast. Stem Cells Dev 2012;21:2987–3000. 22724507 10.1089/scd.2012.0099 PMC3475151

[dmae007-B226] Sugulle M , Dechend R, Herse F, Weedon-Fekjaer MS, Johnsen GM, Brosnihan KB, Anton L, Luft FC, Wollert KC, Kempf T et al Circulating and placental growth-differentiation factor 15 in preeclampsia and in pregnancy complicated by diabetes mellitus. Hypertension 2009;54:106–112. 19470878 10.1161/HYPERTENSIONAHA.109.130583 PMC4167791

[dmae007-B227] Svensson-Arvelund J , Mehta RB, Lindau R, Mirrasekhian E, Rodriguez-Martinez H, Berg G, Lash GE, Jenmalm MC, Ernerudh J. The human fetal placenta promotes tolerance against the semiallogeneic fetus by inducing regulatory T cells and homeostatic M2 macrophages. J Immunol 2015;194:1534–1544. 25560409 10.4049/jimmunol.1401536

[dmae007-B228] Tal R. The role of hypoxia and hypoxia-inducible factor-1alpha in preeclampsia pathogenesis. Biol Reprod 2012;87:134–135. 23034156 10.1095/biolreprod.112.102723

[dmae007-B229] Tannetta DS , Sargent IL, Linton EA, Redman CWG. Vitamins C and E inhibit apoptosis of cultured human term placenta trophoblast. Placenta 2008;29:680–690. 18653232 10.1016/j.placenta.2008.04.009

[dmae007-B230] Tejera E , Bernardes J, Rebelo I. Co-expression network analysis and genetic algorithms for gene prioritization in preeclampsia. BMC Med Genomics 2013;6:51–10. 24219996 10.1186/1755-8794-6-51 PMC3829810

[dmae007-B231] Than NG , Romero R, Tarca AL, Kekesi KA, Xu Y, Xu Z, Juhasz K, Bhatti G, Leavitt RJ, Gelencser Z et al Integrated systems biology approach identifies novel maternal and placental pathways of preeclampsia. Front Immunol 2018;9:1661. 30135684 10.3389/fimmu.2018.01661 PMC6092567

[dmae007-B232] Thulluru HK , Park C, Dufort D, Kleiverda G, Oudejans C, van Dijk M. Maternal nodal inversely affects NODAL and STOX1 expression in the fetal placenta. Front Genet 2013;4:170. 23986775 10.3389/fgene.2013.00170 PMC3753557

[dmae007-B233] Tilburgs T , Scherjon SA, van der Mast BJ, Haasnoot GW, Versteeg-V D, Voort-Maarschalk M, Roelen DL, van Rood JJ, Claas FHJ. Fetal–maternal HLA-C mismatch is associated with decidual T cell activation and induction of functional T regulatory cells. J Reprod Immunol 2009;82:148–157. 19631389 10.1016/j.jri.2009.05.003

[dmae007-B234] Tranquilli AL , Brown MA, Zeeman GG, Dekker G, Sibai BM. The definition of severe and early-onset preeclampsia. Statements from the International Society for the Study of Hypertension in Pregnancy (ISSHP). Pregnancy Hypertens 2013;3:44–47. 26105740 10.1016/j.preghy.2012.11.001

[dmae007-B235] Tse WK , Whitley GSJ, Cartwright JE. Transforming growth factor-β1 regulates hepatocyte growth factor-induced trophoblast motility and invasion. Placenta 2002;23:699–705. 12398809 10.1016/s0143-4004(02)90866-0

[dmae007-B236] Tsuchida K , Nakatani M, Hitachi K, Uezumi A, Sunada Y, Ageta H, Inokuchi K. Activin signaling as an emerging target for therapeutic interventions. Cell Commun Signal 2009;7:1–11. 19538713 10.1186/1478-811X-7-15 PMC2713245

[dmae007-B237] Turco MY , Gardner L, Kay RG, Hamilton RS, Prater M, Hollinshead MS, McWhinnie A, Esposito L, Fernando R, Skelton H et al Trophoblast organoids as a model for maternal–fetal interactions during human placentation. Nature 2018;564:263–267. 30487605 10.1038/s41586-018-0753-3 PMC7220805

[dmae007-B238] Tzavlaki K , Moustakas A. TGF-β signaling. Biomolecules 2020;10:487. 32210029 10.3390/biom10030487 PMC7175140

[dmae007-B239] Valensise H , Vasapollo B, Gagliardi G, Novelli GP. Early and late preeclampsia. Hypertension 2008;52:873–880. 18824660 10.1161/HYPERTENSIONAHA.108.117358

[dmae007-B240] van Caam A , Madej W, Garcia de Vinuesa A, Goumans MJ, ten Dijke P, Blaney Davidson E, van der Kraan P. TGFβ1-induced SMAD2/3 and SMAD1/5 phosphorylation are both ALK5-kinase-dependent in primary chondrocytes and mediated by TAK1 kinase activity. Arthritis Res Ther 2017;19:112. 28569204 10.1186/s13075-017-1302-4 PMC5452635

[dmae007-B241] Vanwijk MJ , Kublickiene K, Boer K, VanBavel E. Vascular function in preeclampsia. Cardiovasc Res 2000;47:38–48. 10869528 10.1016/s0008-6363(00)00087-0

[dmae007-B242] Venkatesha S , Toporsian M, Lam C, Hanai JI, Mammoto T, Kim YM, Bdolah Y, Lim KH, Yuan HT, Libermann TA et al Soluble endoglin contributes to the pathogenesis of preeclampsia. Nat Med 2006;12:642–649. 16751767 10.1038/nm1429

[dmae007-B243] Vogt J , Traynor R, Sapkota GP. The specificities of small molecule inhibitors of the TGFß and BMP pathways. Cell Signal 2011;23:1831–1842. 21740966 10.1016/j.cellsig.2011.06.019

[dmae007-B244] von Dadelszen P , Magee LA, Roberts JM. Subclassification of preeclampsia. Hypertens Pregnancy 2009;22:143–148. https://doi.org/101081/PRG-120021060 10.1081/PRG-120021060 12908998

[dmae007-B245] Vondra S , Höbler AL, Lackner AI, Raffetseder J, Mihalic ZN, Vogel A, Saleh L, Kunihs V, Haslinger P, Wahrmann M et al The human placenta shapes the phenotype of decidual macrophages. Cell Rep 2023;42:111977. 36640334 10.1016/j.celrep.2022.111977

[dmae007-B246] Walker RG , Czepnik M, Goebel EJ, McCoy JC, Vujic A, Cho M, Oh J, Aykul S, Walton KL, Schang G et al Structural basis for potency differences between GDF8 and GDF11. BMC Biol 2017;15:19–22. 28257634 10.1186/s12915-017-0350-1 PMC5336696

[dmae007-B247] Walshe TE , Dole VS, Maharaj ASR, Patten IS, Wagner DD, D’Amore PA. Inhibition of VEGF or TGF-β signaling activates endothelium and increases leukocyte rolling. Arterioscler Thromb Vasc Biol 2009;29:1185–1192. 19461051 10.1161/ATVBAHA.109.186742 PMC2775449

[dmae007-B248] Wang HQ , Takebayashi K, Tsuchida K, Nishimura M, Noda Y. Follistatin-related gene (FLRG) expression in human endometrium: sex steroid hormones regulate the expression of FLRG in cultured human endometrial stromal cells. J Clin Endocrinol Metab 2003;88:4432–4439. 12970321 10.1210/jc.2002-021758

[dmae007-B249] Wang L , Wang H, Luo J, Xie T, Mor G, Liao A. Decorin promotes decidual M1-like macrophage polarization via mitochondrial dysfunction resulting in recurrent pregnancy loss. Theranostics 2022;12:7216–7236. 36438479 10.7150/thno.78467 PMC9691373

[dmae007-B250] Wang RN , Green J, Wang Z, Deng Y, Qiao M, Peabody M, Zhang Q, Ye J, Yan Z, Denduluri S et al Bone morphogenetic protein (BMP) signaling in development and human diseases. Genes Dis 2014;1:87–105. 25401122 10.1016/j.gendis.2014.07.005 PMC4232216

[dmae007-B251] Wang Y , Lewis DF, Gu Y, Zhang Y, Alexander JS, Granger DN. Placental trophoblast-derived factors diminish endothelial barrier function. J Clin Endocrinol Metab 2004;89:2421–2428. 15126573 10.1210/jc.2003-031707

[dmae007-B252] Wei X , Yang X. The central role of natural killer cells in preeclampsia. Front Immunol 2023;14:1009867. 36865565 10.3389/fimmu.2023.1009867 PMC9972679

[dmae007-B253] Wendt MK , Allington TM, Schiemann WP. Mechanisms of the epithelial-mesenchymal transition by TGF-β. Future Oncol 2009;5:1145–1168. 19852727 10.2217/fon.09.90 PMC2858056

[dmae007-B254] Wilczynski JR , Radwan M, Kalinka J. The characterization and role of regulatory T cells in immune reactions. Front Biosci 2008;13:2266–2274. 17981708 10.2741/2840

[dmae007-B255] Williams PJ , Searle RF, Robson SC, Innes BA, Bulmer JN. Decidual leucocyte populations in early to late gestation normal human pregnancy. J Reprod Immunol 2009;82:24–31. 19732959 10.1016/j.jri.2009.08.001

[dmae007-B256] Wójtowicz A , Zembala-Szczerba M, Babczyk D, Kołodziejczyk-Pietruszka M, Lewaczyńska O, Huras H. Early- and late-onset preeclampsia: a comprehensive cohort study of laboratory and clinical findings according to the new ISHHP criteria. Int J Hypertens 2019;2019:4108271. 31637053 10.1155/2019/4108271 PMC6766116

[dmae007-B257] Wrighton KH , Lin X, Feng XH. Phospho-control of TGF-β superfamily signaling. Cell Res 2009;19:8–20. 19114991 10.1038/cr.2008.327 PMC2929013

[dmae007-B258] Wu K , Liu F, Wu W, Chen Y, Zhang W. Bioinformatics approach reveals the critical role of TGF-β signaling pathway in pre-eclampsia development. Eur J Obstet Gynecol Reprod Biol 2019;240:130–138. 31280059 10.1016/j.ejogrb.2019.06.034

[dmae007-B259] Wu L , Luo LH, Zhang YX, Li Q, Xu B, Zhou GX, Luan HB, Liu YS. Alteration of Th17 and Treg cells in patients with unexplained recurrent spontaneous abortion before and after lymphocyte immunization therapy. Reprod Biol Endocrinol 2014;12:74. 25086467 10.1186/1477-7827-12-74 PMC4237930

[dmae007-B260] Wu Z , Fang L, Yang S, Gao Y, Wang Z, Meng Q, Dang X, Sun YP, Cheng JC. GDF-11 promotes human trophoblast cell invasion by increasing ID2-mediated MMP2 expression. Cell Commun Signal 2022;20:13. 35705978 10.1186/s12964-022-00899-z PMC9202197

[dmae007-B261] Xiao YT , Xiang LX, Shao JZ. Bone morphogenetic protein. Biochem Biophys Res Commun 2007;362:550–553. 17719560 10.1016/j.bbrc.2007.08.045

[dmae007-B262] Xie J , Zhu H, Chang HM, Klausen C, Dong M, Leung PCK. GDF8 promotes the cell invasiveness in human trophoblasts by upregulating the expression of follistatin-like 3 through the ALK5-SMAD2/3 signaling pathway. Front Cell Dev Biol 2020;8:573781. 33195207 10.3389/fcell.2020.573781 PMC7655915

[dmae007-B2600] Xu Y-T, , Shen M-H, , Jin A-Y, , Zhu R. Maternal circulating levels of transforming growth factor-β superfamily and its soluble receptors in hypertensive disorders of pregnancy. Int J Gynaecol Obstet 2017; 137:246–252. 10.1002/ijgo.12142 28281288

[dmae007-B263] Xu J , Sivasubramaniyam T, Yinon Y, Tagliaferro A, Ray J, Nevo O, Post M, Caniggia I. Aberrant TGFβ signaling contributes to altered trophoblast differentiation in preeclampsia. Endocrinology 2016;157:883–899. 26653761 10.1210/en.2015-1696

[dmae007-B264] Xu RH , Chen X, Li DS, Li R, Addicks GC, Glennon C, Zwaka TP, Thomson JA. BMP4 initiates human embryonic stem cell differentiation to trophoblast. Nat Biotechnol 2002;20:1261–1264. 12426580 10.1038/nbt761

[dmae007-B265] Xu XH , Jia Y, Zhou X, Xie D, Huang X, Jia L, Zhou Q, Zheng Q, Zhou X, Wang K. Downregulation of lysyl oxidase and lysyl oxidase-like protein 2 suppressed the migration and invasion of trophoblasts by activating the TGF-β/collagen pathway in preeclampsia. Exp Mol Med 2019;51:1–12. 10.1038/s12276-019-0211-9 PMC6389995 30804321

[dmae007-B266] Xuan YH , Choi YL, Shin YK, Ahn GH, Kim KH, Kim WJ, Lee HC, Kim SH. Expression of TGF-beta signaling proteins in normal placenta and gestational trophoblastic disease. Histol Histopathol 2007;22:227–234. 17163397 10.14670/HH-22.227

[dmae007-B267] Yagel S. The developmental role of natural killer cells at the fetal-maternal interface. Am J Obstet Gynecol 2009;201:344–350. 19788966 10.1016/j.ajog.2009.02.030

[dmae007-B268] Yair D , Eshed-Englender T, Kupferminc MJ, Geva E, Frenkel J, Sherman D. Serum levels of inhibin B, unlike inhibin A and activin A, are not altered in women with preeclampsia. Am J Reprod Immunol 2001;45:180–187. 11270644 10.1111/j.8755-8920.2001.450310.x

[dmae007-B269] Yan X , Liao H, Cheng M, Shi X, Lin X, Feng XH, Chen YG. Smad7 protein interacts with receptor-regulated Smads (R-Smads) to inhibit transforming growth factor-β (TGF-β)/Smad signaling. J Biol Chem 2016;291:382–392. 26555259 10.1074/jbc.M115.694281 PMC4697173

[dmae007-B270] Yan X , Liu Z, Chen Y. Regulation of TGF-β signaling by Smad7. Acta Biochim Biophys Sin (Shanghai) 2009;41:263–272. 19352540 10.1093/abbs/gmp018 PMC7110000

[dmae007-B271] Yan X , Xiong X, Chen Y-G. Feedback regulation of TGF-β signaling. Acta Biochim Biophys Sin (Shanghai) 2018;50:37–50. 29228156 10.1093/abbs/gmx129

[dmae007-B272] Yang D , Dai F, Yuan M, Zheng Y, Liu S, Deng Z, Tan W, Chen L, Zhang Q, Zhao X et al Role of transforming growth factor-β1 in regulating fetal-maternal immune tolerance in normal and pathological pregnancy. Front Immunol 2021;12:689181. 34531852 10.3389/fimmu.2021.689181 PMC8438197

[dmae007-B273] Yang L , Liang P, Yang H, Coyne CB. Trophoblast organoids with physiological polarity model placental structure and function. bioRxiv. 2023.01.12.523752, 2023, preprint: not peer reviewed. 10.1242/jcs.261528 PMC10499031 37676312

[dmae007-B274] Yao Y , Xu XH, Jin L. Macrophage polarization in physiological and pathological pregnancy. Front Immunol 2019;10:792. 31037072 10.3389/fimmu.2019.00792 PMC6476302

[dmae007-B275] Yeo CY , Whitman M. Nodal signals to Smads through Cripto-dependent and Cripto-independent mechanisms. Mol Cell 2001;7:949–957. 11389842 10.1016/s1097-2765(01)00249-0

[dmae007-B276] Yi Y , Zhu H, Klausen C, Chang HM, Inkster AM, Terry J, Leung PCK. Dysregulated BMP2 in the placenta may contribute to early-onset preeclampsia by regulating human trophoblast expression of extracellular matrix and adhesion molecules. Front Cell Dev Biol 2021;9:768669. 34970543 10.3389/fcell.2021.768669 PMC8712873

[dmae007-B277] Yong HEJ , Murthi P, Borg A, Kalionis B, Moses EK, Brennecke SP, Keogh RJ. Increased decidual mRNA expression levels of candidate maternal pre-eclampsia susceptibility genes are associated with clinical severity. Placenta 2014;35:117–124. 24331737 10.1016/j.placenta.2013.11.008 PMC4107207

[dmae007-B278] Yoshimura A , Wakabayashi Y, Mori T. Cellular and molecular basis for the regulation of inflammation by TGF-β. J Biochem 2010;147:781–792. 20410014 10.1093/jb/mvq043 PMC2912031

[dmae007-B279] You J , Wang W, Chang HM, Yi Y, Zhao H, Zhu H, Sun Y, Tang M, Wang C, Sang Y et al The BMP2 signaling axis promotes invasive differentiation of human trophoblasts. Front Cell Dev Biol 2021;9:607332. 33614644 10.3389/fcell.2021.607332 PMC7889606

[dmae007-B280] Yu L , Li D, Liao QP, Yang HX, Cao B, Fu G, Ye G, Bai Y, Wang H, Cui N et al High levels of activin A detected in preeclamptic placenta induce trophoblast cell apoptosis by promoting nodal signaling. J Clin Endocrinol Metab 2012;97:E1370–E1379. 22685232 10.1210/jc.2011-2729

[dmae007-B281] Zeng YT , Liu WF, Zheng PS, Li S. GDF15 deficiency hinders human trophoblast invasion to mediate pregnancy loss through downregulating Smad1/5 phosphorylation. iScience 2023;26:107902. 37766993 10.1016/j.isci.2023.107902 PMC10520888

[dmae007-B2800] Zhang L, , Li X, , Zhou C, , You Z, , Zhang J, , Cao G. The diagnosis values of serum STAT4 and sEng in preeclampsia. J Clin Lab Anal 2020;34:e23073. 31628681 10.1002/jcla.23073 PMC7031581

[dmae007-B282] Zhang J , Dunk CE, Shynlova O, Caniggia I, Lye SJ. TGFb1 suppresses the activation of distinct dNK subpopulations in preeclampsia. EBioMedicine 2019;39:531–539. 30579870 10.1016/j.ebiom.2018.12.015 PMC6355656

[dmae007-B283] Zhang YE. Non-Smad signaling pathways of the TGF-β family. Cold Spring Harb Perspect Biol 2017;9:a022129. 27864313 10.1101/cshperspect.a022129 PMC5287080

[dmae007-B284] Zhao HJ , Chang HM, Klausen C, Zhu H, Li Y, Leung PCK. Bone morphogenetic protein 2 induces the activation of WNT/β-catenin signaling and human trophoblast invasion through up-regulating BAMBI. Cell Signal2020a;67:109489. 31786181 10.1016/j.cellsig.2019.109489

[dmae007-B285] Zhao HJ , Chang HM, Zhu H, Klausen C, Li Y, Leung PCK. Bone morphogenetic protein 2 promotes human trophoblast cell invasion by inducing activin A production. Endocrinology2018a;159:2815–2825. 29846546 10.1210/en.2018-00301

[dmae007-B286] Zhao HJ , Klausen C, Li Y, Zhu H, Wang YL, Leung PCK. Bone morphogenetic protein 2 promotes human trophoblast cell invasion by upregulating N-cadherin via non-canonical SMAD2/3 signaling. Cell Death Dis2018b;9:1–12. 29416020 10.1038/s41419-017-0230-1 PMC5833391

[dmae007-B287] Zhao HJ , Klausen C, Zhu H, Chang HM, Li Y, Leung PCK. Bone morphogenetic protein 2 promotes human trophoblast cell invasion and endothelial-like tube formation through ID1-mediated upregulation of IGF binding protein-3. FASEB J2020b;34:3151–3164. 31908038 10.1096/fj.201902168RR

[dmae007-B288] Zhou C , Yan Q, Zou QY, Zhong XQ, Tyler CT, Magness RR, Bird IM, Zheng J. Sexual dimorphisms of preeclampsia-dysregulated transcriptomic profiles and cell function in fetal endothelial cells. Hypertension 2019;74:154–163. 31154903 10.1161/HYPERTENSIONAHA.118.12569 PMC6561818

[dmae007-B289] Zhu S , Li Z, Cui L, Ban Y, Leung PCK, Li Y, Ma J. Activin A increases human trophoblast invasion by upregulating integrin β1 through ALK4. FASEB J 2021;35:e21220. 33230889 10.1096/fj.202001604R PMC12315494

[dmae007-B290] Zou H , Niswander L. Requirement for BMP signaling in interdigital apoptosis and scale formation. Science 1996;272:738–741. 8614838 10.1126/science.272.5262.738

